# Findings from an opt-in eye examination service in English special schools. Is vision screening effective for this population?

**DOI:** 10.1371/journal.pone.0212733

**Published:** 2019-03-11

**Authors:** Lisa A. Donaldson, Marek Karas, Donna O’Brien, J. Margaret Woodhouse

**Affiliations:** 1 SeeAbility, Epsom, Surrey, United Kingdom; 2 School of Optometry & Vision Sciences, Cardiff University, Cardiff, United Kingdom; Faculty of Medicine, Cairo University, EGYPT

## Abstract

Our objective was to present the findings of an opt-in, school-based eye care service for children attending 11 special schools in England and use these findings to determine whether a vision screening programme would be appropriate for this population. Data from eye examinations provided to 949 pupils (mean age 10.7 years) was analysed to determine the prevalence and aetiology of visual deficiencies and reported eye care history. For 46.2% (n = 438) of pupils, a visual deficiency was recorded. 12.5% of all the children seen (n = 119) had a visual deficiency that was previously undiagnosed. Referral for a medical opinion was made for 3.1% (n = 29) of pupils seen by the service. Spectacle correction was needed for 31.5% (n = 299) of pupils; for 12.9% (122) these were prescribed for the first time. 3.7% (n = 11) of parents/carers of pupils needing spectacles chose not to use the spectacle dispensing service offered in school. Eye care history was available for 847 pupils (89.3%). Of the pupils for whom an eye care history was available, 44% (n = 373) reported no history of any previous eye care and10.7% (n = 91) reported a history of attending a community optical practice/opticians. Only one pupil from the school entry 4–5 age group (0.6% of age group n = 156) would have passed vision screening using current Public Health England screening guidelines. Children with a diagnosis of autism were significantly less likely to be able to provide a reliable measurement of visual acuity. This study supports previously published evidence of a very high prevalence of visual problems in children with the most complex needs and a significant unmet need in this group. It demonstrates routine school entry vision screening using current Public Health England guidelines is not appropriate for this group of children and very low uptake of community primary eye care services.

## Introduction

Sight is a critical sense for everyone and understanding a child’s visual abilities and limitations is vital to optimally support their development [1]. There is a body of evidence that children with learning disabilities are significantly more likely to experience eye and vision problems than members of the general childhood population [2–7]. Children with the most complex needs are likely to be taught in special schools. In England 79% of children with severe learning disabilities and 81% of children with profound and multiple learning disabilities attend such schools [8]. The total population attending special schools in England at the time of writing is estimated at 117,888 [9].

Vision screening in the general childhood population improves the identification and correction of reduced vision and is implemented in many countries worldwide [10]. Under the recommendations of the United Kingdom National Screening Committee [11], all children should have access to an orthoptic led screening of vision at school entry (age 4–5 years) primarily to identify amblyopia, but also refractive error and strabismus. However, it has been suggested that the provision of this type of screening, in special schools is patchy [5] and it is questionable if such screening is suitable for children with complex needs, given that many cannot be assessed using established visual acuity tools [4]. Studies [2,4,5] have also highlighted the low uptake of hospital eye care and primary care optometric eye examinations in community optical practices by the special schools population. The UK eye care professional bodies with SeeAbility have developed a recommended Framework for special schools eye care [12] which advocates the provision of routine eye care, including refraction, eye health checks and spectacle dispensing, in the special school environment. In 2017, Public Health England made resources available to support the commissioning and delivery of child vision screening and these recommend a routine full eye examination should be offered in school for all children attending a special school [13].

This study reports on service evaluation data from an opt-in school based eye care service provided by optometrists, orthoptists and dispensing opticians working with SeeAbility, a UK charity supporting people with learning disability and sight loss, across 11 special schools in England between 2013 and 2017. This evaluation sets out to report on uptake of the service, clinical findings, and self-reported eye care history of children attending at first appointment.

## Methods

Eye examinations were offered to children attending 11 special schools in London (8 schools), Buckinghamshire, Durham and Manchester. Informed parental consent (see S1 Fig.) was obtained prior to the eye care team visiting the school and parents were given a detailed questionnaire exploring previous ocular and general health history prior to the first visit [14],(see S2 Fig).

The questionnaire included 5 questions used to identify features of cerebral visual impairment (CVI) [15]. Cerebral visual impairment is an umbrella term for visual perceptual difficulties with a cerebral, rather than ocular origin. The cerebral deficit is often unknown, but manifestations of CVI are more common amongst children with a history of brain injury or maldevelopment. CVI can range from obvious poor sight to difficulties in specific tasks (such as negotiating steps) with good visual acuity. Children with a score of 3 or more were designated as suspect CVI and referred to a qualified teacher for the visually impaired (QTVI) for a more detailed inventory of questions where this service was available. Some children in the study had a diagnosis of CVI reported by parents or school; these were all children with recognisable poor sight.

In each case primary general health diagnosis was recorded as reported by parents or, when available, from school medical records.

During the 4 years since the service was launched, eye examinations were carried out by 6 optometrists, all experienced in working with children with disabilities. Examinations took place in suitable rooms of minimum 3 metres in length which could be blacked out and children were accompanied to the test by a member of teaching staff who knew them well and/or a parent. Refraction was conducted by retinoscopy, with the use of 1% cyclopentolate drops to produce cycloplegia when clinically necessary and practically possible in the judgement of the optometrist. Visual acuity was measured using available age and ability-appropriate tests. Tests available included Keeler Preferential Looking Cards [16] Cardiff Acuity Test[17], Bradford Visual Function Box[18] (in year 4 of operating our service), LogMAR linear and single crowded versions of Kay Pictures[19] and Keeler LogMAR Crowded letter test (formerly Glasgow Acuity Cards) [20].

For children starting reception year (at age 4–5) or nursery, a joint optometric/orthoptic assessment was carried out when possible, with visual acuity and binocular vision status being measured by an orthoptist. For all children, we attempted to measure vision and/or visual acuity monocularly and binocularly, with the pupil’s existing spectacles if worn. For all children, we attempted an assessment of binocular vision using tests including observation of corneal reflections, cover tests at distance and near, assessment of ocular motility, near point of convergence, 20/10 base out prism test, observation of smooth pursuit and saccadic eye movement ability, quality of fixation, stereopsis, and observation of visual reflexes (Optokinetic nystagmus, vestibulo-ocular reflex, blink). Pupil reactions, media quality, and an assessment of internal and external ocular health were attempted for all pupils.

Spectacles were prescribed when required. Parents or carers were given the choice of a spectacle dispensing service offered in school or being issued with a spectacle prescription voucher to use at a community optical practice.

Pupils were referred to their GP or for an ophthalmological opinion in secondary care according to clinical judgement.

A detailed lay language report form designed in collaboration with Ulster University School of Optometry and Vision Science, was completed for each child (S3 Fig)[21](. This was designed to clearly explain the findings of the assessment including the child’s visual abilities, recommended actions (including need for spectacles wear when necessary) and ongoing care plan. The report was designed to be easily incorporated into each child’s Education Health and Care Plan (EHCP); it was given to parents and school and was made available to Qualified Teachers of the Visually Impaired (QTVIs) when visual defects were identified. Reports were also shared when relevant and, depending on local arrangements with school paediatricians, speech and language teams and occupational therapists.

The data for this service evaluation was derived from annual audit of the SeeAbility service, which does not require ethical approval. Nevertheless, the proposed use of audit data was scrutinised and approved by the Cardiff School of Optometry and Vision Sciences Human Research Ethical Committee, prior to commencement of the service.

Our analysis of data was carried out using SPSS (IBM Corp., version 20.0.0.2) and was limited to the first examination of each pupil; this may have been performed over more than one contact during a period of a few weeks to gain as complete a clinical assessment as possible. Where children were absent, unwell or unable to complete their assessment, it was possible to rearrange appointments to allow maximally efficient use of clinician’s time.

The distribution of refractive error is influenced by ethnicity [22] and the schools covered by the SeeAbility service have a varied ethnic mix. The ethnicity of individual pupils participating in the service was not recorded at the time of testing. However, the ethnic diversity of the London schools is available, and figures for September 2016 across the eight participating London schools were: White European 34%, South Asian 12%, Black African Caribbean 32% and ‘other’ (East Asian, mixed or minority groups such as Afghan, Turkish etc.) 22%.

Anecdotal clinical observation indicated pupils with a diagnosis of autism were less likely to provide a reliable visual acuity measurement using the available tests than their peers with a learning disability of a different aetiology. We explored this in our data analysis by comparing ability to obtain a reliable visual acuity measurement in pupils with and without an autism diagnosis.

## Results

Over the four-year period 949 new children were seen; 509 were of primary school age and 440 secondary school age. Mean age was 10.7 years (range was 3.0 to 19.8 years).

### Location and uptake of service

Uptake of this opt-in service varied from year to year and from school to school.

At the end of academic year 2017, the mean uptake of the service was 81% (range 66–92%). Most of the children seen (n = 826, 87.0%) attended 9 schools in the south east of England, 8 of which are in the Greater London area (S4 Fig gives further detail). Very young children aged 2–3 years were not offered the service but in a few cases these younger children were seen when requested by the school or parents. The uptake rates exclude the 2-3yr olds.

### Clinical findings

#### Reported history of previous eye care

Table 1 shows the parent reported eye care history for pupils seen by the service. This information was available for 847 pupils (89.3%).

**Table 1:** Parent reported eye care history.

Previous Eye Care History	Number of Respondents (total n = 949)	Percentage of Total Pupils Seen by Service	Percentage of Respondents where eye care history known (n = 847)
Information not available	102	10.7%	NA
No previous eye care	373	39.3%	44.0%
Currently under hospital eye service	189	19.9%	22.3%
Discharged from hospital eye service, community follow up	20	2.1%	2.4%
Discharged from hospital eye service, no community follow up	188	19.9%	22.2%
No hospital eye service, seen by community optometrist	71	7.1%	8.4%
No hospital eye service, seen in school by optometrist	6	0.6%	0.7%

Of pupils for whom history was available 373 (44%) reported no previous eye care. Overall, 91 (10.7%) reported having attended a community optical practice. A further 6 pupils (0.7%) had been seen in school by an optometrist under special arrangements in which children were referred by a paediatrician.

#### Presenting level of vision

It was possible to assess every child for whom consent was received but often with only limited engagement in some elements of formal testing. In some cases, repeat attempts at assessment were necessary, or it was necessary to see children in their own classroom, a ball pool, corridor or sensory room.

A reliable measure of presenting visual acuity using a formal test was recorded for 574 (60.5%) pupils. This was defined as the acuity in the better eye or the binocular acuity when no monocular acuity was available. Low vision (WHO criterion [23] of LogMAR 0.5 or poorer in the better eye, or binocular) was recorded for 143 pupils (24.9% of the pupils for whom it was possible to record a presenting level of vision). This included 26 pupils with severe visual impairment and 12 pupils with CVI that precluded quantitative acuity measurement.

The recommended pass criteria for UK school vision screening for ages 4–5 years [13] is a monocular score of 0.2 LogMAR using the Keeler LogMAR crowded letter test. In the SeeAbility service, 156 pupils fell into this age group (4.00 to 5.97 years) but only 10 pupils were able to use a LogMAR crowded acuity type test (Kays Pictures LogMAR or Keeler LogMAR) and only one (0.6%) was able to pass the test using the recommended Keeler test.

Of the 574 pupils able to take part in acuity assessment and accepting binocular acuity in cases in which no monocular acuity was available, 300 (52.3%) pupils failed to achieve LogMAR 0.2 using any of the tests available. Using the more rigorous criterion of successful monocular acuity recording of LogMAR 0.2 or better in each eye, a total of 76.8% of special school pupils across all ages would fail vision screening.

Of the 375 pupils with no acuity score recorded, the reason for this was recorded for 348 pupils. Of these, 38 pupils had severe visual impairment or CVI and for 310 pupils co-operation / comprehension was too limited to achieve a reliable threshold result.

A diagnosis of disability was available for 855 pupils. For the purposes of analysis of ability to co-operate for visual acuity, the pupils were divided into those with a diagnosis of autism (444, 51.9%), and those with another diagnosis (such as Down’s syndrome, cerebral palsy, global developmental delay, specific syndromes etc., 411, 48.1%). Some children had a dual diagnosis of Down’s syndrome and autism and were categorized under autism. Pupils whose acuity was too poor to attempt acuity were excluded. Among the pupils who had autism, 61.7% were cooperative for acuity, while among pupils without autism, 68.9% were cooperative for acuity testing. The difference was significant (χ2 = 4.41, p = 0.04); children with autism were less likely to be successfully tested for acuity.

#### Incidence and type of refractive error requiring spectacle correction

An assessment of the level of refractive error was possible for 932 (98.2%) right eyes and 928 (97.8%) left eyes using the objective method of retinoscopy. Cyclopentolate 1% eye drops were used for 47 (5.0%) examinations. (Cyclopentolate is routinely used in paediatric ophthalmic assessment to measure refractive error by paralysing accommodation and to gain a good retinal view through a dilated pupil).

Table 2 shows the spectacle prescribing pattern of the service.

**Table 2:** Spectacle prescribing pattern.

Prescription advised	Number of pupils	Percentage of all students seen by service (n = 949)
No spectacles prescribed	646	68.1%
First spectacles prescribed	122	12.9%
Changed spectacle prescription	71	7.5%
Replacement spectacles due to poor condition	18	1.9%
No change to existing spectacles needed	88	9.3%
Spectacle wear discontinued	4	0.4%

Overall 646 (68.1%) pupils had either no refractive error or very low refractive error which did not indicate the need for spectacles; 299 (31.5%) children needed spectacles and for 122 (12.9%) these were prescribed for the first time, A very high proportion of the 299 parents /carers of children needing spectacles (n = 288, 96.3%) chose to have them dispensed and fitted in school.

Myopia of ≤-0.50 in either eye was measured in 212 pupils (22.3%); 38 pupils (4.0%) were high myopes (<-6.00D), including 8 children with myopia in excess of -13.00DS. The high myopes comprised 19 of primary school age and 19 of secondary age. Of this group of high myopes, 6 children were given spectacles for the first time by the service.

Hypermetropia of ≥+2.00 in either eye was measured in 144 (15.2%) pupils. Astigmatism was defined as ≥1.00DC in either eye and was noted for 271 (28.6%) pupils (astigmatism can be present in isolation or co-exist with myopia or hyperopia).

Accommodative accuracy is the ability to accurately focus onto a close target when corrected for any hyperopia, myopia or astigmatism and it is often impaired in children with learning disabilities [24,25,26]. This was assessed in 700 pupils: of these 572 (81.7%) had accurate accommodation and 74 (10.6%) had inaccurate accommodation. The children with under accommodation were prescribed spectacles incorporating a prescription for near vision, for 100% of these pupils, this was for the first time.

#### Incidence of strabismus and other problems with eye movement

A problem with eye movements/alignment (an orthoptic anomaly) was recorded in 239 (25.2%) of pupils; 214 (22.6%) had strabismus and 23 (2.4%) had nystagmus (involuntary eye movements). Other orthoptic anomalies recorded included poor eye movement control and lack of smooth pursuit eye movements.

#### Incidence of other pathology

A pathological condition was recorded in 72 (7.6%) pupils; some pupils had more than one condition. This included 4 cases of blepharitis (eyelash infection), 5 cases of conjunctivitis, 4 cases of keratoconus, 12 cases of cataract, 4 optic disc anomalies and 7 retinal anomalies.

#### Visual field defects and cerebral visual impairment (CVI)

Hemianopia is a total loss of one half of the vision in each eye, originating at a neural level; 13 cases were recorded, 5 of which were previously undiagnosed. A further 10 pupils had another gross visual field defect.

Cerebral visual impairment, a problem with visual processing at a cerebral level, was diagnosed or suspected in 141 (14.9%) pupils. All were referred to the QTVI for further evaluation.

#### Incidence of visual deficiency of any aetiology

A visual deficiency was recorded when a child had a defect of any aetiology (correctable refractive error, orthoptic anomaly, ocular pathology or suspected / confirmed cerebral visual impairment) irrespective of visual acuity scores. Results were available for 923 (97.3%) children; a decision would not be made for the remaining pupils after first assessment. Of these 923 pupils for whom it was possible to confirm the presence or absence of a visual deficiency 438 (47.5%) pupils had a visual deficiency and 119 of these (12.9%) were previously undiagnosed.

Of the 373 pupils with no history of previous eye care reported, 61 (16.4%) had a visual deficiency that was not previously known, 47 (12.6%) needed a spectacle prescription, 37 (9.9%) had low vision according to WHO criteria [23] including 5 pupils who had vision too low to be recorded (1.3%).

#### Onward referral

Twelve (1.3%) pupils were referred to their GP for further management and 17 (1.8%) for the opinion of an ophthalmologist. In addition 32 (3.4%) pupils were referred by the optometrist for an orthoptic opinion—this was provided in school by a SeeAbility orthoptist (this number discounts the new starters who received an orthoptic assessment as part of the service).

## Discussion

This evaluation shows that many children in our special school population have undiagnosed visual needs. Our findings align with those of previously published studies which reported the prevalence of ocular and visual anomalies among children with learning disabilities, including those in special schools elsewhere in the UK [2,4,5] and in other countries [3,6,7].

Previously published work has looked at children attending special schools in Wales [5], Scotland [2] and the north of England [4]. In the present study, 87.0% of pupils attended schools in the south east of England, mainly in the greater London area; thus it helps to inform the UK wide picture.

### Measurement of visual acuity

Despite the availability of a wide range of visual acuity tests, a reliable measurement of visual acuity using established methods was only possible for 60.5% of children. This is considerably lower than headline rates of cooperation reported by other studies [2,4,5]. Pilling [4] reported a rate of 97% cooperation with functional vision assessment which included the use of the Bradford box, a novel visual function instrument [18]. The rate of cooperation with standard tests (Crowded Keeler LogMAR, Crowded Kays pictures and Cardiff Acuity Test) was 62%, a similar proportion to that found in our evaluation. Woodhouse [5] reported successful acuity measurement in 96% of cases using Crowded Keeler LogMAR, Crowded Kays pictures and Cardiff Acuity Test with over half (54%) being able to complete the most challenging test, the Keeler Crowded LogMAR test and only 15 children refusing monocular assessment. Given that only 25 (2.6%) of our pupils were able to cooperate with the Keeler crowded test this would suggest we were assessing a less able population.

Routine school entry vision screening, as recommended by the UK National Screening Committee [11] relies on the ability to engage with standard acuity tests. In our evaluation only 1 child from the school entry 4–5 age group would have passed this screening test which demonstrates that routine school entry vision screening is not appropriate for this group of children.

Most currently available tests have been developed based on normal cognitive development and so are not optimal for this population which indicates a need for development of further tests specific to the learning-disabled child. As our findings show that children on the autistic spectrum were less likely to achieve a reliable acuity measurement, tests targeted to this group are clearly needed.

### Refractive error

Of all the vision deficiencies identified, the need for refractive error correction was the most prevalent with 31.5% of pupils needing spectacles, 12.9% for the first time. When compared to large population based studies [22], this study and previous studies of children with learning disabilities [2–7] indicate a significantly higher incidence of refractive error than in the general population. This further indicates the need for inclusion of refraction in any routine assessment.

For 98% of children we were able to carry out refraction using retinoscopy to determine refractive error and prescribe refractive correction when indicated. This strongly supports the routine use of retinoscopy as a more reliable means to identify refractive error than vision screening alone.

Significant uncorrected refractive error can mean a child is visually impaired in a functional sense, impairing their social, emotional and behavioural development and access to visual elements of their specialised education. Crucially a child may be unable to communicate poor vision if they are nonverbal. For example eye-gaze control technology allows the user to operate a laptop, computer or speech-generating device using movements of their eyes. Children with limited communication may be assessed for use of this technology and assumed cognitively unable to access it when simple correction of refractive error could allow them to do so. A systematic review by Williams et al [27] concluded that refractive error correction leads to functional improvement in children with neurodevelopmental impairment.

### Orthoptic anomalies

Strabismus and other orthoptic anomalies were the next most prevalent visual deficiency identified in this cohort of children with 25.2% of pupils having a problem with eye movements/alignment noted. Studies estimate the prevalence of childhood squint in the general population at 2.1% [28] making incidence 10 times higher in our cohort of children.

### Cerebral Visual Impairment (CVI)

14.9% (n = 141) of pupils had confirmed or suspected CVI, a significant cohort. It is extremely important that CVI is identified in children, so that presenting difficulties can be managed in an appropriate way, rather than being incorrectly assigned to a child’s learning disability.

### Other visual disorders

While other disorders were rarer, of note were the 4 children identified as having keratoconus. Keratoconus is a degenerative corneal condition which is relatively rare in the general population (incidence 0.27%) [29], but with a significantly higher incidence in the population of people with learning disabilities (5.5% in people with Down’s syndrome) [30]. The recently developed technique of corneal collagen cross-linking, can halt the progress of keratoconus [31] so timely intervention can make it possible to avoid the need for rigid contact lenses and, in later stages of the disease, a corneal transplant.

### Unrecognized vision problems

Overall there was a significant cohort of children within the schools who had a previously unrecognized visual deficiency identified (12.9% of the children seen for whom it was possible to confirm (n = 119)).

Of particular interest were the 373 children with no history of eye care; of these, 61 (16.4%) had a previously undiagnosed visual deficiency with 47 (12.6%) needing a first pair of spectacles.

Identification of sight problems in the general population is often symptom led [32] but many children with complex needs would not be able to self-report a change in vision or they may not recognise there is a problem. There is also the risk that behaviours secondary to poor vision may be wrongly attributed to the diagnosis of a learning disability, an example of diagnostic overshadowing, a common issue across all areas of healthcare for people with learning disabilities [33].

### Reported eye care history

Our results support existing evidence of poor uptake of primary and secondary eye care service by this group, with 44% of pupils for whom eye care history was available reporting no history of eye care. Only a small number of pupils, 91(10.7% of those for whom eye care history was known), had made use of community/high street optometry services. These services are free under the NHS England General Ophthalmic Service (GOS).

In our study, only 20 (9.6%) of the 208 pupils who had been discharged from hospital care had gone on to make use of these services. From this it could be hypothesized that children requiring refractive correction or adaptions to allow for low vision, once discharged or lost to follow up from a hospital eye clinic, may fail to access the correct spectacles or support. In light of the high incidence of refractive errors reported, this suggests significant unmet need. Given that having an eye test at an optometric practice is a free service for all children, these findings suggest that there must be considerable barriers for parents in accessing this community option.

The hospital eye service was the most frequently reported location for a child to have previously received eye care (46.9%, n = 397)). As it has been shown that failure to attend rates for hospital eye clinics are high for all children [34] and even higher for children with complex needs [35], it is likely that children currently under hospital care and requiring refractive correction or adaptions for low vision may get lost to follow up and may lose access to the correct support or spectacles.

### The benefits of service provision in the special school

Our findings confirm those of earlier studies that almost all children with learning disabilities can have an eye examination if the correct preparation is made [2,5]. Carrying out testing in school allows for minimal disruption to the school day and familiar surroundings which, in our experience, leads to better compliance with testing. Being in the school setting allows for the observation of children’s functional vision abilities in a familiar setting which may be the only visual assessment available when formal measures of vision are not possible. Furthermore, the ability to be flexible with appointments when children are unwell or not having a ‘good day’ in terms of tolerance or behaviour, means that there is effectively a near zero non-attendance rate.

Consent rates into the service were high (in 2016/17 academic year 81% (range 66–92%)) in the 9 schools where the whole school was offered the service). For 96.3% (n = 288) of pupils needing spectacles, families chose to have them dispensed and fitted in school. This suggests a service in school is well received and convenient for families.

In our experience, the presence of an eye care service in school allows for more effective dissemination of relevant information to teaching and support staff. As it is within the school setting, staff become familiar with the service as they attend examinations with their pupils and this allows for informal information sharing and increased awareness and understanding among teaching staff of vision related issues. More formally, visual abilities, needs and limitations are routinely included in a child’s Education Health and Care Plan (EHCP). Little et al [36] have evidenced that this visual information has previously been rarely mentioned. Working closely with the school ensures that pupils’ visual abilities and spectacle needs can be incorporated into classroom interventions defined in these plans.

### Are current public health surveillance mechanisms and contracts to deliver optometric eye examinations to children with learning disabilities effective?

It has been shown that the nature of the NHS England General Ophthalmic Service, which provides eligible groups with a free eye examination at an optometric practice, produces geographical inequalities regarding service provision [37] and it has been suggested that its structure and funding is a contributor to eye health inequality [38,39]. These studies evidence the inadequacy of the current General Ophthalmic Service for people with complex needs and the very low uptake of the free NHS eye examinations in our study population (10.7%) supports this conclusion.

## Limitations

The study did not explore the eye care history or status of around 20% of pupils who did not opt into the service in the schools where the service was offered. These findings cannot therefore be taken to represent the entire special school population.

## Conclusion

The very low uptake of free NHS eye examinations in our population suggests that the NHS General Ophthalmic Service is currently failing children with complex needs in England. Furthermore, only one child of 156 of school entry age would have passed routine vision screening as currently recommended by the National Screening Committee [11]. Routine vision screening is demonstrably inappropriate for this population. This and other UKstudies [2,4,5], provide evidence of a high incidence of visual deficiencies, including a high incidence of correctable refractive error, in the UK special school population. The study provides evidence that existing eye care services in England are failing to identify and manage a significant proportion of children with visual deficiencies This is evidence that children attending special schools need a targeted in-school eye care service that provides full, regular, routine, eye examinations and spectacle provision as described by the Framework for provision of eye care in special schools in England [12] and recommended by Public Health England’s screening guidelines [13].

## Supporting information

S1 Fig Parental consent form. (PDF)

S2 Fig About my child and their eyes questionnaire. (PDF)

S3 Fig Results of my child’s eye test. (PDF)

S4 Fig Location, pupil age range and school size for participating schools. (PDF)

S5 Fig Anonymised data set. (PDF)
