# Supplementary material for: Findings from an opt-in eye examination service in English special schools. Is vision screening effective for this population?
Source: PLoS One. 2019 Mar 11;14(3):e0212733. doi: 10.1371/journal.pone.0212733 (PMC6411105; doi:10.1371/journal.pone.0212733)
Supplement: S1 Fig — (PDF) [file pone.0212733.s001.pdf]

An Eye Test at xxxxx School

Dear Parent/Guardian

SeeAbility are working with your child's school to offer free specialist eye tests and glasses (when needed) in school for pupils who are not already receiving eye care. For further details please see the enclosed information sheet. SeeAbility is a national sight loss and disability charity.

We need to have your permission, so if you would like your child to have an eye test, please complete this form in BLOCK CAPITALS and return it with the '*About your child and their eyes'* questionnaire to school as soon as possible. We will then send you an appointment confirmation letter. You are very welcome to attend the appointment if you are able to. We will aim to see your child within the academic year. *Please also complete this form and explain briefly where they are being seen if you do not need SeeAbility to see your child for an eye test.*
If you have any questions please contact: us on 01372 755068 or t.omany@seeability.org

Your Child's First name:_________________ **Last name:** _________________ Male/Female

Your Child's Date of Birth: ______________**Your Child's class room:**_____________

Your home/mobile phone number: ____

Address ____ **Postcode** _________

Email address____

I **give** consent for my child to have their eyes tested Yes ☐ No ☐

I would like to attend the appointment Yes ☐ No ☐
(Appointments take place in school between 10am and 2.20pm)

*My child is already receiving eye care from:*____

If my child needs glasses:
I would like my child to choose and receive glasses at school ☐
(Where your child has not had glasses before we will also contact you before any glasses are ordered)
OR
I would like my child to be given an optical voucher to take to an opticians to choose glasses ☐

How would you prefer to be contacted by SeeAbility? Text/SMS ☐ Email ☐ Post ☐
Please tell us below if you need information providing in an alternative format

____

By signing this form I:

- Have read and understood the attached information about the eye test.
- Agree to relevant information regarding my child's eyes being shared with their school and other professionals working in school.
- Agree for a school chaperone to be present at the eye test and be party to medical information regarding my child.

Signature of parent/guardian:______________________________ Date:___________

Print name: ___________________________________ Relationship to child:___________

Note: This project complies with the 1998 Data Protection Act, which sets out rules to make sure that any information you give us is handled properly.
© SeeAbility 2017. SeeAbility is the operating name of The Royal School for the Blind founded in 1799.
Registered Charity Number 255913 No part of this document can be altered or changed without permission

Providing NHS services
