# Supplementary material for: Findings from an opt-in eye examination service in English special schools. Is vision screening effective for this population?
Source: PLoS One. 2019 Mar 11;14(3):e0212733. doi: 10.1371/journal.pone.0212733 (PMC6411105; doi:10.1371/journal.pone.0212733)
Supplement: S2 Fig — (PDF) [file pone.0212733.s002.pdf]

About your child and their eyes

easy read form

SEE ABILITY
Extraordinary every day

Please fill out as much information as possible.
Not everything in this form will be relevant to your child.

**Child's name**:	
**Date**:	

It would also be useful to see

Your child's glasses if they have any

The prescription from your child's last eye test

Your child's Health Action Plan or personal records

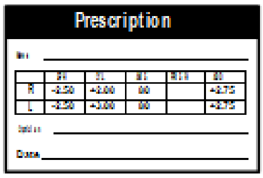

Your child's details

**Address:**	
**Phone number:**	
**Date of birth:**	
**Ethnicity:**	
**Name and address of your child's GP:**	

Information about yourself

**Name:**	
**Address:**	
**Phone number:**	
**Email address:**	
**Your relationship with the child:**	

SeeAbility is the operating name of The Royal School for the Blind founded in 1799.
Registered Charity Number 255913 © SeeAbility 2017. No part of this document can be altered or changed without permission.

Visits to the Optician

Has your child ever had an eye test at the Optician / Optometrist?

yes ☐ no ☐ don't know ☐

Which Optician / Optometrist?	
Date of last check:	
Date of next check:	

Was your child prescribed glasses?

yes ☐ no ☐ don't know ☐

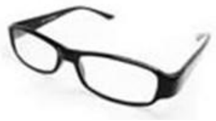

Visits to the Hospital

Has your child ever been to see the eye clinic in a hospital?

yes ☐ no ☐ don't know ☐

If yes, what was the problem?

Which hospital did they go to?	
Date of last appointment:	
Date of next appointment:	

Was your child prescribed glasses?

yes ☐ no ☐ don't know ☐

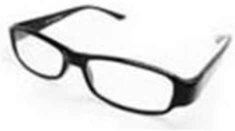

Is your child using their glasses now?

yes ☐ no ☐ don't know ☐

Was your child given patching?

yes ☐ no ☐ don't know ☐

Did your child have an operation on their eyes?

yes ☐ no ☐ don't know ☐

SeeAbility is the operating name of The Royal School for the Blind founded in 1799.
Registered Charity Number 255913 © SeeAbility 2017. No part of this document can be altered or changed without permission.

Is your child registered blind / severely sight impaired?

yes ☐ no ☐ don't know ☐

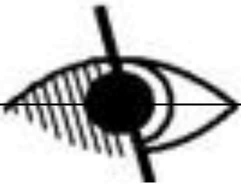

Is your child registered partially blind / sight impaired?

yes ☐ no ☐ don't know ☐

Is your child using their glasses now?

yes ☐ no ☐ don't know ☐

Does your child have any problems with their glasses?

yes ☐ no ☐ don't know ☐

Comments:

Do you have any concerns about your child's eyes?

yes ☐ no ☐ don't know ☐

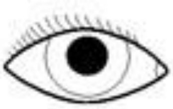

If yes, please write what the problems are:

Is there a history of blastoma in your family?

yes ☐ no ☐ don't know ☐

Other useful information

Has anyone in your family had eye problems?

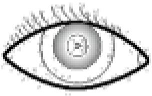

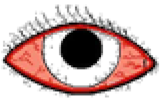

yes ☐ no ☐ don't know ☐

For example has anyone needed to wear glasses as a child, had a squint / strabismus, patching one eye or an eye condition or blastoma?

If yes, please write who has had problems and what problems they've had:

**Who**	**What problem**

SeeAbility is the operating name of The Royal School for the Blind founded in 1799.
Registered Charity Number 255913 © SeeAbility 2017. No part of this document can be altered or changed without permission.

Do your child’s eyes always appear straight?

yes ☐ no ☐ don’t know ☐

If one/both eyes appear to wander, which eye does this and how often? Do your child’s eyes ever appear to move excessively rapidly or uncontrollably? Do you think your child has trouble controlling his/her eye movements? *If so please give details if possible:*

Does your child complain of headaches?

yes ☐ no ☐ don’t know ☐

Does your child tend to shut one eye?

yes ☐ no ☐ don’t know ☐

Does your child appear very sensitive to bright lights?

yes ☐ no ☐ don’t know ☐

More about your child

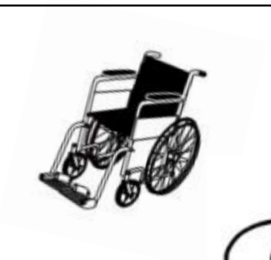

Does your child use a wheelchair?

yes ☐ no ☐ sometimes ☐

Does your child have any health problems or disabilities?

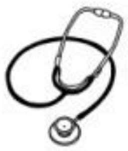

yes ☐ no ☐ sometimes ☐

If yes, please say what they are:

SeeAbility is the operating name of The Royal School for the Blind founded in 1799.
Registered Charity Number 255913 © SeeAbility 2017. No part of this document can be altered or changed without permission.

Birth history

Please give details of any difficulties with your pregnancy or child's birth.
(i.e: mother having infection, prematurity, low birth weight, need for special care etc)

Does your child take any medication?

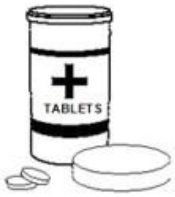

yes ☐ no ☐ don't know ☐

If yes, please take information about your child's medication with you to the eye test and list them below:

Does your child have any allergies?

yes ☐ no ☐ don't know ☐

If yes, please list them below:

Is your child deaf or hard of hearing?

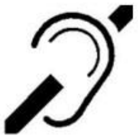

yes ☐ no ☐

If yes, please tell the optometrist about your child's hearing:

Does your child find it hard to communicate?

yes ☐ no ☐ sometimes ☐

SeeAbility is the operating name of The Royal School for the Blind founded in 1799.
Registered Charity Number 255913 © SeeAbility 2017. No part of this document can be altered or changed without permission.

What helps your child to communicate? Tell us if you use things like:

Makaton *yes* ☐ *no* ☐

An interpreter yes ☐ no ☐

Pictures yes ☐ no ☐

Gestures yes ☐ no ☐

Other *(please describe how you communicate with your child):*

About your child's eyes

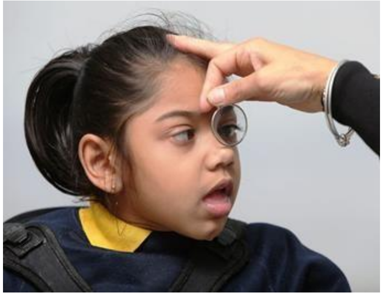

When your child has their eye test the optometrist will need to look at their eyes. They will also do some tests to check how well they can see.

The information you give the optometrist will help them to test your child's eyes.

Can your child say or sign the names of letters on any eye test chart?

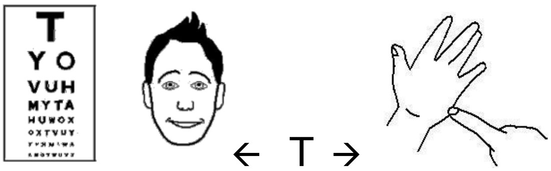

yes ☐ no ☐ don't know ☐

Can your child say or sign the names of pictures on a chart like **house**, **fish** or **car**?

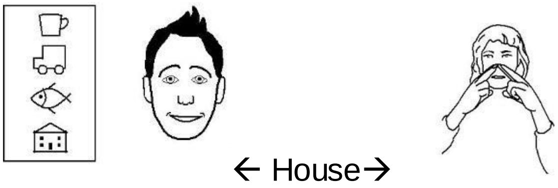

yes ☐ no ☐ don't know ☐

Can your child point to a letter or picture on a card that is the same as a letter or picture on a chart on the wall?

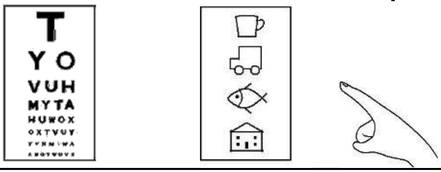

yes ☐ no ☐ don't know ☐

Will your child be able to wear test frames on their face?

yes ☐ no ☐ don't know ☐

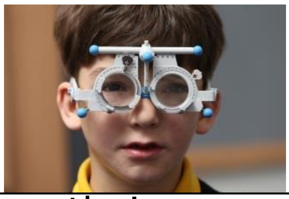

Will your child be okay if the optometrist were to cover their eyes one at a time?

SeeAbility is the operating name of The Royal School for the Blind founded in 1799.
Registered Charity Number 255913 © SeeAbility 2017. No part of this document can be altered or changed without permission.

yes ☐ no ☐ don't know ☐

Can your child understand '**better**' and '**worse**'?

yes ☐ no ☐ don't know ☐

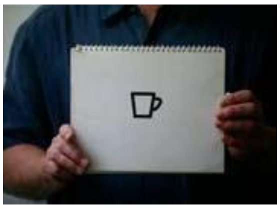

better

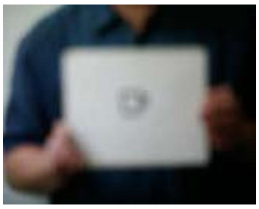

worse

Will your child be okay if the optometrist comes close to them and shines a bright light in their eye?

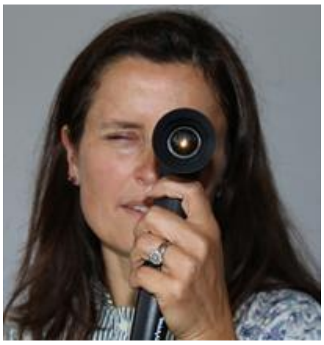

yes ☐ no ☐ don't know ☐

Will your child be okay if the optometrist puts drops in their eyes? Some eye drops may sting for a bit, or make it hard to see for a short time. You can ask the optometrist about this.

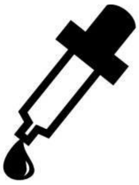

yes ☐ no ☐ don't know ☐

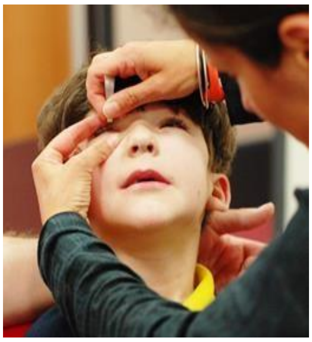

Five key questions

We would also like you to consider the following five questions. These questions are designed for a range of ages and abilities, so some questions may not be relevant. Your child may have difficulty with some behaviours listed below but not others – this is normal. You may also notice that some of the behaviours described occur only occasionally or more frequently when your child is tired, this is common.

Tick the box which best describes your child's current behaviour and if for example your child uses a wheelchair and the stairs question is not relevant, ring "not applicable".

SeeAbility is the operating name of The Royal School for the Blind founded in 1799.
Registered Charity Number 255913 © SeeAbility 2017. No part of this document can be altered or changed without permission.

1. **Does your child have difficulty walking downstairs?**

Never	Rarely	Sometimes	Often	Always	Not applicable

2. **Does your child have difficulty seeing fast-moving objects?**

Never	Rarely	Sometimes	Often	Always	Not applicable

3. **Does your child have difficulty seeing something that is pointed out in the distance?**

Never	Rarely	Sometimes	Often	Always	Not applicable

4. **Does your child have difficulty locating an item of clothing in a pile of clothes?**

Never	Rarely	Sometimes	Often	Always	Not applicable

5. **Does your child find copying words or pictures time-consuming and difficult?**

Never	Rarely	Sometimes	Often	Always	Not applicable

Please tell us any other information we may need to know:

www.seeability.org

Follow us on **Facebook** and **Twitter**

Reviewed: November 2017

SeeAbility is the operating name of The Royal School for the Blind founded in 1799.
Registered Charity Number 255913 © SeeAbility 2017. No part of this document can be altered or changed without permission.
