# Supplementary material for: Findings from an opt-in eye examination service in English special schools. Is vision screening effective for this population?
Source: PLoS One. 2019 Mar 11;14(3):e0212733. doi: 10.1371/journal.pone.0212733 (PMC6411105; doi:10.1371/journal.pone.0212733)
Supplement: S3 Fig — (PDF) [file pone.0212733.s003.pdf]

SEE ABILITY
Extraordinary every day

The results of your child's eye test

The information in this form will help everyone to understand your child's eye sight, eye health and their visual strengths and limitations. This form includes your child's glasses prescription, a copy should be kept with your child's health records and support plan in school.

**Section 1 – Details of child**		
**Child's name and date of birth:**		

**Section 2 – Dates of the eye test**	
**Date of this test:**	
**Recommended date of next test:**	

**Section 3 – Additional detail about the eye test**	
**Where did the test take place and who was present?**	
**What was already known about eyes and vision?**<br>**Did anyone have questions about eyes and vision?**	

**Section 4 – Assessors**	
**Whom is this report from?**<br>**Name:**<br>**Role:**<br>**Address: SeeAbility, New Plan House, 41 East Street, Epsom, KT17 1BL**	
**Who is getting a copy of this report?**	**Parents and school**

SeeAbility is the operating name of The Royal School for the Blind founded in 1799. Registered Charity Number 255913 © SeeAbility 2017.Developed in partnership with Ulster University. No part of this document can be altered or changed without permission.

Section 5 – Summary

ABOUT THE CHILD'S EYES AND VISION:

ACTIONS FROM TODAY'S TEST:

Glasses needed

Modifications to classroom / schoolwork needed

Education Health and Care Plan should include information about vision needs

Child is eligible for certification as visually

impaired If yes, is certification in place?

GP action required

Another specialist needs to see this child

SeeAbility is the operating name of The Royal School for the Blind founded in 1799. Registered Charity Number 255913 © SeeAbility 2017.Developed in partnership with Ulster University. No part of this document can be altered or changed without permission.

Section 6 – We tested to see if glasses are needed

We were able to test for glasses today

Glasses are needed

Why are glasses needed?

We measured for focusing accuracy:

We gave a prescription for glasses:

RIGHT		Sph	Cyl	Axis	Prism	Sph	Cyl	Axis	Prism	LEFT
	Dist									
	Near									

What are these glasses for?

Please see SeeAbility website for more information about Wearing Glasses: www.seeability.org/childreninfocus

SeeAbility is the operating name of The Royal School for the Blind founded in 1799. Registered Charity Number 255913 © SeeAbility 2017.Developed in partnership with Ulster University. No part of this document can be altered or changed without permission.

Section 7 – Results of the vision tests we did today

Visual acuity describes how well a person sees black on white detail with glasses if needed.

The vision results were:

Visual acuity for looking at things in the distance:

This was tested today:

Visual acuity for looking at things close up:

This was tested today:

We have included examples that this child should easily see:

Binocular vision and eye movements:
This is how well your child's eyes work together

This was tested today:

SeeAbility is the operating name of The Royal School for the Blind founded in 1799. Registered Charity Number 255913 © SeeAbility 2017.Developed in partnership with Ulster University. No part of this document can be altered or changed without permission.

Visual field:
This is how well your child can see things to the side of their central vision

This was tested today:

.

Contrast sensitivity:
This is how well objects are seen against different backgrounds

This was tested today:

Cerebral Visual Impairment (CVI):
This is when there are visual difficulties caused by problems in the brain rather than the eyes

Signs of CVI?

Section 8 – Results of the eye health check

The health of the eyes – inside and out:

This was checked today:

The child needs to see another specialist about their eye health

.

SeeAbility is the operating name of The Royal School for the Blind founded in 1799. Registered Charity Number 255913 © SeeAbility 2017.Developed in partnership with Ulster University. No part of this document can be altered or changed without permission.

Section 9 – Technical details for other health professionals

Visual acuity :

Refractive error :

Accommodative function (dynamic ret):

Ocular posture and eye movement assessment:

Contrast sensitivity :

Visual field:

CVI (key questions):

Other tests:

Eye health exam:

SeeAbility is the operating name of The Royal School for the Blind founded in 1799. Registered Charity Number 255913 © SeeAbility 2017.Developed in partnership with Ulster University. No part of this document can be altered or changed without permission.
