# Supplementary material for: Findings from an opt-in eye examination service in English special schools. Is vision screening effective for this population?
Source: PLoS One. 2019 Mar 11;14(3):e0212733. doi: 10.1371/journal.pone.0212733 (PMC6411105; doi:10.1371/journal.pone.0212733)
Supplement: S4 Fig — (PDF) [file pone.0212733.s004.pdf]

Supplementary Fig 4. School locations, age ranges and pupil numbers

School/ Academy	Location	Pupil Age Range	Total on School Roll at July 2017	Number seen by service at July 2017
The Village School	London Borough of Brent, NW9	2-19	275	240 (87%)
Moorcroft School	Eden Academy, Uxbridge London Borough of Hillingdon UB8	11-19	71	57 (80%)
Grangewood School	Eden Academy, Pinner, London Borough of Harrow, HA5	3-11	107	71 (66%)
Perseid Upper School	London Borough of Merton SM4	11-19	51	47 (92%)
Perseid Lower School	London Borough of Merton, SM4	3-11	81	63 (78%)
Willowdene School	London Borough of Greenwich SE18	2-11	198	148 (75%)
Oakmere School	London Borough of Greenwich SE18	11-16	30	20 (67%)
Charlton Park Academy	London Borough of Greenwich SE18	11-19	197	144 (73%)
Heritage House School	Chesham, Buckinghamshire HP5	2-19	59	39 (66%)
Trinity School and Sport College- *service not offered to full school roll*	Durham DH1	2-19 (4-7 only offered service)	213	56 (26%)
Newbridge Academy - *service not offered to full school roll*	Oldham, Manchester OL8	11-19 *(11-13 only offered service at July 2017)	346	38 (11%)

*we only began inviting this school in April 2017
