# Supplementary material for: Findings from an opt-in eye examination service in English special schools. Is vision screening effective for this population?
Source: PLoS One. 2019 Mar 11;14(3):e0212733. doi: 10.1371/journal.pone.0212733 (PMC6411105; doi:10.1371/journal.pone.0212733)
Supplement: S5 Fig — (PDF) [file pone.0212733.s005.pdf]

Year of eye exam	Pupil code	Age (years)	School age	UK screening age	Eye care history	Presenting VA LogMAR	Low vision
1	M001	13.17	Secondary	no	no eye test	0	no
1	M002	5.58	Primary	yes	HES discharged	0.2	no
1	M003	12.42	Secondary	no	HES discharged	0.4	no
1	M004	18.58	Secondary	no		0.1	no
1	M005	19.17	Secondary	no	HES discharged	0.3	no
1	M006	18.42	Secondary	no	no eye test	0.5	yes
1	M007	17.5	Secondary	no	HES discharged		
1	M008	17.17	Secondary	no	no eye test	0	no
1	M009	18.42	Secondary	no	HES discharged		
1	M010	17.83	Secondary	no	current HES	0.2	no
1	M011	18.33	Secondary	no	HES discharged	-0.04	no
1	M012	19.58	Secondary	no		0.2	no
1	M013	17.67	Secondary	no	no eye test	0.5	yes
1	M014	17.67	Secondary	no	no eye test	0.7	yes
1	M015	15.5	Secondary	no	no eye test		
1	M016	13.25	Secondary	no	no eye test	0.1	no
1	M017	14.33	Secondary	no	no eye test		
1	M018	14.33	Secondary	no	no eye test		
1	M019	12.17	Secondary	no	HES discharged		
1	M020	17	Secondary	no	current HES		
1	M021	17.58	Secondary	no	HES discharged		
1	M022	14.33	Secondary	no	optom only		
1	M023	15.5	Secondary	no	HES discharged		
1	M024	15.17	Secondary	no	HES discharged		
1	M025	18	Secondary	no	HES discharged		
1	M026	16.67	Secondary	no	HES discharged		
1	M027	16.25	Secondary	no	HES discharged	0.6	yes
1	M028	11.83	Secondary	no	no eye test		
1	PL001	4.5	Primary	yes	current HES		
1	PL002	6.25	Primary	no	current HES		
1	PL003	6.5	Primary	no	HES discharged		
1	PL004	7.83	Primary	no	HES discharged		
1	PL005	5	Primary	yes			
1	PL006	3.92	Primary	no	HES discharged		
1	PL007	10.33	Primary	no	optom only		
1	PL008	10.67	Primary	no	current HES		
1	PL009	10.33	Primary	no	current HES		
1	PL010	7.5	Primary	no			
1	PL011	8.17	Primary	no	current HES		
1	PL012	8.42	Primary	no	optom only	0	no
1	PL013	8.5	Primary	no	no eye test	0.4	no
1	PL014	7.67	Primary	no	current HES		
1	PL015	5.25	Primary	yes	current HES		
1	PL016	5.5	Primary	yes	HES discharged	0.2	no
1	PL017	7.33	Primary	no	no eye test		
1	PL018	7.33	Primary	no	no eye test	0.3	no

1	PL019	4.17	Primary	yes	no eye test	0.3	no
1	PL020	4.08	Primary	yes	current HES	0.7	yes
1	PL021	5.25	Primary	yes	no eye test	0.8	yes
1	PL022	5.17	Primary	yes		0	no
1	PL023	9.92	Primary	no	HES discharged	0	no
1	PL024	5.17	Primary	yes	HES discharged	0.4	no
1	PL025	4	Primary	yes			
1	PL026	4.08	Primary	yes	current HES	0.7	yes
1	PL027	10.33	Primary	no	current HES	0.2	no
1	PL028	6.25	Primary	no	no eye test	0.7	yes
1	PL031	7.75	Primary	no	current HES	0.8	yes
1	PL032	6.42	Primary	no	current HES	0	no
1	PL033	6.67	Primary	no	current HES	0.4	no
1	PL034	6.83	Primary	no	current HES	0.3	no
1	PL035	6.67	Primary	no	HES discharged	0.4	no
1	PL036	5.33	Primary	yes	no eye test	0.9	yes
1	PU001	13.67	Secondary	no	no eye test		
1	PU002	11.75	Secondary	no	no eye test	0.4	no
1	PU003	12.33	Secondary	no	no eye test		
1	PU004	12.5	Secondary	no	current HES	0	no
1	PU005	12.17	Secondary	no	HES discharged	0.1	no
1	PU006	12.25	Secondary	no	optom only		
1	PU007	12	Secondary	no	HES discharged		
1	PU008	12.75	Secondary	no	HES discharged		
1	PU009	13.58	Secondary	no	current HES		
1	PU010	13.5	Secondary	no	HES discharged	0.5	yes
1	PU011	13.33	Secondary	no	HES discharged	0.7	yes
1	PU012	12.08	Secondary	no	current HES		
1	PU013	15.25	Secondary	no	HES discharged	0.13	no
1	PU014	17.92	Secondary	no	no eye test		
1	PU015	15.92	Secondary	no	HES discharged		
1	PU016	15.25	Secondary	no	HES discharged	0.4	no
1	PU017	16.75	Secondary	no	current HES	0.3	no
1	PU018	15.33	Secondary	no	no eye test		
1	PU019	16.5	Secondary	no	HES discharged	0	no
1	PU020	17.5	Secondary	no	no eye test	0.1	no
1	PU021	17.75	Secondary	no	no eye test	0.1	no
1	PU022	17.83	Secondary	no	no eye test	0.15	no
1	PU023	15.75	Secondary	no	no eye test		
1	PU024	16.08	Secondary	no	current HES		
1	PU025	13.75	Secondary	no	no eye test		
1	PU026	16	Secondary	no	current HES	0	no
1	VS001	14.08	Secondary	no	optom only	1	yes
1	VS002	14.92	Secondary	no	current HES	0.8	yes
1	VS003	11.75	Secondary	no	current HES	0.4	no
1	VS004	14.5	Secondary	no	current HES	0.4	no
1	VS005	6.75	Primary	no	no eye test	0.6	yes
1	VS006	11.42	Secondary	no	HES discharged	0.1	no
1	VS007	6.83	Primary	no	no eye test		
1	VS008	7.92	Primary	no	no eye test	0.2	no

1	VS009	6.75	Primary	no	no eye test	0.5	yes
1	VS010	6.92	Primary	no	no eye test	0.6	yes
1	VS011	7.08	Primary	no	no eye test		
1	VS012	5.92	Primary	yes	HES discharged		
1	VS013	5	Primary	yes	current HES	0.5	yes
1	VS014	6.58	Primary	no	current HES	0.5	yes
1	VS015	6.17	Primary	no	current HES	0.5	yes
1	VS016	6.67	Primary	no	current HES		
1	VS017	14.67	Secondary	no	no eye test	0.2	no
1	VS018	16.17	Secondary	no		0.2	no
1	VS019	7.5	Primary	no	current HES	0.6	yes
1	VS020	5.42	Primary	yes	no eye test	0.15	no
1	VS021	8.42	Primary	no	HES discharged		
1	VS022	8.42	Primary	no	no eye test		
1	VS023	7.5	Primary	no	current HES	0.3	no
1	VS024	7.25	Primary	no	current HES	0.9	yes
1	VS025	11.08	Secondary	no	no eye test	0.3	no
1	VS026	6.33	Primary	no	current HES		
1	VS027	10.42	Primary	no	current HES		
1	VS028	18.75	Secondary	no		0.7	yes
1	VS029	16.25	Secondary	no	no eye test	0.2	no
1	VS030	6.58	Primary	no	no eye test	0.3	no
1	VS031	10.42	Primary	no	no eye test		
1	VS032	7.08	Primary	no	HES discharged		
1	VS033	8.33	Primary	no	no eye test	0.2	no
1	VS034	8.42	Primary	no	current HES	0.4	no
1	VS035	9.75	Primary	no		-0.1	no
1	VS036	12.17	Secondary	no	optom only	0.3	no
1	VS037	13.83	Secondary	no	no eye test	0	no
1	VS038	12.25	Secondary	no	no eye test	0	no
1	VS039	12.25	Secondary	no	no eye test	-0.1	no
1	VS040	8.67	Primary	no	no eye test		
1	VS041	8.25	Primary	no	no eye test	0	no
1	VS042	13.67	Secondary	no	HES discharged		yes
1	VS043	11.17	Secondary	no	HES discharged	0.3	no
1	VS044	9.25	Primary	no	no eye test	0.5	yes
1	VS045	11.17	Secondary	no	no eye test	0	no
1	VS046	7.33	Primary	no	HES discharged		
1	VS047	9.83	Primary	no	HES discharged	0.5	yes
1	VS048	13.08	Secondary	no			
1	VS049	17.17	Secondary	no	no eye test	0.2	no
1	VS050	13.92	Secondary	no		0	no
1	VS051	10.75	Primary	no	optom only	0.35	no
1	VS052	19.25	Secondary	no		0.2	no
1	VS053	15.08	Secondary	no	optom only	0.28	no
1	VS054	12.58	Secondary	no	no eye test	-0.1	no
1	VS055	9.58	Primary	no		0.05	no
1	VS055	7.08	Primary	no	no eye test		
1	VS057	10.42	Primary	no	current HES	0.85	yes
1	VS058	16.75	Secondary	no	current HES	0.3	no

1	VS059	12.25	Secondary	no	current HES	0	no
1	VS060	16.5	Secondary	no	current HES	0	no
1	VS061	19.08	Secondary	no		0.2	no
1	VS062	15.42	Secondary	no	HES discharged	0.8	yes
1	VS063	7.83	Primary	no	no eye test		
1	VS064	6.42	Primary	no			
1	VS065	5.67	Primary	yes	HES discharged		
1	VS066	7.33	Primary	no	optom only	0.16	no
1	VS067	9.42	Primary	no	current HES	0.18	no
1	VS068	13.5	Secondary	no	HES discharged		
1	VS069	14.92	Secondary	no	HES discharged	0.5	yes
1	VS070	14.08	Secondary	no	no eye test	0.5	yes
1	VS071	14.75	Secondary	no	HES discharged	0.8	yes
1	VS072	6.75	Primary	no	HES discharged	0.4	no
1	VS073	5.17	Primary	yes	no eye test		
1	VS074	7.58	Primary	no	HES discharged	0.2	no
1	VS075	5.08	Primary	yes	HES discharged		yes
1	VS076	16.17	Secondary	no	HES discharged	0.35	no
1	VS077	14.92	Secondary	no	optom only	0	no
1	VS078	14.25	Secondary	no		0	no
1	VS079	16.5	Secondary	no		0.4	no
1	VS080	14.83	Secondary	no	current HES	0.6	yes
1	VS081	19.33	Secondary	no	no eye test		
1	VS082	6.42	Primary	no	current HES		
1	VS083	18.08	Secondary	no			
1	VS084	8.67	Primary	no	current HES		
1	VS085	6.5	Primary	no	current HES		yes
1	VS086	8	Primary	no	no eye test	0.5	yes
1	VS087	4.83	Primary	yes	HES discharged		
1	VS088	8	Primary	no	no eye test	0	no
1	VS089	19.42	Secondary	no	no eye test	0.13	no
1	VS090	8.33	Primary	no	no eye test	0	no
1	VS091	7.83	Primary	no	current HES	0.4	no
1	VS092	10.33	Primary	no	HES discharged	0	no
1	VS093	9.58	Primary	no	HES discharged		
1	VS094	10.83	Primary	no	no eye test	0.3	no
1	VS095	5.17	Primary	yes	current HES	0.7	yes
1	VS096	17.67	Secondary	no	no eye test	0.3	no
1	VS097	17.33	Secondary	no	optom only	0.4	no
1	VS098	16.17	Secondary	no	no eye test	0	no
1	VS099	16.58	Secondary	no	current HES	0	no
1	VS100	18.25	Secondary	no		0.5	yes
1	VS101	9.08	Primary	no	no eye test	-0.1	no
1	VS102	10.67	Primary	no	optom only	-0.1	no
1	VS103	19	Secondary	no	no eye test	0	no
1	VS104	12.08	Secondary	no	no eye test	0.05	no
1	VS105	12.67	Secondary	no	current HES		yes
1	VS106	18.08	Secondary	no	HES discharged	0.25	no
1	VS107	12.17	Secondary	no		0	no
1	VS108	13.58	Secondary	no		0.2	no

1	VS109	12.33	Secondary	no	HES discharged	0.2	no
1	VS110	12.25	Secondary	no		0.1	no
1	VS111	18.92	Secondary	no	HES discharged	0.15	no
1	VS112	17.75	Secondary	no	current HES	0.23	no
1	VS113	16.33	Secondary	no	no eye test	0.2	no
1	VS114	15.58	Secondary	no	optom only	0	no
1	VS115	19.58	Secondary	no	no eye test	-0.03	no
1	VS116	19.42	Secondary	no	HES discharged	0.55	yes
1	VS117	14.5	Secondary	no	current HES	0.3	no
1	VS118	14.5	Secondary	no	no eye test		yes
1	VS119	12.75	Secondary	no	current HES	0.3	no
1	VS120	18	Secondary	no	optom only	0.4	no
1	VS121	16.67	Secondary	no	optom only	0	no
1	VS122	14.58	Secondary	no	HES discharged		
1	VS123	19.17	Secondary	no	HES discharged	0.03	no
1	VS124	12.67	Secondary	no	current HES	0.45	no
1	VS125	19.25	Secondary	no		0.4	no
1	VS126	15.83	Secondary	no	no eye test	0	no
1	VS127	19.58	Secondary	no	no eye test	0.1	no
1	VS128	16.92	Secondary	no	no eye test		
1	VS129	19.5	Secondary	no	current HES		yes
1	VS130	19.25	Secondary	no	HES discharged	0.2	no
1	VS131	14.5	Secondary	no	current HES		yes
1	VS132	19.58	Secondary	no	no eye test	-0.1	no
2	GW002	6	Primary	no	HES discharged		
2	GW003	7	Primary	no	no eye test		
2	GW004	5.5	Primary	yes	no eye test		
2	GW005	7	Primary	no		0.6	yes
2	GW006	6.5	Primary	no	no eye test		
2	GW007	7	Primary	no	HES + optom		
2	GW008	7.5	Primary	no	no eye test	0.4	no
2	GW009	7	Primary	no			
2	GW010	6	Primary	no	current HES	0.5	yes
2	GW011	4	Primary	yes	HES discharged	0.4	no
2	GW012	5	Primary	yes	no eye test		
2	GW013	7	Primary	no	HES discharged		
2	GW014	6.5	Primary	no	no eye test	0.4	no
2	GW015	5	Primary	yes			
2	GW016	8	Primary	no		0.4	no
2	GW017	6	Primary	no			
2	M034	17	Secondary	no			
2	M034	19	Secondary	no			
2	M035	15.5	Secondary	no	current HES	0.3	no
2	M036	19.5	Secondary	no	HES discharged		
2	M037	12.5	Secondary	no	HES discharged	0	no
2	PL037	5	Primary	yes	no eye test		
2	PL038	7	Primary	no	no eye test	0.6	yes
2	PL039	7.5	Primary	no	HES discharged	0.1	no
2	PL040	4.5	Primary	yes	no eye test	0.2	no
2	PL041	4	Primary	yes			

2	PL042	5.5	Primary	yes			
2	PL043	4	Primary	yes		0.6	yes
2	PL044	4.5	Primary	yes			
2	PL045	7	Primary	no	current HES		
2	PL046	5.5	Primary	yes	current HES	0.3	no
2	PU028	19	Secondary	no	no eye test	0	no
2	VS001	12	Secondary	no	no eye test		
2	VS133	4	Primary	yes	current HES	0.8	yes
2	VS134	4.5	Primary	yes	current HES		
2	VS135	4.5	Primary	yes	HES discharged	0.8	yes
2	VS136	6	Primary	no	current HES	0.3	no
2	VS137	5	Primary	yes	current HES	0.8	yes
2	VS138	4	Primary	yes	current HES		yes
2	VS139	5	Primary	yes	HES discharged	0.5	yes
2	VS140	5	Primary	yes	HES discharged		
2	VS141	5	Primary	yes	HES discharged	0.5	yes
2	VS142	5	Primary	yes	current HES		
2	VS143	14	Secondary	no		0	no
2	VS144	4	Primary	yes	no eye test		
2	VS145	7	Primary	no	current HES		
2	VS146	4.5	Primary	yes	no eye test	0.3	no
2	VS147	4.5	Primary	yes	current HES		
2	VS148	8.5	Primary	no	no eye test	0.4	no
2	VS149	4.5	Primary	yes	current HES	0.6	yes
2	VS150	9	Primary	no	current HES	0.3	no
2	VS151	5	Primary	yes	HES discharged	0.6	yes
2	VS152	18	Secondary	no	current HES		
2	VS153	13.5	Secondary	no	current HES	0.3	no
2	VS154	6	Primary	no	current HES		
2	VS155	14	Secondary	no	HES discharged	0.1	no
2	VS156	10	Primary	no	no eye test	0.4	no
2	VS157	16	Secondary	no	HES discharged	0.3	no
2	VS158	8.5	Primary	no	HES discharged	0.38	no
2	VS159	12	Secondary	no	current HES	0.9	yes
2	VS160	11.5	Secondary	no	HES discharged		
2	VS161	5	Primary	yes	current HES	1	yes
2	VS162	5.5	Primary	yes	no eye test		
2	VS163	11.5	Secondary	no	no eye test	0.2	no
2	VS164	14.5	Secondary	no	current HES		
2	VS165	15	Secondary	no	HES + optom	0.15	no
2	VS166	14	Secondary	no			yes
2	VS167	8.5	Primary	no	current HES	0.23	no
2	VS168	16	Secondary	no	no eye test		
2	VS169	13.5	Secondary	no	optom only	0.2	no
2	VS170	10.5	Primary	no	HES discharged		
2	VS171	16	Secondary	no	HES + optom	0.53	yes
2	VS172	9.5	Primary	no	current HES	0.25	no
2	VS173	9.5	Primary	no	no eye test	0.35	no
2	VS174	14.5	Secondary	no	current HES	1	yes
2	VS175	9	Primary	no	current HES		

2	VS176	7	Primary	no	current HES	0.2	no
2	VS177	6.5	Primary	no	current HES	0.15	no
2	VS178	13	Secondary	no	no eye test	0.3	no
2	VS179	13.5	Secondary	no	no eye test	0.1	no
2	VS180	16	Secondary	no	HES discharged	0.6	yes
2	VS181	14	Secondary	no			
2	VS182	4.5	Primary	yes	no eye test		
2	VS183	15	Secondary	no	HES discharged	0.1	no
2	VS185	12.5	Secondary	no	optom only	0.03	no
2	VS186	16.5	Secondary	no	optom only	0	no
2	VS187	13	Secondary	no	current HES	0	no
2	VS188	14.5	Secondary	no		0.5	yes
2	VS189	10.5	Primary	no	optom only	0	no
2	VS190	14	Secondary	no		0.13	no
2	VS191	8.5	Primary	no	current HES	0.35	no
2	VS192	10.5	Primary	no	no eye test	0.5	yes
2	VS193	15.5	Secondary	no		0.4	no
2	VS194	11	Secondary	no	current HES	0.4	no
2	VS195	8	Primary	no	current HES	0.7	yes
2	VS196	9	Primary	no	current HES	0.2	no
2	VS197	18.5	Secondary	no	HES discharged	0.5	yes
2	VS198	9	Primary	no	current HES	0.8	yes
2	VS199	7	Primary	no	no eye test		
2	VS200	7	Primary	no	no eye test	0.2	no
2	VS201	6	Primary	no		0.08	no
2	VS202	7	Primary	no			
2	VS203	17.5	Secondary	no	no eye test	0.45	no
2	VS204	9	Primary	no	no eye test	0.1	no
2	VS205	9	Primary	no	no eye test	-0.15	no
2	VS206	5	Primary	yes	current HES	0.4	no
2	VS207	14.5	Secondary	no	optom only		
2	VS208	8	Primary	no	optom only	0	no
2	VS209	6.5	Primary	no	optom only		
2	VS210	14.5	Secondary	no	current HES	0.7	yes
2	VS211	12	Secondary	no		0.43	no
2	VS212	6	Primary	no	HES discharged		
2	VS213	6.5	Primary	no	no eye test	0.2	no
2	VS214	18	Secondary	no	HES discharged	0.33	no
2	VS215	14	Secondary	no	no eye test	0.2	no
2	VS216	16	Secondary	no		0.6	yes
2	VS217	8	Primary	no	current HES		yes
2	VS218	10	Primary	no		0.7	yes
2	W001	8	Primary	no	current HES		yes
2	W002	6.5	Primary	no	no eye test	0.25	no
2	W003	9.5	Primary	no	optom only	0.4	no
2	W004	9	Primary	no	no eye test	0.03	no
2	W005	7	Primary	no	current HES	0	no
2	W006	6.5	Primary	no	no eye test		
2	W007	8	Primary	no	current HES		yes
2	W008	10.5	Primary	no	HES + optom		

2	W009	5.5	Primary	yes	no eye test	0.4	no
2	W010	8	Primary	no	no eye test	0.3	no
2	W011	8	Primary	no	no eye test	0.2	no
2	W012	10.5	Primary	no	optom only		
2	W013	10	Primary	no	HES + optom	0	no
2	W014	6.5	Primary	no	HES discharged		
2	W015	5	Primary	yes	HES discharged		
2	W016	6	Primary	no	no eye test		
2	W017	5	Primary	yes	no eye test		
2	W018	5.5	Primary	yes	no eye test	0.8	yes
2	W019	5.5	Primary	yes	HES discharged		
2	W020	5	Primary	yes	no eye test		
2	W021	6.5	Primary	no		0.25	no
2	W022	5.5	Primary	yes			
2	W023	7	Primary	no	HES discharged		
2	W024	5	Primary	yes		0.8	yes
2	W025	4.5	Primary	yes	current HES		
2	W026	6.5	Primary	no	no eye test	0.2	no
2	W027	6.5	Primary	no	no eye test	0.5	yes
2	W028	7	Primary	no	no eye test		
2	W029	6	Primary	no	no eye test		
2	W030	7	Primary	no	no eye test	0.1	no
2	W031	7	Primary	no	HES discharged	0	no
2	W032	7	Primary	no	HES discharged	0.14	no
2	W033	6	Primary	no	no eye test		
2	W034	5	Primary	yes	HES discharged		yes
2	W035	7.5	Primary	no	no eye test	0	no
2	W036	6	Primary	no	no eye test	0.6	yes
2	W037	7.5	Primary	no	no eye test		
2	W038	7.5	Primary	no	no eye test	0.6	yes
2	W039	9	Primary	no		0.2	no
2	W040	11	Secondary	no	no eye test		
2	W041	7.5	Primary	no	no eye test	0.4	no
2	W042	10.5	Primary	no	no eye test	0.5	yes
2	W043	8	Primary	no			
2	W044	7	Primary	no	no eye test	0.13	no
2	W045	7	Primary	no	no eye test	0.1	no
2	W046	5.5	Primary	yes			
2	W047	5.5	Primary	yes	HES discharged		
2	W048	12	Secondary	no	no eye test	0.7	yes
2	W049	7	Primary	no	no eye test	0.4	no
2	W050	8.5	Primary	no	no eye test		
2	W051	8	Primary	no	optom only		
2	W052	10	Primary	no	optom only		yes
2	W053	8.5	Primary	no			yes
2	W054	8.5	Primary	no	no eye test	0.5	yes
2	W055	6	Primary	no	no eye test		
2	W056	9.5	Primary	no	no eye test	0.2	no
2	W057	10.5	Primary	no			yes
2	W058	7.5	Primary	no		0.1	no

2	W059	9	Primary	no	no eye test	0	no
2	W060	7	Primary	no			
2	W061	7.5	Primary	no	optom only	0	no
2	W062	12	Secondary	no	no eye test		yes
3	CP01	14.83	Secondary	no	seen at school		
3	CP02	17.5	Secondary	no	no eye test		
3	CP03	18.42	Secondary	no	no eye test	-0.1	no
3	CP04	17.33	Secondary	no	HES discharged	0.4	no
3	CP05	17.08	Secondary	no	no eye test	0.5	yes
3	CP06	15.67	Secondary	no	HES discharged	0	no
3	CP07	18	Secondary	no	current HES		yes
3	CP08	15.83	Secondary	no	HES discharged		yes
3	CP09	17.92	Secondary	no	HES discharged		yes
3	CP10	13.75	Secondary	no	HES discharged		yes
3	CP11	16	Secondary	no	HES discharged		yes
3	CP12	13.33	Secondary	no	current HES	0.2	no
3	CP13	12.17	Secondary	no	HES discharged	0	no
3	CP14	11.83	Secondary	no	optom only	0.1	no
3	CP15	11.67	Secondary	no	no eye test	0.1	no
3	CP16	18.67	Secondary	no	HES discharged	0.1	no
3	CP17	12.33	Secondary	no	no eye test		
3	CP18	17	Secondary	no	current HES	0.43	no
3	CP19	13.58	Secondary	no	no eye test	0.03	no
3	CP20	16.5	Secondary	no	no eye test	0	no
3	CP21	18.08	Secondary	no	no eye test		
3	CP22	18.25	Secondary	no	no eye test	0.03	no
3	CP23	14.25	Secondary	no	HES discharged	0.9	yes
3	CP24	16.75	Secondary	no	HES discharged	0	no
3	CP25	16.83	Secondary	no	optom only	0.2	no
3	CP26	14.33	Secondary	no	no eye test	0.24	no
3	CP27	16	Secondary	no	HES discharged		
3	CP28	13.58	Secondary	no	no eye test		
3	CP29	14.17	Secondary	no	no eye test		
3	CP30	15.33	Secondary	no	no eye test	0	no
3	CP31	12.92	Secondary	no	current HES	1.3	yes
3	CP32	14.67	Secondary	no	no eye test	0.3	no
3	CP33	13.83	Secondary	no	HES discharged		
3	CP34	14.42	Secondary	no	optom only		
3	CP35	13.17	Secondary	no	no eye test		
3	CP36	13.08	Secondary	no	no eye test	0.1	no
3	CP37	16.5	Secondary	no	seen at school	-0.1	no
3	CP38	19.75	Secondary	no	optom only	-0.1	no
3	CP39	16.92	Secondary	no	HES discharged	0.4	no
3	CP40	16.22	Secondary	no	no eye test		
3	GW018	7.83	Primary	no	HES discharged	0.3	no
3	GW019	5.83	Primary	yes			
3	GW020	8.42	Primary	no	current HES	0	no
3	GW021	5.5	Primary	yes		0.6	yes
3	GW022	5.92	Primary	yes	current HES	0.7	yes
3	GW023	7.58	Primary	no	current HES	0.6	yes

3	GW024	4.83	Primary	yes	no eye test		
3	GW025	4.83	Primary	yes	current HES	0.43	no
3	GW026	3.92	Primary	no	current HES		
3	GW027	6.92	Primary	no	HES discharged		
3	GW028	10	Primary	no	no eye test	0.5	yes
3	GW029	5.75	Primary	yes	current HES		
3	GW030	4.67	Primary	yes	no eye test	0.4	no
3	GW031	7	Primary	no	current HES	0.6	yes
3	GW032	6.25	Primary	no	current HES	0.3	no
3	GW033	5.25	Primary	yes	HES discharged		
3	GW043	8.33	Primary	no	no eye test	0	no
3	GW044	8.83	Primary	no	no eye test		
3	GW045	8.75	Primary	no	no eye test		
3	GW046	10.08	Primary	no	optom only	0.1	no
3	GW047	9	Primary	no	HES discharged	0.3	no
3	GW048	10.58	Primary	no	current HES	0.6	yes
3	GW049	9.17	Primary	no	HES discharged	0.3	no
3	GW050	9.25	Primary	no	current HES		
3	GW051	7.25	Primary	no			
3	GW052	10.83	Primary	no		0	no
3	GW053	10.33	Primary	no	current HES		
3	GW054	10.17	Primary	no			
3	GW056	8.92	Primary	no	no eye test	0	no
3	GW057	4.33	Primary	yes	current HES	0.6	yes
3	GW058	4.75	Primary	yes	no eye test	0.5	yes
3	GW059	5.75	Primary	yes	current HES	0.6	yes
3	GW060	5.17	Primary	yes	no eye test		
3	GW061	6.08	Primary	no			
3	GW062	6	Primary	no	HES discharged		
3	HH001	16.67	Secondary	no	no eye test		
3	HH002	17.75	Secondary	no	no eye test	0.2	no
3	HH003	16.92	Secondary	no	HES discharged		
3	HH004	9.75	Primary	no	HES discharged		
3	HH005	11.17	Secondary	no	HES discharged	0.1	no
3	HH006	10.67	Primary	no	HES discharged		
3	HH007	13.67	Secondary	no	HES discharged		
3	HH008	13.67	Secondary	no	HES discharged	0	no
3	HH009	13.08	Secondary	no	no eye test	0.1	no
3	HH010	5.92	Primary	yes	current HES		
3	HH011	6	Primary	no	no eye test		
3	HH012	5.83	Primary	yes	current HES		
3	HH013	14.67	Secondary	no	no eye test	0	no
3	HH014	15.58	Secondary	no	optom only		
3	HH015	16.83	Secondary	no	HES discharged		
3	HH016	15.83	Secondary	no	no eye test		
3	HH017	7.92	Primary	no	HES discharged		
3	HH018	13.58	Secondary	no	HES discharged		
3	HH019	19.33	Secondary	no	current HES	0.2	no
3	HH020	8.67	Primary	no	HES discharged		
3	HH021	13.92	Secondary	no			

3	HH022	12.08	Secondary	no	HES discharged	0.4	no
3	HH023	18.42	Secondary	no	current HES		
3	HH024	13.75	Secondary	no	optom only	0.4	no
3	HH025	15.33	Secondary	no	no eye test		
3	HH026	11.25	Secondary	no	no eye test	0.1	no
3	HH027	15.08	Secondary	no	current HES		
3	HH028	17.58	Secondary	no	HES discharged		
3	HH029	13.08	Secondary	no	HES discharged		
3	HH030	7.42	Primary	no	no eye test	0	no
3	M038	12.25	Secondary	no	HES discharged		
3	M039	18.42	Secondary	no	HES discharged	0.4	no
3	M040	13.42	Secondary	no	HES discharged		
3	M041	12.67	Secondary	no	HES discharged	0.65	yes
3	M042	12.08	Secondary	no			
3	M043	18.92	Secondary	no	HES discharged		
3	M044	17.83	Secondary	no	optom only	0.23	no
3	M045	12.17	Secondary	no	no eye test		
3	M046	10.75	Primary	no	HES discharged		
3	M047	17.08	Secondary	no	HES discharged		
3	M048	12.83	Secondary	no	HES discharged	0	no
3	M049	10.83	Primary	no	HES discharged	0.1	no
3	M050	10.5	Primary	no	no eye test	0.1	no
3	M051	12	Secondary	no	no eye test	0	no
3	M052	10.92	Primary	no	no eye test		
3	M053	11.08	Secondary	no	no eye test		
3	M054	11.25	Secondary	no	HES discharged		
3	M055	17.5	Secondary	no	optom only		
3	PL046	4.5	Primary	yes	current HES		
3	PL047	6.5	Primary	no			
3	PL048	6.92	Primary	no	HES discharged	0.1	no
3	PL049	3.58	Primary	no	no eye test		
3	PL050	3.5	Primary	no	HES discharged		
3	PL051	3.92	Primary	no	current HES		
3	PL052	4.92	Primary	yes	no eye test		
3	PL053	5.92	Primary	yes	current HES		
3	PL054	10.33	Primary	no	current HES		
3	PL055	4.58	Primary	yes	no eye test		
3	PL056	3.83	Primary	no	current HES		
3	PL057	10.5	Primary	no	current HES	0.05	no
3	PL058	5.58	Primary	yes	current HES		
3	PL059	4.92	Primary	yes	current HES		
3	PL072	5.25	Primary	yes	no eye test		
3	PU030	11.08	Secondary	no	no eye test	0.1	no
3	PU031	13.08	Secondary	no	current HES		
3	T001	10.08	Primary	no	HES discharged	0.2	no
3	T002	10.67	Primary	no	no eye test	0	no
3	T003	9.5	Primary	no	no eye test	0	no
3	T004	9.17	Primary	no	no eye test	0	no
3	T005	8.83	Primary	no	HES discharged		
3	T006	11.5	Secondary	no	HES + optom	0.2	no

3	T007	10.67	Primary	no	no eye test	0	no
3	T008	10.67	Primary	no	HES + optom	0	no
3	T009	11.25	Secondary	no	no eye test	0	no
3	T011	8.17	Primary	no	no eye test	0	no
3	T012	5.25	Primary	yes	no eye test	0.2	no
3	T015	5.17	Primary	yes	no eye test		
3	T019	9.58	Primary	no	no eye test	0	no
3	T020	7.08	Primary	no	current HES		
3	T022	7.42	Primary	no	HES discharged	0	no
3	T023	7.75	Primary	no	HES discharged	0	no
3	T024	8.75	Primary	no	no eye test	0	no
3	T025	9.92	Primary	no	no eye test	0	no
3	T026	7.33	Primary	no	no eye test		
3	T027	6.5	Primary	no	no eye test	0.2	no
3	T028	6.92	Primary	no	no eye test	0.3	no
3	T029	8.83	Primary	no	optom only	0.2	no
3	T030	11.25	Secondary	no	HES + optom	0	no
3	T031	11.25	Secondary	no	HES + optom	0	no
3	T032	6.42	Primary	no	current HES	0.4	no
3	T034	5.42	Primary	yes		0.25	no
3	T035	19.25	Secondary	no	HES + optom	0.2	no
3	T038	12.08	Secondary	no	no eye test	0	no
3	T039	13	Secondary	no	HES discharged	0.2	no
3	T040	14.33	Secondary	no	no eye test	0.6	yes
3	T041	13.83	Secondary	no	optom only	0.2	no
3	T042	13.67	Secondary	no	no eye test	0	no
3	T043	14.75	Secondary	no	no eye test	0	no
3	T044	14.17	Secondary	no	no eye test	0	no
3	T045	12	Secondary	no	no eye test	0	no
3	T047	6.25	Primary	no	no eye test	0	no
3	T048	7	Primary	no	no eye test	0.2	no
3	T050	5	Primary	yes	no eye test	0.3	no
3	T052	16.75	Secondary	no	no eye test	0	no
3	T053	17.67	Secondary	no	no eye test	0.2	no
3	T054	13.33	Secondary	no	no eye test	0	no
3	T055	14.75	Secondary	no	optom only	0	no
3	T056	13.83	Secondary	no	HES + optom	0	no
3	T058	5.75	Primary	yes	HES discharged		
3	T059	12	Secondary	no	optom only	0	no
3	T061	11.92	Secondary	no	no eye test		
3	T062	13.67	Secondary	no	HES discharged	0	no
3	T063	11.58	Secondary	no	current HES	0.4	no
3	T064	13.5	Secondary	no	HES + optom	0	no
3	T065	8.5	Primary	no			
3	T066	13.17	Secondary	no	no eye test	0	no
3	T067	13.42	Secondary	no	no eye test	0	no
3	T068	10.42	Primary	no	no eye test	0	no
3	T072	11.83	Secondary	no	optom only	0	no
3	VS219	4.2	Primary	yes	no eye test		
3	VS220	5.2	Primary	yes	no eye test	0.1	no

3	VS221	11.5	Secondary	no	no eye test	-0.03	no
3	VS222	4.5	Primary	yes	HES discharged	1.1	yes
3	VS223	11.3	Secondary	no	HES discharged	0.18	no
3	VS224	4.6	Primary	yes	HES discharged		yes
3	VS225	9.5	Primary	no		0	no
3	VS226	4.4	Primary	yes		0.9	yes
3	VS227	8.9	Primary	no		0.28	no
3	VS228	11.8	Secondary	no		0.03	no
3	VS229	5.9	Primary	yes	current HES		
3	VS230	5.1	Primary	yes	current HES		
3	VS231	14.3	Secondary	no	no eye test	0.05	no
3	VS232	7.4	Primary	no	no eye test	0.9	yes
3	VS233	4.7	Primary	yes	no eye test	0.63	yes
3	VS234	13.9	Secondary	no		0.9	yes
3	VS235	4.6	Primary	yes	no eye test	1.1	yes
3	VS236	4.3	Primary	yes	no eye test	0.6	yes
3	VS237	4.9	Primary	yes	no eye test		
3	VS238	5	Primary	yes	current HES	0.8	yes
3	VS239	4.9	Primary	yes	current HES		
3	VS240	11.4	Secondary	no	no eye test	-0.1	no
3	VS241	13.8	Secondary	no	no eye test	0.23	no
3	VS242	11.6	Secondary	no	no eye test	0.03	no
3	VS243	11.8	Secondary	no	no eye test	-0.03	no
3	VS244	5.4	Primary	yes	current HES		
3	VS245	17.1	Secondary	no			
3	VS246	7.1	Primary	no			
3	VS247	11.4	Secondary	no		0.65	yes
3	VS248	4.9	Primary	yes	HES discharged	1	yes
3	VS249	12.2	Secondary	no	HES discharged	0.15	no
3	VS250	5.8	Primary	yes	current HES		
3	VS252	6.2	Primary	no	no eye test	0.6	yes
3	VS253	4.5	Primary	yes	no eye test	0.6	yes
3	VS254	10.9	Primary	no	current HES	0.3	no
3	VS255	5.3	Primary	yes	current HES		
3	VS256	5	Primary	yes	current HES	0.8	yes
3	VS257	5.2	Primary	yes	no eye test	-0.2	no
3	VS258	14.8	Secondary	no	HES discharged	0	no
3	VS259	8.4	Primary	no	HES discharged	0.55	yes
3	VS260	18.3	Secondary	no	no eye test	0.2	no
3	VS261	19.5	Secondary	no	no eye test	0.1	no
3	VS262	8.5	Primary	no		0.15	no
3	VS263	9.9	Primary	no		0.05	no
3	VS264	10.7	Primary	no			
3	VS265	9.7	Primary	no		0.05	no
3	W058	10	Primary	no	no eye test	0.1	no
3	W059	9.33	Primary	no	current HES	0.1	no
3	W063	8.75	Primary	no	HES discharged	0.2	no
3	W064	10.83	Primary	no	current HES	0.4	no
3	W065	9.42	Primary	no	optom only	0	no
3	W066	11	Secondary	no	seen at school		

3	W067	9.08	Primary	no	no eye test		
3	W068	8.25	Primary	no	no eye test	0.2	no
3	W069	8.25	Primary	no	current HES	0.1	no
3	W070	8.08	Primary	no	no eye test	0.1	no
3	W071	8.42	Primary	no	no eye test	0.2	no
3	W072	8	Primary	no	no eye test		
3	W073	11.25	Secondary	no	current HES	1	yes
3	W074	10.75	Primary	no	current HES		yes
3	W075	11.17	Secondary	no	no eye test		
3	W076	2.58	Primary	no	current HES	0.4	no
3	W077	10.17	Primary	no	no eye test	0.3	no
3	W078	6.75	Primary	no	HES discharged		
3	W079	8	Primary	no	current HES		yes
3	W080	9.67	Primary	no	HES discharged		
3	W081	10.08	Primary	no	no eye test	0.2	no
3	W082	6.83	Primary	no	current HES	0.3	no
3	W083	10.33	Primary	no	HES discharged	0.2	no
3	W084	7.75	Primary	no	no eye test		
3	W085	10.5	Primary	no	no eye test	0.2	no
3	W086	8.42	Primary	no	no eye test	0	no
3	W087	10.08	Primary	no	no eye test		
3	W088	8.58	Primary	no	HES discharged		
3	W089	12.17	Secondary	no	no eye test		yes
3	W090	7.42	Primary	no	HES discharged	1.1	yes
3	W091	4.17	Primary	yes	no eye test		
3	W092	3.25	Primary	no	current HES		
3	W093	4.25	Primary	yes	no eye test		
3	W094	4.67	Primary	yes	no eye test	0.5	yes
3	W095	4.67	Primary	yes	no eye test	0.2	no
3	W096	3.33	Primary	no	current HES		yes
3	W097	4.42	Primary	yes	current HES		yes
3	W098	4	Primary	yes	current HES		yes
3	W099	4.83	Primary	yes	no eye test		
3	W100	4.92	Primary	yes	no eye test		
3	W101	5.75	Primary	yes	no eye test		
3	W102	4.83	Primary	yes	no eye test		
3	W103	4.08	Primary	yes	current HES	1	yes
3	W104	4.83	Primary	yes	no eye test	0.1	no
3	W105	7.67	Primary	no	no eye test		
3	W106	8.25	Primary	no			
3	W107	9.33	Primary	no	no eye test	0	no
3	W108	9.92	Primary	no	no eye test		
3	W109	8.42	Primary	no	HES discharged	0.4	no
3	W110	9.42	Primary	no	no eye test		
3	W111	7.17	Primary	no	no eye test		
3	W112	11.42	Secondary	no	no eye test	0.3	no
3	W113	7.17	Primary	no	no eye test		
3	W115	9.92	Primary	no	no eye test	0	no
3	W116	8.33	Primary	no	no eye test		
3	W117	6.83	Primary	no	no eye test	0.1	no

3	W118	5	Primary	yes	no eye test		
3	W119	5.75	Primary	yes	no eye test		
3	W120	5.33	Primary	yes	no eye test		
3	W121	3.58	Primary	no	current HES		
3	W122	8.17	Primary	no	optom only	0.13	no
3	W123	7.67	Primary	no	no eye test	0	no
3	W124	8.33	Primary	no	HES discharged	0	no
3	W125	12.58	Secondary	no		0.1	no
3	W260	12	Secondary	no	HES discharged	0.5	yes
3	W261	9.25	Primary	no	no eye test		
3	W262	9.08	Primary	no	current HES		
4	CP100	14.32	Secondary		no eye test		
4	CP101	15.53	Secondary		HES discharged		
4	CP102	12.09	Secondary		no eye test	0.2	no
4	CP103	19.11	Secondary		no eye test	-0.1	
4	CP104	16.12	Secondary		no eye test		
4	CP105	19.02	Secondary		no eye test	0	no
4	CP106	18.99	Secondary		HES discharged		
4	CP107	18.02	Secondary		no eye test	0	no
4	CP108	19.17	Secondary		HES discharged		
4	CP109	19.12	Secondary		no eye test	0	no
4	CP110	18.93	Secondary		HES discharged		
4	CP111	17.31	Secondary			0.3	no
4	CP41	14.74	Secondary		optom only	0	no
4	CP42	15.52	Secondary		optom only	0	no
4	CP43		Secondary			0.2	no
4	CP44	13.57	Secondary			-0.1	no
4	CP45		Secondary		optom only	0	no
4	CP46	15.63	Secondary		no eye test	0.2	no
4	CP47	14.64	Secondary		HES discharged	0.6	yes
4	CP48	16.5	Secondary		optom only	0.02	no
4	CP49	13.76	Secondary		optom only	0.4	no
4	CP50	15.23	Secondary		no eye test		
4	CP51	15.5	Secondary		optom only	0.2	no
4	CP52	12.85	Secondary		no eye test	0.2	no
4	CP53	14.7	Secondary		HES discharged	0.3	no
4	CP54	15.76	Secondary		no eye test	0	no
4	CP55	15.86	Secondary		no eye test	0	no
4	CP56	16.9	Secondary		no eye test		
4	CP57	14.73	Secondary		no eye test		
4	CP58	15.02	Secondary		HES + optom	0.46	no
4	CP59	13	Secondary		no eye test		
4	CP60	15.89	Secondary		HES + optom	0	no
4	CP61	14.93	Secondary		HES discharged	0	no
4	CP62	18.03	Secondary		no eye test	0.06	no
4	CP63	14.77	Secondary		no eye test		
4	CP67	14.58	Secondary		no eye test	0	no
4	CP68	14.69	Secondary		optom only	0	no
4	CP69	15.64	Secondary		no eye test	0.15	no
4	CP70	15.26	Secondary		no eye test		

4	CP71	16.87	Secondary		no eye test	0.1	no
4	CP72	15.97	Secondary		optom only	0.33	no
4	CP73	16.47	Secondary		HES discharged	0.2	no
4	CP74	15.85	Secondary		no eye test	0.3	no
4	CP75	14.97	Secondary		HES discharged		
4	CP76	12.54	Secondary		HES + optom	0	no
4	CP77	17.83	Secondary		no eye test		
4	CP78	14.35	Secondary		HES discharged	0	no
4	CP79	11.94	Secondary		no eye test	0	no
4	CP80	12.02	Secondary		optom only	0	no
4	CP81	18.6	Secondary			0	no
4	CP82	16.71	Secondary		no eye test		
4	CP83	14.14	Secondary		no eye test		
4	CP84	14.35	Secondary		HES + optom	0.45	no
4	CP85	12.61	Secondary		optom only	0.03	no
4	CP86	17.31	Secondary		optom only	0	no
4	CP87	15.1	Secondary		seen at school	0.6	yes
4	CP88	12.08	Secondary		HES discharged	0.2	no
4	CP89	14.06	Secondary		no eye test	0	no
4	CP90	17.47	Secondary		no eye test	-0.1	no
4	CP91	14.07	Secondary		optom only	0.06	no
4	CP92	18.93	Secondary		optom only	0	no
4	CP93	14	Secondary		HES discharged		
4	CP94	19.34	Secondary		HES discharged	0	no
4	CP95	15.84	Secondary		HES discharged		
4	CP96	12.55	Secondary		no eye test	-0.1	no
4	CP97	12.4	Secondary			-0.1	no
4	CP98	14.91	Secondary		no eye test	-0.1	no
4	CP99	19.6	Secondary		seen at school		
4	GW046	6.17	Primary	no	current HES	0.3	no
4	GW047	8.14	Primary	no	current HES		
4	GW048	8.61	Primary	no	current HES	0.2	no
4	GW063	6.63	Primary	no	HES discharged	0.2	no
4	GW064	15.74	Secondary	no	HES discharged		
4	GW065	5.97	Primary	yes	current HES		
4	GW066	6.54	Primary	no			
4	GW067	10.77	Primary	no	no eye test		
4	GW068	8.37	Primary	no	no eye test		
4	GW069	8.9	Primary	no	no eye test	0.2	no
4	GW070	8.18	Primary	no	current HES		
4	GW071	6.4	Primary	no	no eye test		
4	GW072	6.25	Primary	no	HES discharged	0.3	no
4	GW073	7.53	Primary	no	current HES		
4	GW074	4.16	Primary	yes	no eye test		
4	GW075	4.79	Primary	yes	no eye test		
4	GW076	4.19	Primary	yes	no eye test		
4	GW076	5.56	Primary	yes	current HES	0.4	no
4	GW077	5.22	Primary	yes	current HES	0.4	no
4	GW078	6.42	Primary	no	current HES		
4	GW079	7.82	Primary	no			

4	GW080	4.41	Primary	yes	current HES		
4	GW081	8.37	Primary	no	current HES		
4	GW082	5.03	Primary	yes	no eye test	0.3	no
4	GW083	7.37	Primary	no	no eye test		
4	GW084	14.7	Secondary	no	current HES	0.3	no
4	GW085	4.82	Primary	yes	HES discharged	0	no
4	HH030	5.67	Primary	yes	no eye test	0.3	no
4	HH031	5.3	Primary	yes	current HES	0.3	no
4	HH032	8.72	Primary	no	no eye test	0.3	no
4	HH033	6.51	Primary	no	no eye test	0.3	no
4	HH034	6.55	Primary	no	current HES		
4	HH035	5.34	Primary	yes	current HES		
4	HH036	11.33	Secondary	no	current HES		
4	HH037	12.96	Secondary	no	HES + optom		
4	HH038	11.04	Secondary	no	no eye test		
4	HH039	6.18	Primary	no	no eye test		
4	HH040	13.85	Secondary	no	no eye test	0.2	no
4	HH041	17.37	Secondary	no	HES discharged	0.2	no
4	HH042	16.47	Secondary	no	no eye test	0	no
4	HH043	11.88	Secondary	no	no eye test		
4	M042	13.6	Secondary	no			
4	M057	10.6	Primary	no	no eye test		
4	M058	16.38	Secondary	no	no eye test		
4	M059	19.41	Secondary	no			
4	M060	13	Secondary	no		0.1	no
4	M061	13.65	Secondary	no	optom only	0.2	no
4	M062	16.14	Secondary	no	HES discharged	0	no
4	M063	11.43	Secondary	no	no eye test	0.3	no
4	M063	12.88	Secondary	no	current HES	0.2	no
4	N01	11.9	Secondary	no	no eye test	0.1	no
4	N02	12.62	Secondary	no	optom only	0.26	no
4	N03	12.13	Secondary	no	current HES	0.7	yes
4	N04	12.62	Secondary	no	optom only	0.1	no
4	N05	12.58	Secondary	no	optom only	0.1	no
4	N06	11.76	Secondary	no	optom only	0.34	no
4	N07	11.73	Secondary	no		0.3	no
4	N08	11.77	Secondary	no	current HES	0.7	yes
4	N09	12.1	Secondary	no	current HES		
4	N10	12.2	Secondary	no	no eye test		
4	N11	12.65	Secondary	no	no eye test	0.3	no
4	N12	12.56	Secondary	no	optom only		
4	N13	12.84	Secondary	no	current HES	0.3	no
4	N14	13.06	Secondary	no	optom only	0.8	yes
4	N15	13.42	Secondary	no	optom only	0.1	no
4	N16	12.6	Secondary	no	optom only	0.3	no
4	N17	13.48	Secondary	no	HES discharged		
4	N18	12.39	Secondary	no		0.7	yes
4	N19	13.6	Secondary	no	current HES		

4	N20	13.08	Secondary	no	current HES		
4	N21	12.83	Secondary	no	no eye test		
4	N22	14.4	Secondary	no	HES discharged		
4	N23	13.63	Secondary	no	optom only	0.3	no
4	N24	13.52	Secondary	no		0.3	no
4	N25	12.88	Secondary	no		0.16	no
4	N26	14.12	Secondary	no	HES + optom	0.4	no
4	N27	12.11	Secondary	no	current HES		
4	N28	13.95	Secondary	no		0.1	no
4	N29	14.19	Secondary	no		0.3	no
4	N30	13.72	Secondary	no		0	no
4	N31	14.65	Secondary	no	optom only	0.2	no
4	N32	14.97	Secondary	no		0.3	no
4	N33	14.23	Secondary	no	current HES		
4	N34	15.31	Secondary	no	optom only	0.2	no
4	N35	17.39	Secondary	no	no eye test		
4	N36	15.73	Secondary	no	no eye test	0	no
4	N37	15.44	Secondary	no		0.1	no
4	N38	14.78	Secondary	no		0.3	no
4	OK597	12.11	Secondary	no	current HES		yes
4	OK603	12.48	Secondary	no	HES + optom	0.32	no
4	OK605	11.99	Secondary	no	current HES		yes
4	PL060	4.88	Primary	yes	no eye test	0.4	no
4	PL061	9.46	Primary	no	no eye test	0	no
4	PL062	14.21	Secondary	no	current HES		
4	PL063	10.87	Primary	no	HES discharged	0.1	no
4	PL064	4.12	Primary	yes	no eye test		
4	PL065	11.19	Secondary	no	no eye test	0	no
4	PL066	5.89	Primary	yes	no eye test	0.1	no
4	PL067	5.4	Primary	yes	no eye test	0.2	no
4	PL068	5.11	Primary	yes	no eye test		
4	PL069	9.63	Primary	no	HES discharged		
4	PL070	7.41	Primary	no	current HES		
4	PL071	6.32	Primary	no	no eye test	0.2	no
4	PU031	18.24	Secondary	no	current HES	0	no
4	PU032	12.54	Secondary	no	current HES	0	no
4	PU033	12.34	Secondary	no	current HES	0	no
4	PU034	18.64	Secondary	no	no eye test	-0.1	no
4	PU035	16.76	Secondary	no	no eye test	0	no
4	PU036	12.44	Secondary	no	HES discharged	0	no
4	PU037	11.85	Secondary	no		0.05	no
4	VS335	9.65	Primary	no	no eye test	0.25	no
4	VSoptix 11(	7.38	Primary	no	no eye test		
4	VSOptix 22	5.44	Primary	yes	current HES		
4	VSOptix 22	7.6	Primary	no	HES + optom		
4	VSOptix 23	10.8	Primary	no	no eye test	0.05	no
4	VSoptix 23!	5.4	Primary	yes	HES discharged		
4	VSOptix 28	8.04	Primary	no	HES discharged		
4	VSOptix 29	8.02	Primary	no	no eye test	0	no
4	VSOptix 32	13.08	Secondary	no	optom only	0.15	no

4	VSOptix 33	14.08	Secondary	no	no eye test		
4	VSOptix 34	11.91	Secondary	no	no eye test	0.3	no
4	VSOptix110	4.87	Primary	yes	no eye test	0	no
4	VSOptix111	5.73	Primary	yes	HES discharged		
4	VSOptix224	6.92	Primary	no	current HES		
4	VSOptix236	5.14	Primary	yes	no eye test	0.05	no
4	VSOptix237	4.76	Primary	yes	no eye test	0.1	no
4	VSOptix241	6.6	Primary	no	current HES		
4	VSOptix260	7.13	Primary	no	no eye test	0.4	no
4	VSoptix311	10.36	Primary	no	HES discharged	0.03	no
4	W123	11.09	Secondary	no	current HES		yes
4	W124	7.21	Primary	no	HES discharged	0.3	no
4	W124	10.65	Primary	no	current HES	0.1	no
4	W125	4.3	Primary	yes	no eye test		yes
4	W126	6.5	Primary	no	no eye test	0.2	no
4	W127	10.11	Primary	no			yes
4	W128	7.06	Primary	no	HES discharged	0.3	no
4	W129	9.74	Primary	no	no eye test	0.1	no
4	W130	7.24	Primary	no	no eye test	0.3	no
4	W131	4.72	Primary	yes	no eye test	0.3	no
4	W132	6.67	Primary	no	current HES	0.3	no
4	W133	4.94	Primary	yes	no eye test		
4	W134	7.51	Primary	no	no eye test		
4	W135	10.94	Primary	no	no eye test	0	no
4	W136	6.93	Primary	no	no eye test	0.3	no
4	W137	6.83	Primary	no	no eye test		
4	W138	10.72	Primary	no	no eye test	0	no
4	W139	4.52	Primary	yes	HES discharged		
4	W140	4.54	Primary	yes	no eye test	0.1	no
4	W141	12.26	Secondary	no	HES discharged		yes
4	W142	5.8	Primary	yes	no eye test		
4	W143	6.23	Primary	no	no eye test	0	no
4	W144	5.21	Primary	yes	no eye test		
4	W145	10.49	Primary	no	no eye test		
4	W146	5.18	Primary	yes	no eye test		
4	W147	9.04	Primary	no	no eye test		
4	W148	8.28	Primary	no	no eye test	0.3	no
4	W149	7.13	Primary	no	no eye test		yes
4	W150	9.36	Primary	no	HES discharged	0	no
4	W151	5.08	Primary	yes	optom only	0.3	no
4	W152	11.49	Secondary	no	no eye test		
4	W153	6.86	Primary	no	HES discharged	0	no
4	W154	7.65	Primary	no	no eye test		
4	W155	10.17	Primary	no	no eye test		
4	W156	10.95	Primary	no	optom only	0.36	no
4	W157	4.84	Primary	yes	no eye test		
4	W158	8.02	Primary	no	HES discharged		yes
4	W159	8.73	Primary	no	seen at school	0.43	no
4	W160	9.4	Primary	no	no eye test	0.1	no
4	W161	6.87	Primary	no	no eye test	0.1	no

4	W162	6.19	Primary	no	no eye test		
4	W163	6.33	Primary	no	no eye test		
4	W164	7.88	Primary	no	no eye test	0.4	no
4	W165	6.21	Primary	no	no eye test	0.3	no
4	W166	6.72	Primary	no	no eye test	0.4	no

Reason no VA	Strabismus	Cyclo used	Rsphere	Rcyl	Raxis	Lsphere	Lcyl
pass	no		0	-0.5	180	0	-0.5
fail	no		0	-1.5	180	0	-1.5
fail	yes		0	0		0	
fail	no		-1	-0.5	180	-0.5	-0.5
fail	yes		4	-3	150	4.5	-3
fail	yes		1	-0.5	180	1	-0.5
	no		3	-0.5	180	3	-0.5
pass	no		0	0		0	
	yes		0				
pass	no		0	-2	175	0	-2
pass	no		0.5	0		0.5	
pass	no		1	0		1	
fail	yes		0.75	0		0.75	
fail	no		-2.25	0		-4.5	0
	no		3	0		3	
pass	no		1.5	-1.5	180	1.5	-1.5
	no		0	0		0	
	yes		0.5	0		0.5	
	no		0	0		0	
	no		0				
	no		0	0		0	0
	yes		1	0		1	0
	yes		-0.5	-3	180	-0.5	-3
	no		-2.5	-2	180	-0.5	-2
	yes		-30	0		-30	0
	no		-2.5	-1	180	-1.5	-1
fail	no		0	-3.5	90	-1.5	-3
	no		1	0		1	0
	no		1.5	-2	90	1.5	-2
	no		0.5	-1	180	0.5	-1
	yes		4.5	0		3	0
	no						
	no		1	0		1	0
	yes		0.5	-2.5	10	0.5	-2.5
	no	yes	-5	0		-5	0
	yes		3	0		3	0
	yes		5	0		5	0
	no		2	-1	180	2	-1
	no		7	-2	180	5	0
pass	no						
fail	no						
	no		2	0		2	0
	yes		-0.5	-1	180	-0.5	-1
fail	no	yes	3.5	-0.75	180	3.5	-0.75
	no		0.5	-0.5	180	0.5	-0.5
fail	no		-0.5	0		-0.5	0

fail	no		0.75	0		0.75	0
fail	no		6	-2	70		
fail	no						
pass	no						
pass	no						
fail	no		1.25	0		1.25	0
	no		2	-2	180	2	-2
fail	no		2	-3.5	180	3	-3.5
fail	no		-4.5	-4	15	-4.5	-3
fail	no						
fail	no		-2.5	0		-1.5	0
fail	yes	yes	2	-1	165	2	-1
fail	no		1	-1	180	1	-1
fail	yes		0.75	0		0.75	0
fail	no	yes	2	-3	180	2	-3
fail	no		0	-1	180	0	-1
	no		0	-0.5	180	0	-0.5
fail	no		0	-0.5	180	0	-0.5
	no						
pass	no		4.5	-2.5	180	4.5	-2.5
pass	no		2.5	-1.5	10	3.25	-1.5
	no		0	-0.75	180	0	-0.75
	no		0.5	0		0.5	0
	no		0.5	-0.5	90	0.5	-0.5
	yes		0.5	-2	180	0	-2
fail	no		0.5	0		0.5	0
fail	no	yes	-0.5	-1	180	-0.5	-2.5
	yes		0	0		0	-1
pass	no		0	-0.5	180	0	-0.5
	no		-1	0		-0.5	-1
	yes		2	0		-0.5	-2
fail	no						
fail	yes		2.5	0		2.5	0
	yes						
pass	no						
fail	yes		-0.5	-1	180	-0.5	0
pass	no		2	-1	90	2	-1
fail	no	yes	2	-0.75	20	2.75	-0.75
	no		1.5	0		1.5	0
	yes		-2	-2	20	-0.5	0
	no						
fail	yes		0	-1	180	0	0
fail	no		-16	-2	180	-16	-2
fail	yes		4	-1.5	10	4	-1.5
fail	yes	yes	0			2	0
fail	yes		1	-1	10	1	-1
fail	no		2			2	0
pass	no		0			0	0
	no		0			0	0
pass	no		0			0	0

fail	no		1			1	0
fail	no		0.5	-0.5	90	0.5	-0.5
	no		0.5			0.5	0
	no		1.5			1.5	0
fail	yes		2.5	-1	180	3	-1
fail	no		2	-1	180	2	-1
fail	no	yes	1	-2	170	1	-2.5
	yes		2	-0.5	180	2.5	-0.5
pass	no		0			0	0
pass	no		0			0	0
fail	yes		0.75			0.75	0
fail	no		0			0	0
	no		0	0		0	0
	no		0.75	0		0.75	0
fail	no		1	0		1	0
fail	no		-10	-2	10	-12.5	-2
fail	no		0	0		0	0
	no		-14.5	-1	180	-13	-1
	yes		3	-2.5		3	-3
fail	no		0.5	-0.75	180	0.5	-0.75
pass	no		0	-3	5	0	-3
fail	no		1			1	0
	yes		3.5			3.5	0
	yes		-17.5	-2.5	180	-17.5	-3.5
pass	no		2.75	-1.5	10	2.5	-1.75
fail	no		4.5	-1	150	3.75	0
pass	no		0.5			0.5	0
fail	no		-0.5			-0.5	0
pass	no		0			0	0
pass	no		0.5			0.5	0
pass	no		0			0	0
	no		0.5			0.5	0
pass	no		1.25			0.5	0
	no		2.5	-2	20	2	-2
fail	no		0.5	-0.5	180	0.5	-0.5
fail	no		0.75			0.75	0
pass	yes		0	-3	180	0	0
	no		1.5	-2	170	1.5	-2
fail	no		0			0	0
	no		1			1	0
fail	no	yes	-17	-2	20	-0.5	0
pass	no		0			0	0
fail	no					0	0
fail	yes		3.5	-2	5	3	-1.5
fail	no		0	-1.5	50	0	-1.5
pass	yes		0			0	0
pass	no		3.5	-1.5	90	0.75	-0.75
	no		1			1	0
fail	yes		0	-0.5	180	0	-0.5
fail	no		-13.5	0		-13.5	0

pass	yes		0	-0.5	10	1.5	-1
pass	no		0	0		0	0
pass	no		0	0		0	0
fail	yes		-3	0		-1	0
	no		1	-2	180	1	-2
	no		0	0		0	0
	no	yes	4	0		4	0
fail	no		2.25	-2.25	105	2.25	-2.25
fail	no		-2	-1	180	-7.5	-1
	yes		0.5	0		0.5	0
fail	no		0.5	0		0.5	0
fail	no		0.5	-0.75	90	0.5	-0.75
fail	yes		0	0		0	0
fail	no		1	-1	180	1	-1
	no		1	0		1	0
pass	no		0	0		0	0
	yes		6.5	-1.5	180	6.5	-1.5
fail	yes		-1	0		-1	0
pass	no		0	0		0	0
pass	no		0.5	0		0.5	0
fail	yes		-2	-1	180	-2	-1
fail	yes		-13	-1.75	180	-18	0
	yes		0.5	0		0.5	0
	yes	yes	2.5	-0.5	180	2.5	-0.5
	no		0	0		0	0
	yes		2	-3	180	2	-3
	no		1	-1	180	1	-1
fail	yes		0.5	-2	180	0.5	-2
	yes		1	0		1	0
pass	no		0.5			0.5	0
pass	no		0	0		0	0
pass	no		0.75	0		0.75	0
fail	no		3	-0.75	90	4	-0.75
pass	no		0	0		0	0
	yes		1	-2	180	1	-2
fail	no		0.5	0		0.5	0
fail	yes		-2	-0.75	180	-3	-2
fail	no		0.5			0.5	0
fail	no		-1	0		-1	0
pass	yes		0.75	0		0.75	0
pass	yes		2.5	-2	180	4	-3.5
fail	no		0.5	0		0.5	0
pass	no		0.5	0		0.5	0
fail	no	yes	0	-1	15	0	-0.75
fail	no		0	0		0	0
pass	no		0.5	-0.75	180	0.5	-0.75
	yes		1	-1	180	1	-1
fail	yes		0	-0.75	180	0	-0.75
pass	no		0.5	0		0.5	0
fail	yes		-1	-0.5	180	-2	-2.5

pass	no		0.5	-1	180	0.5	-1
pass	no		1.5	-1	180	1.5	-1
fail	no		3	-2.5	168	2	-1.5
fail	no	yes	1	-1.5	180	1	-1.5
pass	no		0.5	0		0.5	0
pass	no		1	-1	180	1	-1
fail	yes		0	-0.75	15	0	-2.5
fail	no		-6.5	-1.5	10	-6	-2.5
fail	no		3.5	-5	180	3.5	-6
	no		0	0		0	0
fail	yes		3	-1.5	10	3	-1.5
fail	no		-7	-3.5	170	-6	-3
pass	no		0.75			0.75	0
	no		1			1	0
fail	yes	yes	7.5	-2	180	5.5	-1
fail	yes		-12	-2.5	165	-10	-1.75
fail	no		0	0		0	0
pass	no		0.5	0		0.5	0
pass	no		0.5	-1	180	0.5	-1
	no		0.5	0		0.5	0
	yes		-10.5	-2	180	-11	-2
fail	yes		-3	-1	180	-0.5	-2
	yes		0	0		0	0
pass	no		0	0		0	0
	no		-0.5	0	0	-0.5	0
	no		0.25	0	0	0.25	0
	no		0	0	0	0	0
fail	no		0.5	-0.5	110	0.5	0
	no		0	0	0	0	0
	no		-1	-0.5	180	0.5	-2.5
fail	no		1.5	-0.5	180	1.5	-0.5
	no		0	0	0	0	0
fail	no		0	0	0	0	0
fail	no		0	-0.75	180	0	-0.75
	no		1	-1.5	180	0	0
	no		1.5	0	0	1.5	0
fail	no		0	0	0	0	0
	no		0	0	0	0	0
fail	no		0.25	0	0	0.25	-0.75
	no		0	0	0	0	0
	yes		-3.5	-1	180	-3.5	-1.5
	yes		1	-4	180	0	-4
fail	no		-1.5	-1.5	180	-1.5	-1.5
	yes						
fail	no		0	0	0	2.5	-0.5
	no		0.5	0	0	0.5	0
fail	no		0.5	0	0	0.5	0
pass	no		0.5	0	0	0.5	0
pass	no		0.5	-1	180	0.5	-1
	no		0.5	0	0	0.5	0

	no		0.5	0	0	0.5	
fail	no		3.5	-2	180	3.5	-2
	no		0	0	0	0	0
	yes		-9.5	-4	180	-9.5	-4
fail	no		-6.5	-1	180	-6.5	-1
pass	no		-0.5	-0.5	180	-0.5	-0.5
	no		0.25	0	0	0.25	0
fail	no						
	yes		1	0		1	0
fail	no		1	-3	180	1	-3
fail	no		-0.5	-2	90	-1	-1
fail	yes		3	-1.5	180	3	-1.5
	yes		1	-1	180	1	-1
fail	no		0	0		0	0
	yes		0	0		0.75	0
fail	no		2.5	0		2.5	0
	no		2	-2	180	2	-2
pass	no		0.5	0		0.5	0
	no		1	0		1	0
	no		5	0		6	0
fail	no		1	0		1	0
	no		4	0		4	0
fail	no		0.5	0		0.5	0
fail	yes		3.5	0		3.5	0
fail	no	yes	-3	-1	180	1	-1
fail	no		0.5	0		0.5	0
	yes		1	-2	180	1	-2
fail	no		2	0		2	0
	no		1	0		1	0
pass	no		0	0		0	0
fail	no		0.5	0		0.5	0
fail	no		-4	0		-4	0
fail	yes		0.5	0		0.5	0
fail	yes		4.5	-1	180	4.5	-1
	no		1.5	-1.5	180	1.5	-1.5
fail	no		0	0		0	0
	no		0.5	0		0.5	0
pass	no		0.5	0		0.5	0
	no		-10	-1.5	180	-10	-1.5
fail	yes		0	0		-0.5	0
	yes		0.5	0		0.5	0
fail	yes		0	-4.5	12	2.5	-3.5
	no		0	0		0	0
pass	yes		-2.5	0		-2.5	0
	yes		6.5	-1	45	6.5	-1
fail	no		-0.5	-2.5	125	-2	-2
fail	no		0	0		0	0
fail	no		1	-4.75	5	1	-3.5
fail	yes		0.5	0		0.5	0
	no		0	0		0	0

fail	yes		0.5	-1.5	80	1	-2.5
fail	no		7	-1.5	5	7	-2
fail	no		0	0		0	0
pass	no		0.5	0		0.5	0
fail	yes		4.5	-1	10	4.5	-1
	no		3	0		3	0
	no		0.5	-0.75	180	0.5	-0.75
pass	no		1	-1	180	1	-1
pass	yes		0.5	-1	80	0.75	-0.5
pass	no		0	0		0	0
fail	no		0	0		0	0
fail	no		4.5	-3	180	4	-3
pass	no		-1.75	-0.5	170	-1.75	-1
pass	yes		-1	-4	5	-1.5	-4
fail	yes		7	-1	180	7	-1
fail	no		1	0		1	0
fail	no		0	0		0	0
fail	yes		0	-2	180	0	-2
fail	no		0	-4	170	0	-4
pass	yes		0.5	0		0.5	0
fail	no		-2.5	0		-2.5	0
fail	yes		-5	-0.5	170	-2	-0.5
	no		0	0		0	0
pass	no		1	0		4	0
pass	no		0	0		0	0
	no		0.5	0	1	0	0
fail	no		-1	-2	7	-1	-2.25
pass	no		2.5	0		2.5	0
pass	no		0	0		0	0
fail	no		1.5	-1.5	180	1.5	-1.5
	no		0	0		0	0
pass	no		0	0		0	0
	no		0.5	0		0.5	0
fail	yes		-9.5	-1.75	20	-9.5	-1.75
fail	no		0	-2.5	180	0	-2.5
	yes	yes	1	0		2	0
pass	no		0	0		0	0
fail	no	yes	1.75	0		1.75	0
pass	no		0	0		0	0
fail	no		0	0		0	0
	yes		0	-3.5	180	0	-2
fail	no		2	0		2	0
	yes		-1	-1.5	60	-2	-3
fail	yes		0.5	-1	180	0	-1
fail	yes	yes	4.5	0		4.5	0
pass	no		0	0		0	0
pass	no		0.5	0		0.5	0
	no		0	0		0	0
	no		1	-4	180	0	-3
	yes		2.5	-1.5	180	2.5	-1.5

fail	yes	yes	4	0		4	0
fail	no		1.5	0		1.5	0
pass	no		0	0		0	0
	no		0	0		0	0
pass	no		0.5	0		0.5	0
	no		2.5	0		2.5	0
	yes		-4.5	-3.5	180	-3.5	-3.5
	no		0	0		0	0
	no		0.5	0		0.5	0
fail	no		0	0		0	0
	no		1	0		1	0
	no	yes	-6	0		-6	0
fail	yes		-1	-2.25	180	-0.5	-1.5
	no		0	0		0	0
	no		0	0		0	0
fail	no		0	0		0	0
	yes		0.5	0		0.5	0
pass	no		-1	0		-1	0
fail	yes		0.5	0		0.5	0
	no	yes	1	0		1	0
	no		0.5	0		0.5	0
pass	no		0	0		0	0
pass	no		0	0		0	0
pass	no		1.5	0		1.5	0
	no		1	0		1	0
	no		1.5	-1	180	1.5	-1
pass	no		1	-0.75	180	-1	-0.75
fail	no		1	0		1	0
	no		0.5	-1	90	0.5	-1
fail	no		0	0		0	0
pass	no		-1	0		-1	0
	no		1.5	-1.25	90	1.5	-1.25
fail	no		0.5	0		0.5	0
fail	no		-1	0		-1	0
	no		-1.5	-3.5	180	-1.5	-3.5
pass	no		1	0		1	0
pass	no		-0.5	0		-0.5	0
	yes		-7	-3	180	-3	-3
	no		1	-0.5	180	1	-0.5
fail	no		0.5	-2	180	0.5	-2
fail	no		-1	0		-1	0
	no		3	-3.5	180	3	-3.5
	no		0.75	0		0.75	0
	yes		3	0		3	0
	yes		3	0		3	0
fail	no		0.5	0		0.5	0
	no		0.5	0		0.5	0
pass	no		0	0		0	0
	yes		5.5	-0.75	180	4.5	-0.75
pass	no		1	-0.75	180	1	-0.75

pass	no		0.5	0		0.5	0
	yes		-8	-0.5	180	-8	-0.5
pass	no	yes	0	0		0	0
	no		-4	-1	90	-4	-1
	yes		2.5	-6	180	3.5	-6
	no		0.5	0	0	0	0.5
pass	no		0	0	0	0	0
fail	yes		-2.5	0	0	-3	0
fail	no						
pass	yes		1	0	0	1.5	
	yes		1	0	0	1	
	yes		2	-3.5	180	2	-3.5
	yes		-1	-4	180	-1	-4
	no		1	0	0	1	
	no		2	-4	180	2	-4
fail	no		1	-1	180	1	-1
pass	no		0	0	0	0	0
pass	no		0.5	0	0	0.5	
pass	yes		0.5	-3	180	0.5	-3
pass	no		0	0	0	0	0
	no		0	0	0	0	0
fail	yes		-6	-4	5	-5	-5.5
pass	no		0	0	0	0	0
pass	no		0	-0.5	60	0	-0.5
	no		0	0	0	0	0
pass	no		0.5	-0.5	180	0.5	-0.5
fail	no		1	0	0	1	-1
pass	no		2	0	0	0	
pass	no		-1	0	0	-1	0
fail	no		2	-2	180	1	-1
	no		0	0	0	0	
	no		0	0	0	0	
	no		0.5	0	0	0.5	
pass	no		0.5	0	0	0.5	
fail	yes		7	0	0	7	0
fail	no		-0.75	0	0	-0.75	
	no		0	-2	180	0	-2
	yes		2.5	0	0	2.5	
	no		0	0	0	0	
pass	no		0	0	0	0	
pass	no		0	0	0	0	
pass	no		0	0	0	0	
fail	no		-1.75	0	0	-1.75	
	yes		0	0	0	0	0
fail	yes		5.5	-2.5	155	5	-2.5
	yes		-0.5	-2	180	-0.5	-2
pass	no		0.75	0	0	0.75	0
fail	no		-0.75	-0.75	180	-0.75	-0.75
fail	no		-5	-1	180	-4.5	0
fail	no		14.5	0	0	13.5	0

	no	0	0	0	0	0
fail	yes	1	0	0	1	0
	yes	0	-2.25	180	1.75	-2.25
	no	1.5	0	0	1.5	0
fail	no	0	0	0	0	0
	yes	-4.5	-3.5	45	-3.5	-4
fail	no	0	0	0	6.5	0
fail	yes	0.5	0	0	0	-1
fail	no	3.5	-0.5	180	3.5	-0.5
	no	0	0	0	0	0
pass	no	0	0	0	0	0
	no	0.5	-0.5	180	0.5	-0.5
	no	10.5	0	0	10.5	0
pass	no	0	0	0	0	0
fail	no	0	0	0	0	0
fail	no	1.5	-1.5	180	1.5	-1.5
fail	no	0	0	0	0	0
	no	0	-2	180	0	-2
	no	0	-1.5	180	0	-1.5
pass	no	0	0	0	0	0
	yes	-6.5	0	0	-5.5	0
	no	0.25	0	0	0.25	0
pass	no	0	-1	180	0	0
fail	no	2	0	0	2	0
fail	no	1	-2.5	180	1	-2.5
fail	no	0.5	0	0	0.5	0
	no	-0.5	0	0	-0.5	0
	no	0.5	0	0	0.5	0
	yes	2	0	0	2	0
	no	0.25	0	0	0	0
pass	no	0.5	-0.75	180	0	0
	yes	0	-0.75	180	0	0
	no	0	-2	180	0	-2
pass	no	1	-2	180	0.5	0
	no	-0.5	0	0	-0.5	0
	no	0.5	0	0	0.5	0
pass	no	0.5	0	0	1	0
pass	no	0	-0.5	180	0	-0.75
	no	1	0	0	1	0
	no	0	0	0	0	0
	yes	2	-1	100	2	-1
pass	no	0.5	0	0	0.5	0
	yes	-9.5	-2	180	-9.5	-2
	yes	0	-2.5	45	0	-2.5
	no	-0.25	0	0	-0.25	0
	yes	-0.5	-0.5	180	-10	-2
	yes	0	-3	180	0	-2
pass	no	1	0	0	4.5	-2
	no	0.25	0	0	0.25	0
	no	-0.75	-0.5	180	-0.75	-0.5

fail	yes		1.25	-1.25	180	0.75	0
	yes		0	-1.5	180	0.5	-4.5
fail	yes		6.5	-2	180	6.5	-2
	no		0.5	-0.75	180	0.5	-0.75
pass	no		0.5	0	0	0.5	0
	yes		3	-1	180	3	0
	no		-0.5	0	0	-0.5	0
	no		3	-1	180	3	-1
pass	no		0	0	0	0	0
	yes		0	0	0	1	0
fail	yes		3	0	0	3	0
	no		0	0	0	0	0
fail	yes		0	-1	90	1	-1.25
	no		5	-4	30	5	-4
	no		0	0	0	0	0
fail	yes		0	0	0	0	0
	no		0	0	0	0	0
	no		-0.5	0	0	-0.5	-0.5
	no		0	0	0	0	-0.5
fail	no		0.25	0	0	-0.25	0
pass	no		0	0	0	0	0
pass	no		0.25	0	0	0.25	0
pass	no		0	0	0	0	0
	no		0	0	0	0	0
	no		0.25	0	0	0.25	0
	no		0	-0.75	90	0	0
	no		4	0	0	4	0
	yes		9.5	0	0	9.5	0
	yes		0	0	0	0	0
pass	no		0	0	0	0	0
	no		-0.75	-0.5	180	-0.75	-0.5
	no		1.5	-2	180	1.5	-2
	yes		3	-2	180	3	-2
	no		0.5	0	0	0.5	0
	no		0	0	0	0	0
	yes		-1.5	0	0	-1.5	0
	yes		0.5	1	180	0.5	1
	no		0	-0.5	180	0	-0.5
pass	no		0.25	-0.75	180	0.5	-0.75
	no		0.25	0	0	0.25	0
	no		0	-1.25	180	0.5	-1.5
	no		0	0	0	0	0
pass	no		0	-0.5	180	0	-0.5
	yes		1	0	0	1	-1
fail	no	yes	2.5	-0.5	15	2.25	-0.25
pass	no		1.25	-0.5	180	1.25	0
pass	no		1	-0.25	180	0.75	0
pass	no		0.25	0		0.25	0
	no		1.25	0		1.25	0
pass	no	yes	5.25	-1.25	10	5.25	-1

pass	no		0.25	0		0.25	-0.25
pass	no		0	0		0	0
pass	no		0.25	-0.25	15	0.25	0
pass	no		0.25	0		0.25	0
fail	no	yes	1.5	-1.75	175	1	-0.75
	no	yes	1.25	0		1.25	0
pass	no		0.5	-0.5	180	0.5	-0.25
	no		0	0		0	0
pass	no		0.25	0		0.25	0
pass	no		0	0		0	0
pass	no		0	0		0	-0.5
pass	no		0	0		-0.25	0
	no	yes	2	0		2	0
pass	no	yes	1	0		1	0
fail	no	yes	0.5	0		0.5	0
pass	no		0	0		0	0
pass	no		0.25	-0.25	180	0	-0.5
pass	no		0.75	0		0.5	-0.25
fail	no		0	0		-0.5	0
fail	no	yes	0.5	-2	180	0.5	-2
fail	no	yes	5	-2.5	165	5.25	-2.5
pass	no		0	0		0	0
fail	no		0	0		0	0
fail	no		0	0		0	0
pass	no		0	0		0	0
pass	no		0	0		0	0
pass	no		0.75	0		0.5	0
pass	no		-0.25	0		-0.25	-0.25
pass	no		0	0		0	0
pass	no		0	0		0	0
fail	no	yes	0.75	-0.5	180	0.5	-1
fail	no	yes	0.75	0		0.5	-0.25
pass	no		-0.25	0		-0.5	0
pass	no		0	0		0	0
pass	no		0	0		0	0
pass	no		0	0		0	0
pass	no		0	0		0	0
	no	yes	4.5	-1.5	180	4.5	-2.5
pass	no		0	0		0	0
	no	yes	-0.5	-1.5	110	-0.75	-3.5
pass	no		0	0		0	-0.25
fail	no	yes	-1.75	0		-0.75	-0.25
pass	no	yes	1	-1	160	1.5	-1.25
	no		0	0		0	0
pass	no		0	0		0	0
pass	no		0.5	0		0.5	0
pass	no	yes	1	-1	75	0	-1.75
pass	yes		0.5	-0.5	75	0.5	-0.5
	no		0	0	0	0	0
pass	no		0.75	0	0	0.75	

pass	no	1.5	-0.25	170	1.5	-0.25
fail	yes	1.25	-0.5	180	1	-0.5
fail	no	-5	-0.25	180	-5	-0.25
	yes	-3	-2	180	-2	-2
pass	no	-12	-3	180	1	
fail	no	5	-2.5	180	5	-3
fail	yes	5	-2.5	170	7.5	-2
pass	no	2	-0.5	180	2.75	-0.5
	no	2.75	-0.25	180	2.75	-0.25
	yes	0	-1	40	0	-1
pass	no	2	0	0	1.25	
fail	no	6.5	-1	180	6.75	-1
fail	no	1.75	-0.25	90	1.75	-0.25
fail	yes	-5.5	-1.5	180	-5	-1.5
fail	no	2.5	0	0	2.5	
fail	no	1.5	0	0	1.5	
	no	2	0	0	2	
fail	yes	-0.5	-1.5	180	-2	-1.75
	yes	2.25	-0.5	180	1.75	-0.5
pass	no	2	0	0	2	
fail	no	0.25	0	0	0.25	
pass	no	2.25	0	0	2.25	
pass	no	1.25	0	0	1.25	
	no	-3.5	-0.5	180	-1.5	-0.5
	no	1.25	0	0	1.25	
	no	0.5	-0.5	180	0.5	-0.5
fail	yes	3	-1	180	3	-1
fail	yes	3	-3.25	180	4.75	-3.25
pass	yes	7	-0.5	180	7.5	-0.75
	no	2.25	-0.5	180	2.25	-0.5
fail	no	2.5	-0.5	180	2.5	-0.5
fail	no	3	-0.75	180	3	-0.75
fail	no	3	-0.5	160	2.5	-0.5
	no	-2	-2	180	-1	-2
fail	yes	2	-0.5	180	2	-0.5
pass	no	3	-0.25	10	3	-0.25
pass	no	2.25	-0.5	90	2.25	-0.5
fail	yes	1.25	0	0	1.25	
fail	no	1	0	0	1	
pass	yes	3.5	-1	180	3.5	-0.5
fail	no	1.75	-0.25	180	1.75	0.25
fail	no	4	-1	180	4	0
	no	3	-0.5	180	3	0.5
pass	no	1.5	0	0	1.25	-0.75
pass	no	0	0	0	0	0
fail	yes	0	-1	180	-11	-3
fail	no	0	0	0	0	0
fail	no	3	0	0	3	0
pass	no	0	0	0	0	0
	no	5.5	0	0	6	0

	no	0	0	0	0	0
pass	no	0	-1	90	0	-1
fail	yes	6	0	0	4.5	0
pass	no	0	0	0	0	0
pass	no	0	0	0	0	0
	no	0	0	0	0	0
fail	yes	-6	-1	180	-7	-1
	no	2.25	-2	180	2	-2
	no	0	0	0	0	0
fail	no	0	0	0	0	0
fail	no	0	0	0	0	0
	no	1	-3	180	1	-2.5
	no	-9.5	-1.5	180	-9.5	-1.5
	no	1	-2	90	1	-2
pass	no	1	-1	180	1	-1
fail	yes	4	-2	180	4	-2
pass	yes	-0.5	-4	180	-1	-2
	no	1.5	0	0	1.5	0
pass	no	0	0	0	0	0
pass	no	0	0	0	0	0
	no	0	0	0	0	0
	no	4	-4	180	4	-4
	no	-4	-1	90	-4	-1
fail	no	-8	-0.5	180	-8	-0.5
	no	0	0	0	0	0
	yes	1	3.5	130	3	2
	no	1	0	0	1	0
fail	no	3	0	0	2	0
pass	no	0	0	0	0	0
	no	0.5	-1	180	0.5	-0.5
	yes	4	0	0	5	0
	yes		0	0	0	
	no	0.75	0	0	0.75	0
	no	1.5	0	0	1.5	0
	no	2	-4	180	2	-4
	yes	1	0	0	1	
fail	yes	6.5	-2	180	6.5	0
pass	no	0.5	0	0	0.5	
	no	1	0	0	1	0
	yes	1	0	0	1	0
pass	no	0.5	0	0	0.5	0
	no	1	0	0	1	0
fail	yes	4	0	0	3	0
	yes	1.25	0	0	1.25	0
	no	1	0	0	1	0
fail	yes	1.25	0	0	1.25	
	no	1.5	0	0	1.5	
pass	no	0	0	0	0	
	no	0	0	0	0	
pass	no	8	0	0	8	0

	no		0	0	0	0	
	no		0	0	0	0	
	no		0	0	0	0	
	yes		2	-4	180	1.5	-2.5
pass	no		0	0	0	0	
pass	no		0	0	0	0	0
pass	no		0	-0.5	180	0	-0.5
pass	no		0	0	0	0	0
fail	no		-0.5	0	0	-0.5	0
	no		0	0	0	0	0
	yes		4.5	-1	180	4.5	-1
	no		0	0		0	0
	no		-8	-2	180	-8	-2
fail	no		3.5	0		5.5	0
pass	no		0	0		0	0
	no		0	0		0	0
pass	no		0	0		0	0
	yes		1.5	-1.5	180	1.5	-1.5
pass	no		0	0		0	0
	no		0	0		0	0
pass	no		0	0		0	0
	no		7	0			
fail	no		0	0		0	0
pass	no		0.5	0		0.5	0
pass	yes	yes	0.75	-0.5	180	0.5	-0.25
pass	yes		1	0		1	0
pass	no		0.5	0		0.5	0
pass	no		0	0		0	0
pass	no		0.75			0.75	
fail	no		0	-1.5	15	0	-1
pass	no		0	0		0	0
fail	no		-2	0		-1.5	0
	no		-1	0		-1	0
fail	no		-0.5	0		-0.75	0
pass	no		0.5	0		0.5	0
fail	yes		1.5	0		-0.5	0
pass	no		0	0		0	0
pass	no		0	0		0	0
	no		2.5	0		3.25	0
	no		0.5	-1.25	180	0.5	-1.25
fail	yes		4	-2.75	170	4.5	-2.75
	no		0	0		0	0
pass	no		1.5	-3	165	2	-2
pass	no		-0.5	0		-0.5	0
pass	no		0	0		0	0
	no		-0.5	0		-0.5	0
pass	no		0	0		0	0
pass	no		0	0		0	0
pass	no		-1	0		-0.75	0
	no		0	0		0	0

pass	no		-0.5	0		-0.5	0
fail	yes		-6.75	-2.25	180	-6.75	-2.75
pass	no		0	0		0	0
fail	no		0	0		0	0
	no		4	0		3.5	0
pass	yes		3.5	-0.25	180	4	-0.75
	no		0	0		0	0
pass	no		0	0		0	0
pass	no		0	0		0	0
pass	no		0.5	0		0.5	0
pass	no		0	0		0	0
	no		-0.75	0		-0.75	0
	no		-0.5	0		-0.5	0
fail	yes	yes	1.5	-1.5	5	2.5	-2.25
pass	no		0	0		0	0
pass	no		0	0		0	0
fail	yes		2	0		2	0
fail	yes		-0.75	0		-0.75	0
pass	no		0	0		0	0
pass	no		0	0		0	0
fail	no	yes	-0.5	-2.75	5	0	-1.5
pass	no		0	0		0	0
	no		0.25	-1.25	180	0.5	-1.25
pass	no		0	0		0	0
	yes		0	0		0	0
pass	no		0	0		0	0
pass	no		0	0		0	0
pass	no		0	0		0	0
	no		-3.5	0		-3.5	0
fail	no		0.5	-2	180	0.5	-2
	yes		2	0	0	2	0
pass	yes		-0.25	0	0	-0.25	0
pass	no		0.5	0	0	0	0.5
	yes		-1.5	-2	180	-1.5	-2
	no		0	-2.5	180	0	-2.5
	no		0.5	0	0	0.5	0
	no		-0.5	0	0	-0.5	0
	no		0.25	0	0	0.25	0
pass	no		0.5	0	0	0.5	0
	yes		0.5	-1	180	0.5	-1
	no		-0.25	0	0	-0.25	0
fail	no		1.25	0	0	1.25	0
	no		1	0	0	1	0
	no		0	0	0	0	0
	no		0.5	0	0	0.5	0
	no		0.5	0	0	0.25	0
fail	no		0	-0.5	90	0	-0.5
fail	yes		1	0	0	1	0
	yes		1	0	0	1	0
	no		0.5	0	0	0.5	0

	no		0	0	0	0	0
	no		-0.75	-0.75	180	-0.75	-0.75
fail	no		0	-0.75	90	0	-0.75
	no		0	-0.5	180	0	-0.5
fail	yes		3	-2	20	2.5	-1
pass	no		0.5	0	0	0.5	0
fail	no		1.5	-1	180	1.5	-1
fail	no		1	0	0	1	0
fail	no		3.75	-2.5	25	4	-2.5
fail	no		0	0	0	0	0
	yes		0	-3	180	0	-1
	yes		0.5	0	0	0.5	0
	no		4	-0.75	20	4	-0.75
	no	yes	6	-2.5	180	6	-2.5
	no		0	-1	90	0	-1
	no		0	-0.75	180	0	-0.75
pass	no		0.5	0	0	0.5	0
pass	no		0	0	0	0	0
pass	no		0.5	0	0	0.5	0
	yes		0.5	-0.5	180	0.5	-0.5
	no		3.5	-4.5	20	3.5	-4.5
	no		0.5	-0.5	180	0.5	-0.5
	yes		2.5	-3	20	1	-2.5
	yes		2	-2	180	2	-2
pass	no		0	0	0	0	0
pass	no		0	0	0	0	0
pass	no		0	0	0	0	0
fail	yes		0	0	0	0	0
fail	yes		6.5	0	0	6.5	0
pass	no		0	0	0	0	0
fail	no		4.75	2	110	6.75	1
fail	yes		-3.25	0.75	180	-6	0.75
pass	no		0	0	0	0	0.5
pass	no		0	0.25	180	0	0.25
fail	yes		0.25	2.25	12	0.25	2.5
fail	no		-1	0.75	90	-1	0.75
fail	yes		4	1.25	70	4	1
	no		3	0	0	0	0
	no		0	0	0	0	0
fail	no		-1.25	0.5	90	-1	0.5
	no		-1	0.75	180	-0.5	0
fail	no		-0.5	0.75	90	-0.75	2.75
fail	no		-0.5	0	0	-0.5	0
pass	no		0	0	0	-0.5	0
fail	no		0.5	0	0	0	0
	yes		3	0	0	4	0.5
fail	no		-5.75	0	0	-6.5	0.5
	no		-20	0.75	90	-16	0.75

	no		-0.5	0	0	-0.75	0
	no		0	0	0	0.75	0
	yes		0.5	0.75	180	0.75	0.5
fail	no		-2	0.5	90	-2	0
fail	no		0	0.5	80	1	0.5
pass	no		-1	0	0	-1	0
fail	no		-2	0	0	-2	0
	no		-17.5	0	0	-10.5	0.75
fail	no		-2	1	55	-0.5	0
fail	no		-1.5	0.25	80	-1	0
pass	no		0	0.5	90	0	0.5
fail	no		-5	2.25	90	-5	3.5
fail	no		-2	0	0	-1.5	0
	no		2	2	70	0	3
fail	no		0	0.25	35	-1	1
	no		0	0	0	0	0
pass	no		-0.25	0	0	0.25	0.75
pass	no		-0.25	0.25	180	-0.75	0.75
fail	no		-1.5	0	0	-1.5	0
	yes		0	0		0	0
fail	no		4.5	-2	180	4.5	-2
	no						
fail	no		0.25	0	0	0.25	0
pass	no		-0.25	0	0	-0.25	0
	no		2	0	0	2	0
pass	no		3	-1	10	1	-0.5
	no		0	0	0	0	0
pass	no		-0.25	0	0	-0.25	0
pass	no		0.5	-0.5	180	0.5	-0.5
pass	no		0.5	0	0	0.5	0
	no		0.5	0	0	0.5	0
	no		0.5	0	0	0.5	0
	no		0.5	-0.5	180	0.5	-0.5
pass	no		0	-0.5	180	0	-0.5
pass	yes		-5	-1	180	-4	0
pass	yes		-3	-3	180	3.5	-3
pass	no		0	0	0	0	0
pass	no		0	0	0	0	0
pass	no		0	0	0	0	0
fail	no		0.5	-1	180	0.5	-1
pass	no		0.5	-1.25	180	0	-1
fail	no		0.75	0	0	0.75	-0.5
	no		0.5	-1	0	0.5	-1
	no	yes	3.75	0	0	3.75	0
	no		0	0	0	0	0
pass	no		0	0	0	0	0
	no		1	-2.75	180	1	-2.75
	yes		4	0	0	4	0
pass	no		0	0	0	0	0
pass	no		-1.75	0	0	-1.75	0

	no		0	0	0	0	0
fail	no		0.5	0	0	0.5	0
pass	no		0	0	0	0	0
	no		0.5	0	0	0.5	0
	no		0	0	0	0	0
pass	no		0.5	0	0	0.5	0
pass	no		0	0	0	0	0
	no		5.5	-2	180	5.5	-1
fail	no		2.5	-2.5	180	2.5	-1.5
pass	no		7.25	-0.75	165	6	-1
	no		4	-1.5	10	3	-1
fail	no		-0.75	0	0	-0.75	0
pass	no		0	0	0	0	0
	yes		2	-3	180	2	-2
pass	no		0	0	0	0	0
	yes		-13	-3	180	-13.5	-2.5
fail	no		0	0	0	0	0
pass	no		0.5	0	0	0.5	0
fail	no		0	0	0	0	0
fail	no		1	0	0	1	0
fail	no		-2	0	0	-2	0
	no		0	0	0	0	0
	no		0	0	0	0	0
pass	no		0.5	0	0	0.5	0
fail	no		0.5	-3	180	0.5	-3
	no		1	0	0	1	0
fail	no		0	-3	180	0	0
	no		0	0	0	0	0
pass	no		0	0	0	0	0
	yes		0	0	0	0	0
	no		0.5	0	0	0.5	0
pass	no		0	0	0	0	0
	no		0	0	0	0	0
	no		0	0	0	0	0
	no		0	-1.25	90	0	-1.25
	no	yes	3.5	0	0	4	0
fail	no		-1	0	0	-1	0
	no		0	-1	180	0	-1
pass	no		0	0	0	0	0
fail	no		0	0	0	0	0
	no		-0.5	0	0	-0.5	0
pass	no		0	0	0	0	0
	no		0	0	0	0	0
	no		0	-1.5	90	0	-1.5
fail	yes		1.5	-1.75	180	0.25	-1.5
	no	yes	4	-4	20	2	-2
	no		-1	0	0	-1	0
fail	no	yes	2	0	0	2	0
pass	no		1	0	0	1	0
pass	no		-0.25	0	0	-0.25	0

	no		-0.25	0	0	-0.25	0
	no		1	0	0	1	0
fail	no	yes	3.5	-4	180	3.5	-4
fail	no		1	0	0	1	0
fail	no		-0.5	0	0	-0.5	0

Laxis	Requiv sphere	Lequiv sphere	Astigmatism	Least ametropic equiv sphere	Rx group	Accomm	Visual field	Pathology
180	-0.25	-0.25	no	0	emmetropi	accurate	full	
180	-0.75	-0.75	yes	0	myopia	accurate	full	
	0	0	no	0	emmetropia		full	
180	-1.25	-0.75	no	-0.75	myopia	accurate	full	
20	2.5	3	yes	2.5	hypermetr	accurate		
180	0.75	0.75	no	0.75	emmetropia			
180	2.75	2.75	no	2.75	hypermetr	accurate		
	0	0	no	0	emmetropia			
	0		no	0	emmetropia			
5	-1	-1	yes	0	myopia	under	full	
	0.5	0.5	no	0.5	emmetropi	accurate	full	
	1	1	no	1	emmetropi	accurate	full	
	0.75	0.75	no	0.75	emmetropi	accurate	full	
	-2.25	-4.5	no	-2.25	myopia	accurate	full	
	3	3	no	3	hypermetropia			
180	0.75	0.75	yes	0.75	emmetropi	under	full	
	0	0	no	0	emmetropia		full	
	0.5	0.5	no	0.5	emmetropia			
	0	0	no	0	emmetropia			
	0		no	0	emmetropia			
	0	0	no	0	emmetropia		Left Hemi	hemianopia
	1	1	no	1	emmetropia			
180	-2	-2	yes	-2	myopia			
180	-3.5	-1.5	yes	-1.5	myopia			
	-30	-30	no	-30	myopia			
90	-3	-2	yes	-2	myopia		full	
90	-1.75	-3	yes	-1.75	myopia	accurate	full	
	1	1	no	1	emmetropia			
90	0.5	0.5	yes	0.5	emmetropia			
180	0	0	yes	0	emmetropi	accurate	Full R+L	
	4.5	3	no	3	hypermetropia		full R+L	
							Full R+L	
	1	1	no	1	emmetropia			
170	-0.75	-0.75	yes	-0.75	myopia			
	-5	-5	no	-5	myopia	accurate		
	3	3	no	3	hypermetr	accurate		
	5	5	no	5	hypermetropia		Full R+L	
180	1.5	1.5	yes	1.5	emmetropi	accurate	Full R+L	
	6	5	yes	5	hypermetr	accurate	sup/inf def field defect	
						accurate	Full R+L	
						accurate	Full BEO	
	2	2	no	2	hypermetr	accurate	Full BEO	
180	-1	-1	yes	-1	myopia			
180	3.13	3.13	no	3.13	hypermetropia		Full BEO	
180	0.25	0.25	no	0.25	emmetropi	accurate		
	-0.5	-0.5	no	-0.5	myopia	accurate	Full BEO	

	0.75	0.75	no	0.75	emmetropi	accurate	Full BEO	
	5		yes	5	hypermetropia		Full R	
						accurate	Full BEO	
						accurate	Full BEO	
						accurate	Full BEO	
	1.25	1.25	no	1.25	emmetropi	accurate	Full BEO	
180	1	1	yes	1	emmetropi	accurate	Full BEO	
7	0.25	1.25	yes	0.25	emmetropi	accurate	Full BEO	
175	-6.5	-6	yes	-6	myopia	accurate	Full BEO	
						accurate	Full BEO	
	-2.5	-1.5	no	-1.5	myopia	accurate	?gen restri	field defect
15	1.5	1.5	yes	1.5	emmetropi	accurate	full BEO	
180	0.5	0.5	yes	0.5	emmetropi	accurate	Full BEO	
	0.75	0.75	no	0.75	emmetropi	accurate	R full	
180	0.5	0.5	yes	0.5	emmetropi	accurate	Full BEO	
180	-0.5	-0.5	yes	-0.5	myopia		full BEO	
180	-0.25	-0.25	no	-0.25	emmetropia			
180	-0.25	-0.25	no	-0.25	emmetropi	accurate		
						accurate	Full BEO	
180	3.25	3.25	yes	3.25	hypermetr	accurate	Full BEO	
10	1.75	2.5	yes	1.75	hypermetropia		Full BEO	
180	-0.38	-0.38	no	-0.38	emmetropia			
	0.5	0.5	no	0.5	emmetropia			
90	0.25	0.25	no	0.25	emmetropia			
180	-0.5	-1	yes	-0.5	myopia	accurate		
	0.5	0.5	no	0.5	emmetropia		Full BEO	
180	-1	-1.75	yes	-1	myopia	accurate	Full BEO	
90	0	-0.5	yes	0	myopia			
180	-0.25	-0.25	no	-0.25	emmetropi	accurate	Full BEO	
180	-1	-1	yes	-1	myopia			
180	2	-1.5	yes	-1.5	myopia			
						accurate	Full BEO	
	2.5	2.5	no	2.5	hypermetr	under	Left hemi	
						accurate		
	-1	-0.5	yes	-0.5	myopia			
90	1.5	1.5	yes	1.5	emmetropia			
180	1.63	2.38	no	1.63	hypermetr	under	Full BEO	
	1.5	1.5	no	1.5	emmetropia			
	-3	-0.5	yes	-0.5	myopia		full BEO	
							full BEO	
	-0.5	0	yes	0	myopia	accurate	full BEO	
180	-17	-17	yes	-17	myopia	accurate	Full BEO	CD ratio 0.8
20	3.25	3.25	yes	3.25	hypermetr	under	?full R+L	
	0	2	no	0	hypermetr	accurate		
170	0.5	0.5	yes	0.5	emmetropi	under		
	2	2	no	2	hypermetr	accurate	full BEO	
	0	0	no	0	emmetropi	accurate	Full BEO	
	0	0	no	0	emmetropi	accurate	Full BEO	
	0	0	no	0	emmetropi	accurate	Full BEO	

	1	1	no	1	emmetropi	accurate		
90	0.25	0.25	no	0.25	emmetropi	accurate	Full BEO	
	0.5	0.5	no	0.5	emmetropi	accurate	? Full BEO	
	1.5	1.5	no	1.5	emmetropi	under		
180	2	2.5	yes	2	hypermetr	under	Full BEO	
180	1.5	1.5	yes	1.5	emmetropi	accurate	Full BEO	
10	0	-0.25	yes	0	emmetropia		Full BEO	
180	1.75	2.25	no	1.75	hypermetr	accurate	Full BEO	
	0	0	no	0	emmetropi	accurate	Full BEO	
	0	0	no	0	emmetropi	accurate	Full BEO	
	0.75	0.75	no	0.75	emmetropi	accurate	Full BEO	
	0	0	no	0	emmetropi	accurate		
	0	0	no	0	emmetropi	accurate	Full BEO	
	0.75	0.75	no	0.75	emmetropi	under	Full BEO	
	1	1	no	1	emmetropi	under	Full BEO	
170	-11	-13.5	yes	-11	myopia	accurate	Full BEO	
	0	0	no	0	emmetropi	accurate	Full BEO	
90	-15	-13.5	yes	-13.5	myopia			
	1.75	1.5	yes	1.5	emmetropia			
180	0.13	0.13	no	0.13	emmetropi	accurate	Full BEO	
175	-1.5	-1.5	yes	-1.5	myopia	accurate	Full BEO ??	
	1	1	no	1	emmetropi	accurate		
	3.5	3.5	no	3.5	hypermetr	under	Full BEO ??	
180	-18.75	-19.25	yes	-18.75	myopia		Full BEO ??	
20	2	1.63	yes	1.63	hypermetr	accurate	Full BEO	
	4	3.75	yes	3.75	hypermetr	accurate	Full BEO	
	0.5	0.5	no	0.5	emmetropi	accurate	Full BEO	
	-0.5	-0.5	no	-0.5	myopia	accurate	Full BEO	
	0	0	no	0	emmetropi	accurate	Full BEO	
	0.5	0.5	no	0.5	emmetropi	accurate	Full BEO	
	0	0	no	0	emmetropi	accurate	Full BEO	
	0.5	0.5	no	0.5	emmetropi	accurate	Full BEO	
	1.25	0.5	no	0.5	emmetropi	accurate	Full BEO	
165	1.5	1	yes	1	emmetropia			pale disc
180	0.25	0.25	no	0.25	emmetropi	accurate	Full BEO	
	0.75	0.75	no	0.75	emmetropi	accurate	Full BEO	
	-1.5	0	yes	0	myopia	over	Full BEO	
10	0.5	0.5	yes	0.5	emmetropia			pale disc
	0	0	no	0	emmetropi	accurate	Full BEO	
	1	1	no	1	emmetropi	accurate	Full BEO	
	-18	-0.5	yes	-0.5	myopia	accurate	Full BEO	myopic disc
	0	0	no	0	emmetropi	accurate	Full BEO	
		0	no	0	emmetropi	accurate	Full BEO	
180	2.5	2.25	yes	2.25	hypermetr	under	Full BEO	cataract, min
20	-0.75	-0.75	yes	-0.75	myopia	accurate	Full BEO	CD ratio 0.5
	0	0	no	0	emmetropi	accurate	Full BEO	
90	2.75	0.38	yes	0.38	hypermetr	accurate	Full BEO	
	1	1	no	1	emmetropi	accurate	Full BEO	
180	-0.25	-0.25	no	-0.25	emmetropi	accurate	Full BEO	
	-13.5	-13.5	no	-13.5	myopia	accurate	Full BEO	

10	-0.25	1	yes	-0.25	emmetropi	accurate	field defect	field defect
	0	0	no	0	emmetropi	accurate	Full BEO	
	0	0	no	0	emmetropi	accurate	Full BEO	
	-3	-1	no	-1	myopia	accurate	Full BEO	
180	0	0	yes	0	emmetropi	accurate	Full BEO	
	0	0	no	0	emmetropi	under	Full BEO	
	4	4	no	4	hypermetr	inconclusiv	Full BEO	
105	1.13	1.13	yes	1.13	emmetropi	accurate	Full BEO	CD ratio 0.7
180	-2.5	-8	yes	-2.5	myopia	accurate	Full BEO	
	0.5	0.5	no	0.5	emmetropi	over	Full BEO	
	0.5	0.5	no	0.5	emmetropi	accurate	Full BEO	
99	0.13	0.13	no	0.13	emmetropi	accurate	Full BEO	
	0	0	no	0	emmetropi	accurate	Full ?red at	field defect
180	0.5	0.5	yes	0.5	emmetropi	accurate	Full BEO	
	1	1	no	1	emmetropi	accurate	Full BEO	
	0	0	no	0	emmetropi	accurate	Full BEO	
180	5.75	5.75	yes	5.75	hypermetr	inconclusiv	NP	blepharitis
	-1	-1	no	-1	myopia	accurate	Full BEO	
	0	0	no	0	emmetropi	accurate	Full BEO	
	0.5	0.5	no	0.5	emmetropi	accurate	Full BEO	CD ratio 0.7
180	-2.5	-2.5	yes	-2.5	myopia	under	Full BEO	
	-13.88	-18	yes	-13.88	myopia	inconclusiv	Full BEO	
	0.5	0.5	no	0.5	emmetropi	accurate	?red inf?	field defect
180	2.25	2.25	no	2.25	hypermetr	under	Full BEO	
	0	0	no	0	emmetropi	accurate	Full BEO	
180	0.5	0.5	yes	0.5	emmetropi	accurate	full BEO	
180	0.5	0.5	yes	0.5	emmetropi	inconclusiv	inconclusive	
180	-0.5	-0.5	yes	-0.5	myopia	under	Full BEO	CD ratio 0.5
	1	1	no	1	emmetropi	accurate	full BEO	
	0.5	0.5	no	0.5	emmetropi	accurate	full BEO	
	0	0	no	0	emmetropi	accurate	Full BEO	
	0.75	0.75	no	0.75	emmetropi	accurate	Full BEO	
120	2.63	3.63	no	2.63	hypermetr	accurate	Full BEO	
	0	0	no	0	emmetropi	accurate	Full BEO	
180	0	0	yes	0	emmetropi	inconclusiv	no respons	CD ratio 0.9 pall
	0.5	0.5	no	0.5	emmetropi	accurate	Full BEO	
180	-2.38	-4	yes	-2.38	myopia	accurate	Full BEO	
	0.5	0.5	no	0.5	emmetropi	accurate	Full BEO	
	-1	-1	no	-1	myopia	accurate	Full BEO	
	0.75	0.75	no	0.75	emmetropi	accurate	Full BEO	
180	1.5	2.25	yes	1.5	hypermetr	accurate	Full BEO	
	0.5	0.5	no	0.5	emmetropi	accurate	Full BEO	
	0.5	0.5	no	0.5	emmetropi	accurate	Full BEO	
110	-0.5	-0.38	yes	-0.38	myopia	accurate	Full BEO	
	0	0	no	0	emmetropi	accurate	Full BEO	
180	0.13	0.13	no	0.13	emmetropi	accurate	Full BEO	
180	0.5	0.5	yes	0.5	emmetropi	under	inf defect	feild defect
180	-0.38	-0.38	no	-0.38	emmetropi	accurate	Full BEO	
	0.5	0.5	no	0.5	emmetropi	accurate	Full BEO	
175	-1.25	-3.25	yes	-1.25	myopia	accurate	Lhemianop	hemianopia

180	0	0	yes	0	emmetropi	over	Full BEO	
180	1	1	yes	1	emmetropi	over	Full BEO	
160	1.75	1.25	yes	1.25	emmetropi	under	Full BEO	
180	0.25	0.25	yes	0.25	emmetropi	under	Full BEO	
	0.5	0.5	no	0.5	emmetropi	accurate	Full BEO	
180	0.5	0.5	yes	0.5	emmetropi	accurate	Full BEO	
180	-0.38	-1.25	yes	-0.38	myopia	accurate	Full BEO	
165	-7.25	-7.25	yes	-7.25	myopia	accurate	Full BEO	
3	1	0.5	yes	0.5	emmetropi	under	Full BEO	
	0	0	no	0	emmetropi	under	Full BEO	
5	2.25	2.25	yes	2.25	hypermetr	accurate	Full BEO	
170	-8.75	-7.5	yes	-7.5	myopia	accurate	Full BEO	
	0.75	0.75	no	0.75	emmetropi	accurate	full BEO	
	1	1	no	1	emmetropi	accurate	Full BEO	
180	6.5	5	yes	5	hypermetr	under	full BEO	
10	-13.25	-10.88	yes	-10.88	myopia	accurate	Full BEO	CD ratio 0.5
	0	0	no	0	emmetropi	accurate	Full BEO	
	0.5	0.5	no	0.5	emmetropi	accurate	Full BEO	
180	0	0	yes	0	emmetropi	accurate	Full BEO	
	0.5	0.5	no	0.5	emmetropi	accurate	Full BEO	
30	-11.5	-12	yes	-11.5	myopia	accurate	inconclusive	
180	-3.5	-1.5	yes	-1.5	myopia	accurate	Full BEO	
	0	0	no	0	emmetropi	under	inconclusive	
	0	0	no	0	emmetropi	accurate	Full BEO	
0	-0.5	-0.5	no	-0.5	myopia		full	
0	0.25	0.25	no	0.25	emmetropia			
0	0	0	no	0	emmetropi	accurate	full	
0	0.25	0.5	no	0.25	emmetropi	under	full	
0	0	0	no	0	emmetropia		full	
180	-1.25	-0.75	yes	-0.75	myopia			
180	1.25	1.25	no	1.25	emmetropia			
0	0	0	no	0	emmetropia			
0	0	0	no	0	emmetropia			
180	-0.38	-0.38	no	-0.38	emmetropi	accurate	full	
0	0.25	0	yes	0	emmetropia			
0	1.5	1.5	no	1.5	emmetropia			
0	0	0	no	0	emmetropi	accurate		
0	0	0	no	0	emmetropia			
180	0.25	-0.13	no	-0.13	emmetropia			
0	0	0	no	0	emmetropia			
180	-4	-4.25	yes	-4	myopia			none
180	-1	-2	yes	-1	myopia			
180	-2.25	-2.25	yes	-2.25	myopia	accurate		
								keratoconus
180	0	2.25	no	0	hypermetr	accurate		
0	0.5	0.5	no	0.5	emmetropi	accurate	Full	
0	0.5	0.5	no	0.5	emmetropia		full	
	0.5	0.5	no	0.5	emmetropi	accurate	full	
180	0	0	yes	0	emmetropia		Full	
0	0.5	0.5	no	0.5	emmetropia			

	0.5	0.5	no	0.5	emmetropia			
180	2.5	2.5	yes	2.5	hypermetropia		full	
0	0	0	no	0	emmetropia			
180	-11.5	-11.5	yes	-11.5	myopia			
180	-7	-7	yes	-7	myopia	accurate		
180	-0.75	-0.75	no	-0.75	myopia	accurate	full	
0	0.25	0.25	no	0.25	emmetropi	accurate		
						inconclusive		cataracts R&L
	1	1	no	1	emmetropi	inconclusive		
180	-0.5	-0.5	yes	-0.5	myopia	over		
90	-1.5	-1.5	yes	-1.5	myopia	accurate	generally r	retinal degenera
180	2.25	2.25	yes	2.25	hypermetr	over	full	
180	0.5	0.5	yes	0.5	emmetropi	under	generally r	nystagmus
	0	0	no	0	emmetropi	accurate	full	
	0	0.75	no	0	emmetropi	accurate	full	
	2.5	2.5	no	2.5	hypermetr	accurate		
180	1	1	yes	1	emmetropi	accurate		
	0.5	0.5	no	0.5	emmetropi	accurate	full	
	1	1	no	1	emmetropi	accurate		
	5	6	no	5	hypermetr	inconclusive		
	1	1	no	1	emmetropi	accurate		
	4	4	no	4	hypermetr	inconclusiv	full	
	0.5	0.5	no	0.5	emmetropi	accurate		
	3.5	3.5	no	3.5	hypermetr	accurate	full	
180	-3.5	0.5	yes	0.5	myopia	under	full	
	0.5	0.5	no	0.5	emmetropi	accurate	full	
180	0	0	yes	0	emmetropi	accurate	very restric	retinal degenera
	2	2	no	2	hypermetr	accurate	full	
	1	1	no	1	emmetropi	accurate		
	0	0	no	0	emmetropi	accurate	full	
	0.5	0.5	no	0.5	emmetropi	accurate	full	
	-4	-4	no	-4	myopia	accurate	full	
	0.5	0.5	no	0.5	emmetropi	accurate	full	
180	4	4	yes	4	hypermetr	under		nystagmus
180	0.75	0.75	yes	0.75	emmetropi	accurate	full	
	0	0	no	0	emmetropi	inconclusiv	full	entropion/conju
	0.5	0.5	no	0.5	emmetropi	accurate	full	
	0.5	0.5	no	0.5	emmetropi	accurate	full	
180	-10.75	-10.75	yes	-10.75	myopia	inconclusiv	generally r	field defect
	0	-0.5	no	0	myopia	accurate	full	
	0.5	0.5	no	0.5	emmetropi	inconclusive		nystagmus
180	-2.25	0.75	yes	-2.25	myopia	accurate	full	retinal laser scar
	0	0	no	0	emmetropi	accurate	full	
	-2.5	-2.5	no	-2.5	myopia	accurate	full	
135	6	6	yes	6	hypermetr	inconclusiv	full	
35	-1.75	-3	yes	-1.75	myopia	under	inconclusiv	nystagmus
	0	0	no	0	emmetropi	accurate	full	cataracts
175	-1.38	-0.75	yes	-0.75	myopia	accurate	full	
	0.5	0.5	no	0.5	emmetropi	inconclusiv	very restric	coloboma, large
	0	0	no	0	emmetropi	accurate	full	

100	-0.25	-0.25	yes	-0.25	emmetropi	accurate	full	proptosis
5	6.25	6	yes	6	hypermetr	accurate	full	
	0	0	no	0	emmetropi	accurate	inconclusive	
	0.5	0.5	no	0.5	emmetropi	accurate	full	
175	4	4	yes	4	hypermetr	accurate	full	
	3	3	no	3	hypermetr	inconclusiv	inconclusive	
180	0.13	0.13	no	0.13	emmetropi	accurate	inconclusive	
180	0.5	0.5	yes	0.5	emmetropi	accurate	full	
90	0	0.5	yes	0	emmetropi	accurate	full	
	0	0	no	0	emmetropi	accurate	full	
	0	0	no	0	emmetropi	accurate	full	cataract/ disc irr
180	3	2.5	yes	2.5	hypermetr	accurate	full	
180	-2	-2.25	yes	-2	myopia	accurate	full	
5	-3	-3.5	yes	-3	myopia	accurate	full	
180	6.5	6.5	yes	6.5	hypermetr	accurate	full	
	1	1	no	1	emmetropi	accurate	full	
	0	0	no	0	emmetropi	accurate	full	
180	-1	-1	yes	-1	myopia	inconclusiv	full	
155	-2	-2	yes	-2	myopia	accurate	full	
	0.5	0.5	no	0.5	emmetropi	accurate	full	
	-2.5	-2.5	no	-2.5	myopia	accurate	full	
15	-5.25	-2.25	no	-2.25	myopia	accurate	full	ocular albinism
	0	0	no	0	emmetropi	accurate	full	
	1	4	no	1	hypermetr	inconclusiv	full	
	0	0	no	0	emmetropi	accurate	full	
	0.5	0	no	0	emmetropi	accurate	full	
172	-2	-2.13	yes	-2	myopia	accurate	full	
	2.5	2.5	no	2.5	hypermetr	accurate	full	
	0	0	no	0	emmetropi	accurate	full	
180	0.75	0.75	yes	0.75	emmetropi	inconclusiv	full	
	0	0	no	0	emmetropi	inconclusive		
	0	0	no	0	emmetropi	accurate	full	
	0.5	0.5	no	0.5	emmetropi	accurate	full	
160	-10.38	-10.38	yes	-10.38	myopia	under	full	myopic discs & f
180	-1.25	-1.25	yes	-1.25	myopia	accurate	full	
	1	2	no	1	hypermetr	inconclusive		
	0	0	no	0	emmetropi	accurate	full	
	1.75	1.75	no	1.75	emmetropi	under	full	
	0	0	no	0	emmetropi	accurate	full	
	0	0	no	0	emmetropi	accurate		
180	-1.75	-1	yes	-1.75	myopia	inconclusive		
	2	2	no	2	hypermetr	under	full	
145	-1.75	-3.5	yes	-1.75	myopia	inconclusiv	inconclusive	
180	0	-0.5	yes	0	myopia	accurate	Full BEO	
	4.5	4.5	no	4.5	hypermetr	inconclusiv	FullBEO	
	0	0	no	0	emmetropi	accurate	Full BEO	
	0.5	0.5	no	0.5	emmetropi	accurate	FullBEO	
	0	0	no	0	emmetropi	inconclusiv	inconclusive	
180	-1	-1.5	yes	-1	myopia	inconclusiv	inconclusive	
180	1.75	1.75	yes	1.75	emmetropi	accurate	inconclusiv	chalazion

	4	4	no	4	hypermetr	inconclusiv	inconclusive	
	1.5	1.5	no	1.5	emmetropi	accurate	full BEO	
	0	0	no	0	emmetropi	accurate	Full BEO	
	0	0	no	0	emmetropi	accurate	full BEO	
	0.5	0.5	no	0.5	emmetropi	accurate	inconclusive	
	2.5	2.5	no	2.5	hypermetr	under	Full BEO	cataracts
180	-6.25	-5.25	yes	-5.25	myopia	inconclusiv	FullBEO	nystagmus
	0	0	no	0	emmetropi	accurate	Full BEO	
	0.5	0.5	no	0.5	emmetropi	accurate	FullBEO	
	0	0	no	0	emmetropi	accurate	Full BEO	
	1	1	no	1	emmetropi	accurate	Full BEO	
	-6	-6	no	-6	myopia	inconclusiv	inconclusiv	ptosis R&L
180	-2.13	-1.25	yes	-1.25	myopia	accurate	Full BEO	
	0	0	no	0	emmetropi	accurate	inconclusive	
	0	0	no	0	emmetropi	accurate	Full BEO	
	0	0	no	0	emmetropi	accurate	Full BEO	
	0.5	0.5	no	0.5	emmetropi	inconclusiv	Full BEO	
	-1	-1	no	-1	myopia	accurate	Full BEO	lids, hayfever, m
	0.5	0.5	no	0.5	emmetropi	accurate	Full BEO	
	1	1	no	1	emmetropi	inconclusiv	inconclusive	
	0.5	0.5	no	0.5	emmetropi	accurate	Full BEO	
	0	0	no	0	emmetropi	accurate	Full BEO	
	0	0	no	0	emmetropi	accurate	Full BEO	
	1.5	1.5	no	1.5	emmetropi	accurate	Full EE	blepharitis, mild
	1	1	no	1	emmetropi	accurate	Full BEO	
180	1	1	yes	1	emmetropi	inconclusiv	inconclusiv	nystagmus
180	0.63	-1.38	no	0.63	myopia	accurate	Fulll BEO	
	1	1	no	1	emmetropi	under	Full BEO	
90	0	0	yes	0	emmetropi	accurate	inconclusive	
	0	0	no	0	emmetropi	accurate	Full BEO	
	-1	-1	no	-1	myopia	accurate	Full BEO	
90	0.88	0.88	yes	0.88	emmetropi	accurate	inconclusive	
	0.5	0.5	no	0.5	emmetropi	accurate	Full BEO	
	-1	-1	no	-1	myopia	accurate	Full BEO	
180	-3.25	-3.25	yes	-3.25	myopia	accurate	inconclusive	
	1	1	no	1	emmetropi	accurate	Full BEO	
	-0.5	-0.5	no	-0.5	myopia	accurate	Full BEO	
175	-8.5	-4.5	yes	-4.5	myopia	accurate	Full BEO	
180	0.75	0.75	no	0.75	emmetropi	accurate	inconclusive	
180	-0.5	-0.5	yes	-0.5	myopia	accurate	Full BEO	
	-1	-1	no	-1	myopia	accurate	Full BEO	
180	1.25	1.25	yes	1.25	emmetropi	accurate	Full BEO	
	0.75	0.75	no	0.75	emmetropi	accurate	Full BEO	
	3	3	no	3	hypermetr	under	inconclusive	
	3	3	no	3	hypermetr	inconclusiv	inconclusive	
	0.5	0.5	no	0.5	emmetropi	accurate	Full BEO	
	0.5	0.5	no	0.5	emmetropi	accurate	inconclusive	
	0	0	no	0	emmetropi	accurate	Full BEO	
180	5.13	4.13	no	4.13	hypermetr	inconclusiv	inconclusive	
180	0.63	0.63	no	0.63	emmetropi	accurate	Full BEO	conjunctivitis, al

	0.5	0.5	no	0.5	emmetropi	accurate	Full BEO	
180	-8.25	-8.25	no	-8.25	myopia	inconclusiv	?inconclusive	
	0	0	no	0	emmetropi	accurate	Full BEO	
90	-4.5	-4.5	yes	-4.5	myopia	inconclusiv	inconclusive	
180	-0.5	0.5	yes	-0.5	myopia	under	Inconclusive	
0	0.5	0.25	no	0.25	emmetropi	accurate	Inconclusive	
	0	0	no	0	emmetropi	accurate	Full BEO	
	-2.5	-3	no	-2.5	myopia	accurate	Full BEO	
								conjunctivitis
	1	1.5	no	1	emmetropi	accurate	Full BEO	
	1	1	no	1	emmetropia			L cataract
180	0.25	0.25	yes	0.25	emmetropia		Full BEO	
180	-3	-3	yes	-3	myopia	under		
	1	1	no	1	emmetropi	under		
180	0	0	yes	0	emmetropia			
180	0.5	0.5	yes	0.5	emmetropi	under	RHS reduce	retinal holes
	0	0	no	0	emmetropi	accurate	Full BEO	
	0.5	0.5	no	0.5	emmetropi	accurate	Full BEO	
180	-1	-1	yes	-1	myopia		Full BEO	
	0	0	no	0	emmetropi	under	Full BEO	
	0	0	no	0	emmetropi	accurate	Inconclusive	
12	-8	-7.75	yes	-7.75	myopia	accurate		Cataract/RD
	0	0	no	0	emmetropi	accurate	Full BEO	
90	-0.25	-0.25	no	-0.25	emmetropia			
	0	0	no	0	emmetropi	accurate	Inconclusive	
180	0.25	0.25	no	0.25	emmetropi	accurate	Full EE	
180	1	0.5	yes	0.5	emmetropi	accurate	full BEO	
	2	0	no	0	hypermetr	accurate	Full LE, R lt	field defect
	-1	-1	no	-1	myopia	accurate	Full BEO	
180	1	0.5	yes	0.5	emmetropi	under	RHS attn re	field defect
	0	0	no	0	emmetropi	accurate	inconclusve	
	0	0	no	0	emmetropi	accurate	Inconclusive	
	0.5	0.5	no	0.5	emmetropi	accurate	Full BEO	
	0.5	0.5	no	0.5	emmetropi	accurate	Full BEO	
	7	7	no	7	hypermetr	under	R total hen	Cataract/hemi
	-0.75	-0.75	no	-0.75	myopia	inconclusive		
180	-1	-1	yes	-1	myopia	inconclusiv	full BEO	Keratoconus
	2.5	2.5	no	2.5	hypermetr	under	Full BEO	
	0	0	no	0	emmetropi	accurate	inconclusive	
	0	0	no	0	emmetropi	accurate	Full BEO	
	0	0	no	0	emmetropi	accurate	Full BEO	
	0	0	no	0	emmetropi	accurate	Full BEO	
	-1.75	-1.75	no	-1.75	myopia	under	R hemiano	hemianopia
	0	0	no	0	emmetropi	accurate		
135	4.25	3.75	yes	3.75	hypermetr	accurate	full	
180	-1.5	-1.5	yes	-1.5	myopia	accurate		
0	0.75	0.75	no	0.75	emmetropi	accurate	full	
180	-1.13	-1.13	no	-1.13	myopia			
0	-5.5	-4.5	yes	-4.5	myopia			
0	14.5	13.5	no	13.5	hypermetropia			

0	0	0	no	0	emmetropia			
0	1	1	no	1	emmetropi	accurate	full	
180	-1.13	0.63	yes	0.63	myopia	accurate		
0	1.5	1.5	no	1.5	emmetropia		full	
0	0	0	no	0	emmetropi	accurate		
120	-6.25	-5.5	yes	-5.5	myopia			
0	0	6.5	no	0	hypermetropia			
90	0.5	-0.5	yes	0.5	myopia	accurate		
180	3.25	3.25	no	3.25	hypermetr	accurate		
0	0	0	no	0	emmetropi	accurate		
0	0	0	no	0	emmetropi	accurate		
180	0.25	0.25	no	0.25	emmetropi	accurate		
0	10.5	10.5	no	10.5	hypermetropia			
0	0	0	no	0	emmetropi	accurate	full	
0	0	0	no	0	emmetropi	accurate	full	
20	0.75	0.75	yes	0.75	emmetropi	under	full	
0	0	0	no	0	emmetropi	accurate	full	
180	-1	-1	yes	-1	myopia	accurate	full	
180	-0.75	-0.75	yes	-0.75	myopia	accurate	full	
0	0	0	no	0	emmetropi	accurate	full	
0	-6.5	-5.5	no	-5.5	myopia			
0	0.25	0.25	no	0.25	emmetropia			
0	-0.5	0	yes	0	myopia			
0	2	2	no	2	hypermetr	accurate		
180	-0.25	-0.25	yes	-0.25	emmetropia			
0	0.5	0.5	no	0.5	emmetropi	accurate	full	
0	-0.5	-0.5	no	-0.5	myopia	accurate	full	
0	0.5	0.5	no	0.5	emmetropia			
0	2	2	no	2	hypermetropia			
0	0.25	0	no	0	emmetropi	accurate		
0	0.13	0	no	0	emmetropi	accurate	full	
0	-0.38	0	no	-0.38	emmetropia		R Hemi	hemianopia
180	-1	-1	yes	-1	myopia			
0	0	0.5	yes	0	emmetropi	accurate	full	
0	-0.5	-0.5	no	-0.5	myopia		full	
0	0.5	0.5	no	0.5	emmetropia			
0	0.5	1	no	0.5	emmetropia			
180	-0.25	-0.38	no	-0.25	emmetropia			
0	1	1	no	1	emmetropi	accurate		
0	0	0	no	0	emmetropi	accurate		
95	1.5	1.5	yes	1.5	emmetropi	accurate	full	
0	0.5	0.5	no	0.5	emmetropi	accurate	full	
180	-10.5	-10.5	yes	-10.5	myopia			
180	-1.25	-1.25	yes	-1.25	myopia			
0	-0.25	-0.25	no	-0.25	emmetropi	accurate		
180	-0.75	-11	yes	-0.75	myopia			
180	-1.5	-1	yes	-1	myopia		right hemi	hemianopia
180	1	3.5	yes	1	hypermetr	accurate	full	
0	0.25	0.25	no	0.25	emmetropia		full	
180	-1	-1	no	-1	myopia		full	

0	0.63	0.75	yes	0.63	emmetropi	accurate		
170	-0.75	-1.75	yes	-0.75	myopia			
180	5.5	5.5	yes	5.5	hypermetr	accurate		
180	0.13	0.13	no	0.13	emmetropia			
0	0.5	0.5	no	0.5	emmetropi	accurate		
0	2.5	3	yes	2.5	hypermetropia			
0	-0.5	-0.5	no	-0.5	myopia			
180	2.5	2.5	yes	2.5	hypermetropia			
	0	0	no	0	emmetropi	accurate	full	
0	0	1	no	0	emmetropi	accurate	maybe left	hemianopia
0	3	3	no	3	hypermetr	accurate		
0	0	0	no	0	emmetropia			
80	-0.5	0.38	yes	0.38	myopia			
180	3	3	yes	3	hypermetropia			
0	0	0	no	0	emmetropia		y	
0	0	0	no	0	emmetropi	under	y	keratoconus
0	0	0	no	0	emmetropi	accurate		
180	-0.5	-0.75	no	-0.5	myopia	accurate	y	
180	0	-0.25	no	0	emmetropia		y	
0	0.25	-0.25	no	0.25	emmetropi	accurate		
0	0	0	no	0	emmetropi	accurate	y	
0	0.25	0.25	no	0.25	emmetropia		y	
0	0	0	no	0	emmetropi	accurate	y	
0	0	0	no	0	emmetropi	accurate	y	
0	0.25	0.25	no	0.25	emmetropia			
0	-0.38	0	no	0	emmetropi	accurate	y	
0	4	4	no	4	hypermetr	accurate	y	
0	9.5	9.5	no	9.5	hypermetropia			
0	0	0	no	0	emmetropi	accurate		
0	0	0	no	0	emmetropi	accurate		
180	-1	-1	no	-1	myopia	accurate		
180	0.5	0.5	yes	0.5	emmetropi	accurate		
180	2	2	yes	2	hypermetropia		full	
0	0.5	0.5	no	0.5	emmetropi	accurate		
0	0	0	no	0	emmetropi	accurate		
0	-1.5	-1.5	no	-1.5	myopia			
180	1	1	yes	1	emmetropia			
180	-0.25	-0.25	no	-0.25	emmetropia			
180	-0.13	0.13	no	0.13	emmetropi	accurate	full	
0	0.25	0.25	no	0.25	emmetropia			
180	-0.63	-0.25	yes	-0.25	myopia			
	0	0	no	0	emmetropi	accurate	full	
180	-0.25	-0.25	no	-0.25	emmetropi	accurate	full	
180	1	0.5	yes	0.5	emmetropi	accurate	full	
180	2.25	2.13	no	2.13	hypermetr	under	inconclusive	
0	1	1.25	no	1	emmetropi	accurate	inconclusive	
0	0.88	0.75	no	0.75	emmetropi	accurate	inconclusive	
0	0.25	0.25	no	0.25	emmetropi	accurate	Full R+L	
0	1.25	1.25	no	1.25	emmetropi	inconclusiv	inconclusive	
170	4.63	4.75	yes	4.63	hypermetr	accurate	inconclusive	

80	0.25	0.13	no		0.13	emmetropi	accurate	inconclusive
0	0	0	no		0	emmetropi	accurate	inconclusive
0	0.13	0.25	no		0.13	emmetropi	accurate	Full R+L
0	0.25	0.25	no		0.25	emmetropi	accurate	
180	0.63	0.63	yes		0.63	emmetropi	accurate	
0	1.25	1.25	no		1.25	emmetropia		
180	0.25	0.38	no		0.25	emmetropi	accurate	Full R+L
0	0	0	no		0	emmetropi	accurate	
0	0.25	0.25	no		0.25	emmetropi	accurate	inconclusive
0	0	0	no		0	emmetropi	accurate	inconclusive
90	0	-0.25	no		0	emmetropi	accurate	
0	0	-0.25	no		0	emmetropia		
0	2	2	no		2	hypermetropia		
0	1	1	no		1	emmetropia		
0	0.5	0.5	no		0.5	emmetropia		
0	0	0	no		0	emmetropi	accurate	Full R+L
180	0.13	-0.25	no		0.13	emmetropi	accurate	Full R+L
80	0.75	0.38	no		0.38	emmetropi	accurate	
0	0	-0.5	no		0	myopia	inconclusiv	inconclusive
175	-0.5	-0.5	yes		-0.5	emmetropia		
5	3.75	4	yes		3.75	hypermetr	under	Full R+L
0	0	0	no		0	emmetropi	accurate	
0	0	0	no		0	emmetropi	accurate	
0	0	0	no		0	emmetropi	accurate	
0	0	0	no		0	emmetropi	accurate	Full R+L
0	0	0	no		0	emmetropi	accurate	
0	0.75	0.5	no		0.5	emmetropi	accurate	
90	-0.25	-0.38	no		-0.25	emmetropi	accurate	
0	0	0	no		0	emmetropi	accurate	
0	0	0	no		0	emmetropi	accurate	
180	0.5	0	yes		0	emmetropi	accurate	
90	0.75	0.38	no		0.38	emmetropi	accurate	
0	-0.25	-0.5	no		-0.25	myopia	accurate	
0	0	0	no		0	emmetropi	accurate	
0	0	0	no		0	emmetropi	accurate	
0	0	0	no		0	emmetropi	accurate	
0	0	0	no		0	emmetropi	under	
5	3.75	3.25	yes		3.25	hypermetr	inconclusiv	inconclusive
0	0	0	no		0	emmetropi	accurate	
165	-1.25	-2.5	yes		-1.25	myopia	inconclusiv	inconclusive
120	0	-0.13	no		0	emmetropi	under	
65	-1.75	-0.88	no		-0.88	myopia	accurate	
25	0.5	0.88	yes		0.5	emmetropi	under	
0	0	0	no		0	emmetropia		
0	0	0	no		0	emmetropia		
0	0.5	0.5	no		0.5	emmetropi	accurate	
85	0.5	-0.88	yes		0.5	emmetropi	accurate	
100	0.25	0.25	no		0.25	emmetropi	accurate	
	0	0	no		0	emmetropi	accurate	full
	0.75	0.75	no		0.75	emmetropi	accurate	full

10	1.38	1.38	no	1.38	emmetropi	accurate	full	
180	1	0.75	no	0.75	emmetropia		unclear	
180	-5.13	-5.13	no	-5.13	myopia	accurate	full	
180	-4	-3	yes	-4	myopia	accurate	unclear	
	-13.5		yes	1	myopia	accurate	full	
180	3.75	3.5	yes	3.5	hypermetr	accurate	full	
180	3.75	6.5	yes	3.75	hypermetr	accurate	full	
180	1.75	2.5	no	1.75	hypermetr	accurate	full	
180	2.63	2.63	no	2.63	hypermetr	accurate	r. Hemiano	Hemianopia
140	-0.5	-0.5	yes	-0.5	myopia	inconclusiv	inconclusive	
	2	1.25	no	1.25	hypermetr	accurate	full	
180	6	6.25	yes	6	hypermetr	accurate	inconclusive	
90	1.63	1.63	no	1.63	emmetropi	accurate	full	
180	-6.25	-5.75	yes	-5.75	myopia	accurate	inconclusive	
	2.5	2.5	no	2.5	hypermetr	accurate	full	
	1.5	1.5	no	1.5	emmetropi	inconclusiv	full	
	2	2	no	2	hypermetr	inconclusiv	full	
180	-1.25	-2.88	yes	-1.25	myopia	accurate	full	
180	2	1.5	no	1.5	hypermetr	inconclusiv	full	
	2	2	no	2	hypermetr	accurate	full	
	0.25	0.25	no	0.25	emmetropi	accurate	full	
	2.25	2.25	no	2.25	hypermetr	accurate	full	
	1.25	1.25	no	1.25	emmetropi	accurate	full	
180	-3.75	-1.75	no	-1.75	myopia	inconclusiv	full	
	1.25	1.25	no	1.25	emmetropi	inconclusiv	no co-op	
180	0.25	0.25	no	0.25	emmetropi	inconclusiv	full	
180	2.5	2.5	yes	2.5	hypermetr	accurate	inconclusive	
180	1.38	3.13	yes	1.38	hypermetr	accurate	full	
180	6.75	7.13	no	6.75	hypermetr	accurate	full	
180	2	2	no	2	hypermetr	inconclusive		
180	2.25	2.25	no	2.25	hypermetr	inconclusiv	full	
180	2.63	2.63	no	2.63	hypermetr	accurate	full	
20	2.75	2.25	no	2.25	hypermetr	accurate	full	
180	-3	-2	yes	-2	myopia	inconclusiv	inconclusive	
180	1.75	1.75	no	1.75	emmetropi	inconclusiv	full	
170	2.88	2.88	no	2.88	hypermetr	accurate	full	
90	2	2	no	2	hypermetr	accurate	r hemianop	hemianopia
	1.25	1.25	no	1.25	emmetropi	accurate	full	
	1	1	no	1	emmetropi	accurate	full	
180	3	3.25	yes	3	hypermetr	inconclusiv	full	
180	1.63	1.88	no	1.63	emmetropi	accurate	full	
	3.5	4	yes	3.5	hypermetr	accurate	full	
180	2.75	3.25	no	2.75	hypermetr	accurate	full	
180	1.5	0.88	no	0.88	emmetropi	accurate	full	
	0	0	no	0	emmetropi	accurate	full	
180	-0.5	-12.5	yes	-0.5	myopia	accurate	L upper los ROP LE	
	0	0	no	0	emmetropi	accurate	full	
	3	3	no	3	hypermetr	under	full	
	0	0	no	0	emmetropi	accurate	full	
	5.5	6	no	5.5	hypermetropia		full	

	0	0	no	0	emmetropi	accurate		
90	-0.5	-0.5	yes	-0.5	myopia	accurate	full	
	6	4.5	no	4.5	hypermetr	accurate	full	
	0	0	no	0	emmetropia		full	
	0	0	no	0	emmetropi	accurate	full	
	0	0	no	0	emmetropia			
180	-6.5	-7.5	yes	-6.5	myopia	accurate	full	ROP BES
180	1.25	1	yes	1	emmetropi	under		Blepharitis
	0	0	no	0	emmetropi	accurate		
	0	0	no	0	emmetropia		full	
	0	0	no	0	emmetropi	accurate	full	
180	-0.5	-0.25	yes	-0.25	myopia		inconcl	chalazion Rlid
180	-10.25	-10.25	yes	-10.25	myopia	under		
90	0	0	yes	0	emmetropia		full	
180	0.5	0.5	yes	0.5	emmetropi	accurate	full	
180	3	3	yes	3	hypermetr	accurate	full	CVI susp
7	-2.5	-2	yes	-2	myopia		full	
	1.5	1.5	no	1.5	emmetropi	accurate	full	
	0	0	no	0	emmetropi	accurate	full	
	0	0	no	0	emmetropi	accurate	full	
	0	0	no	0	emmetropi	accurate		
180	2	2	yes	2	hypermetr	under	full	
90	-4.5	-4.5	yes	-4.5	myopia	under		CVI susp
180	-8.25	-8.25	no	-8.25	myopia	accurate		
	0	0	no	0	emmetropia			
165	2.75	4	yes	2.75	hypermetr	accurate		CVI
	1	1	no	1	emmetropi	accurate	full	
	3	2	no	2	hypermetropia		full	
	0	0	no	0	emmetropi	accurate	full	
180	0	0.25	yes	0	emmetropia			CVI
	4	5	no	4	hypermetr	under		CVI
	0	0	no	0	emmetropia			CVI
	0.75	0.75	no	0.75	emmetropi	accurate	inconcl	
	1.5	1.5	no	1.5	emmetropi	under		
180	0	0	yes	0	emmetropia		full	
	1	1	no	1	emmetropi	accurate	full	mild GPC
	5.5	6.5	yes	5.5	hypermetr	under	Ltotalhemi	CVI, hemianopia
	0.5	0.5	no	0.5	emmetropi	accurate	full	
	1	1	no	1	emmetropi	accurate	full	
	1	1	no	1	emmetropi	accurate		
	0.5	0.5	no	0.5	emmetropi	accurate	full	
	1	1	no	1	emmetropi	accurate	inconcl	
	4	3	no	3	hypermetr	accurate	full	
	1.25	1.25	no	1.25	emmetropi	accurate	full	
	1	1	no	1	emmetropi	accurate	full	
	1.25	1.25	no	1.25	emmetropia			
	1.5	1.5	no	1.5	emmetropi	accurate	full	
	0	0	no	0	emmetropi	accurate	full	
	0	0	no	0	emmetropi	accurate	inconcl	
	8	8	no	8	hypermetropia		full	

	0	0	no	0	emmetropi	accurate	full	
	0	0	no	0	emmetropi	accurate		
	0	0	no	0	emmetropia			
180	0	0.25	yes	0	emmetropia			CVI
	0	0	no	0	emmetropi	accurate	full	
	0	0	no	0	emmetropi	accurate	full	
180	-0.25	-0.25	no	-0.25	emmetropi	accurate	full	conjunctivitis
	0	0	no	0	emmetropi	accurate	full	
	-0.5	-0.5	no	-0.5	myopia	accurate	full	
	0	0	no	0	emmetropi	accurate	full	
180	4	4	yes	4	hypermetr	under	full	
	0	0	no		emmetropi	accurate		
180	-9	-9	yes		myopia			
	3.5	5.5	no		hypermetropia			
	0	0	no		emmetropi	accurate		
	0	0	no		emmetropi	accurate		
	0	0	no		emmetropi	accurate		
180	0.75	0.75	yes		emmetropi	under		
	0	0	no		emmetropi	accurate		
	0	0	no		emmetropi	under		
	0	0	no		emmetropi	accurate		
	7		no		hypermetropia			cataract
	0	0	no		emmetropi	accurate		hemianopia
	0.5	0.5	no		emmetropi	accurate		
180	0.5	0.5	no		emmetropi	accurate		
	1	1	no		emmetropi	accurate		
	0.5	0.5	no		emmetropi	accurate		
	0	0	no		emmetropi	accurate		
	0.75	0.75	no		emmetropia			
180	-0.75	-0.5	yes		myopia			sl R ptosis/excer
	0	0	no		emmetropi	under		
	-2	-1.5	no		myopia	accurate		
	-1	-1	no		myopia			
	-0.5	-0.75	no		myopia	accurate		
	0.5	0.5	no		emmetropi	accurate		
	1.5	-0.5	no		myopia	accurate		
	0	0	no		emmetropi	accurate		
	0	0	no		emmetropi	accurate		
	2.5	3.25	no		hypermetropia			
180	-0.63	-0.63	yes		myopia	accurate		
5	2.62	3.12	yes		hypermetr	under		
	0	0	no		emmetropi	accurate		allergic conjunct
15	0	1	yes		emmetropi	accurate		
	-0.5	-0.5	no		myopia	accurate		
	0	0	no		emmetropi	accurate		excema
	-0.5	-0.5	no		myopia	accurate		
	0	0	no		emmetropi	accurate		
	0	0	no		emmetropi	accurate		
	-1	-0.75	no		myopia	accurate		
	0	0	no		emmetropia			

	-0.5	-0.5	no		myopia	accurate		
170	-7.88	8.13	yes		myopia	accurate		
	0	0	no		emmetropi	accurate		
	0	0	no		emmetropi	accurate		
	4	3	no		hypermetropia			
130	3.38	3.62	no		hypermetr	accurate		L inf retinal colo
	0	0	no		emmetropi	accurate		hemianopia
	0	0	no		emmetropi	accurate		hemianopia
	0	0	no		emmetropi	accurate		
	0.5	0.5	no		emmetropi	accurate		
	0	0	no		emmetropi	accurate		
	-0.75	-0.75	no		myopia			
	-0.5	-0.5	no		myopia	accurate		
175	0.75	1.38	yes		emmetropia			
	0	0	no		emmetropi	accurate		
	0	0	no		emmetropi	accurate		
	2	2	no		hypermetr	under		
	-0.75	-0.75	no		myopia	accurate		
	0	0	no		emmetropi	accurate		
	0	0	no		emmetropi	accurate		
175	1.88	-0.75	yes		myopia	accurate		
	0	0	no		emmetropi	accurate		
5	-0.38	-0.13	yes		emmetropi	accurate		
	0	0	no		emmetropi	accurate		
	0	0	no		emmetropi	accurate		
	0	0	no		emmetropi	accurate		
	0	0	no		emmetropi	accurate		
	0	0	no		emmetropi	accurate		
	-3.5	-3.5	no		myopia	accurate		
180	-0.5	-0.5	yes	-0.5	myopia		full	
0	2	2	no	2	hypermetropia		full	
0	-0.25	-0.25	no	-0.25	emmetropi	accurate	full	
0	0.5	0.25	no	0.25	emmetropi	accurate	full	
180	-2.5	-2.5	yes	-2.5	myopia			
180	-1.25	-1.25	yes	-1.25	myopia	accurate	full	
0	0.5	0.5	no	0.5	emmetropi	accurate	full	
0	-0.5	-0.5	no	-0.5	myopia			
0	0.25	0.25	no	0.25	emmetropia			
0	0.5	0.5	no	0.5	emmetropi	accurate	full	
170	0	0	yes	0	emmetropia			
0	-0.25	-0.25	no	-0.25	emmetropi	accurate		
0	1.25	1.25	no	1.25	emmetropi	accurate		
0	1	1	no	1	emmetropi	accurate	full	
0	0	0	no	0	emmetropia			
0	0.5	0.5	no	0.5	emmetropi	accurate		
0	0.5	0.25	no	0.25	emmetropi	accurate		
90	-0.25	-0.25	no	-0.25	emmetropia			
0	1	1	no	1	emmetropia			
0	1	1	no	1	emmetropia			
0	0.5	0.5	no	0.5	emmetropia			

0	0	0	no	0	emmetropia			
180	-1.13	-1.13	no	-1.13	myopia	accurate		
90	-0.38	-0.38	no	-0.38	emmetropi	accurate	full	
180	-0.25	-0.25	no	-0.25	emmetropia			
180	2	2	yes	2	hypermetropia		left reducti	field defect
0	0.5	0.5	no	0.5	emmetropia			
180	1	1	yes	1	emmetropia		full	
0	1	1	no	1	emmetropia			cataract
170	2.5	2.75	yes	2.5	hypermetropia		full	
0	0	0	no	0	emmetropia		full	
180	-1.5	-0.5	yes	-0.5	myopia		full	
0	0.5	0.5	no	0.5	emmetropia			
180	3.63	3.63	no	3.63	hypermetr	accurate		
180	4.75	4.75	yes	4.75	hypermetropia			
90	-0.5	-0.5	yes	-0.5	myopia			
180	-0.38	-0.38	no	-0.38	emmetropia			
0	0.5	0.5	no	0.5	emmetropi	accurate		
0	0	0	no	0	emmetropia			
0	0.5	0.5	no	0.5	emmetropi	accurate	full	
180	0.25	0.25	no	0.25	emmetropia			
180	1.25	1.25	yes	1.25	emmetropia			
180	0.25	0.25	no	0.25	emmetropi	accurate	full	
180	1	-0.25	yes	-0.25	emmetropia			
180	1	1	yes	1	emmetropia			
0	0	0	no	0	emmetropi	accurate	y	
0	0	0	no	0	emmetropi	accurate	y	
0	0	0	no	0	emmetropi	accurate	y	
0	0	0	no	0	emmetropia			
0	6.5	6.5	no	6.5	hypermetropia			
	0	0	no	0	emmetropia		full	
100	5.75	7.25	yes	5.75	hypermetropia			
180	-2.88	-5.63	no	-2.88	myopia			
90	0	0.25	no	0	emmetropia			
180	0.13	0.13	no	0.13	emmetropia			Marcus Gunn Sy
70	1.38	1.5	yes	1.38	emmetropia			
90	-0.63	-0.63	no	-0.63	myopia			
120	4.63	4.5	yes	4.5	hypermetropia			
	3	0	no	0	hypermetropia			
0	0	0	no	0	emmetropia			
90	-1	-0.75	no	-0.75	myopia			
	-0.63	-0.5	no	-0.5	myopia			
100	-0.13	0.63	yes	-0.13	myopia			
	-0.5	-0.5	no	-0.5	myopia			
	0	-0.5	no	0	myopia			
	0.5	0	no	0	emmetropia			
180	3	4.25	no	3	hypermetropia			
180	-5.75	-6.25	no	-5.75	myopia			
90	-19.63	-15.63	no	-15.63	myopia			

	-0.5	-0.75	no	-0.5	myopia			
	0	0.75	no	0	emmetropia			
180	0.88	1	no	0.88	emmetropia			
	-1.75	-2	no	-1.75	myopia			
90	0.25	1.25	no	0.25	emmetropia			
	-1	-1	no	-1	myopia			
	-2	-2	no	-2	myopia			
180	-17.5	-10.13	no	-10.13	myopia			
	-1.5	-0.5	yes	-0.5	myopia			
	-1.38	-1	no	-1	myopia		full	
90	0.25	0.25	no	0.25	emmetropia		full	
85	-3.88	-3.25	yes	-3.25	myopia			
	-2	-1.5	no	-1.5	myopia			
2	3	1.5	yes	1.5	hypermetropia			
170	0.13	-0.5	yes	0.13	myopia		full	
	0	0	no	0	emmetropia			
180	-0.25	0.63	no	-0.25	myopia		full	
180	-0.13	-0.38	no	-0.13	emmetropia			
	-1.5	-1.5	no	-1.5	myopia			
	0	0	no	0	emmetropia			
180	3.5	3.5	yes	3.5	hypermetropia		fullBEO	
								disc pallor
0	0.25	0.25	no	0.25	emmetropi	accurate	full	
0	-0.25	-0.25	no	-0.25	emmetropi	accurate		
0	2	2	no	2	hypermetropia			
180	2.5	0.75	yes	0.75	hypermetr	accurate	full	lid chalazion
0	0	0	no	0	emmetropi	accurate	full	
0	-0.25	-0.25	no	-0.25	emmetropi	accurate	full	
180	0.25	0.25	no	0.25	emmetropi	accurate	full	
0	0.5	0.5	no	0.5	emmetropi	accurate		
0	0.5	0.5	no	0.5	emmetropia			
0	0.5	0.5	no	0.5	emmetropia			
180	0.25	0.25	no	0.25	emmetropia			
180	-0.25	-0.25	no	-0.25	emmetropia			
0	-5.5	-4	yes	-4	myopia			
180	-4.5	2	yes	2	myopia			
0	0	0	no	0	emmetropia			
0	0	0	no	0	emmetropi	accurate	full	
0	0	0	no	0	emmetropi	accurate	full	
180	0	0	yes	0	emmetropi	under		
180	-0.13	-0.5	yes	-0.13	myopia	accurate	full	
180	0.75	0.5	no	0.5	emmetropi	accurate	full	
0	0	0	yes	0	emmetropi	accurate		
	3.75	3.75	no	3.75	hypermetr	accurate		
	0	0	no	0	emmetropia			
	0	0	no	0	emmetropi	accurate	FULL	
180	-0.38	-0.38	yes	-0.38	emmetropi	accurate		
	4	4	no	4	hypermetropia			
	0	0	no	0	emmetropi	accurate	FULL	
	-1.75	-1.75	no	-1.75	myopia	accurate	full	

	0	0	no	0	emmetropi	accurate	FULL	
	0.5	0.5	no	0.5	emmetropi	accurate	full	
	0	0	no	0	emmetropi	accurate		
	0.5	0.5	no	0.5	emmetropi	accurate	full	
	0	0	no	0	emmetropi	accurate		
	0.5	0.5	no	0.5	emmetropi	accurate	full	
	0	0	no	0	emmetropi	accurate	full	
180	4.5	5	yes	4.5	hypermetr	accurate		
180	1.25	1.75	yes	1.25	emmetropia			
20	6.88	5.5	no	5.5	hypermetr	accurate	full	
165	3.25	2.5	yes	2.5	hypermetr	under		
0	-0.75	-0.75	no	-0.75	myopia	accurate	full BEO	
0	0	0	no	0	emmetropi	accurate	full BEO	
180	0.5	1	yes	0.5	emmetropia			
0	0	0	no	0	emmetropi	accurate	full BEO	
180	-14.5	-14.75	yes	-14.5	myopia			Coloboma iris/L
0	0	0	no	0	emmetropi	accurate	FullBEO	
0	0.5	0.5	no	0.5	emmetropi	accurate	full BEO	
0	0	0	no	0	emmetropia			
0	1	1	no	1	emmetropi	accurate	full BEO	
0	-2	-2	no	-2	myopia	accurate	full BEO	
0	0	0	no	0	emmetropi	accurate		
0	0	0	no	0	emmetropi	accurate		
0	0.5	0.5	no	0.5	emmetropi	accurate	full BEO	
180	-1	-1	yes	-1	myopia			
0	1	1	no	1	emmetropi	accurate		
0	-1.5	0	yes	0	myopia			
0	0	0	no	0	emmetropi	under		
0	0	0	no	0	emmetropi	accurate	full BEO	
0	0	0	no	0	emmetropi	under		
0	0.5	0.5	no	0.5	emmetropi	accurate		
0	0	0	no	0	emmetropi	accurate	full BEO	
0	0	0	no	0	emmetropi	accurate	full BEO	
0	0	0	no	0	emmetropi	accurate	full BEO	
90	-0.63	-0.63	yes	-0.63	myopia	accurate	full BEO	
0	3.5	4	no	3.5	hypermetropia			
0	-1	-1	no	-1	myopia	accurate	full BEO	
180	-0.5	-0.5	yes	-0.5	myopia	accurate	full BEO	
0	0	0	no	0	emmetropi	accurate	full BEO	
0	0	0	no	0	emmetropi	accurate	full BEO	
0	-0.5	-0.5	no	-0.5	myopia	accurate	full BEO	
0	0	0	no	0	emmetropi	accurate	full BEO	
0	0	0	no	0	emmetropi	accurate	full BEO	
90	-0.75	-0.75	yes	-0.75	myopia	accurate	full BEO	
10	0.63	-0.5	yes	-0.5	myopia	accurate	full BEO	
180	2	1	yes	1	hypermetropia		full BEO	
0	-1	-1	no	-1	myopia			
0	2	2	no	2	hypermetropia		full BEO	
0	1	1	no	1	emmetropi	under	full BEO	allergic conj
0	-0.25	-0.25	no	-0.25	emmetropi	accurate	Full BEO	

0	-0.25	-0.25	no		-0.25	emmetropi	accurate	Full BEO
0	1	1	no		1	emmetropi	accurate	full BEO
180	1.5	1.5	yes		1.5	emmetropia		full BEO
0	1	1	no		1	emmetropi	accurate	full BEO
0	-0.5	-0.5	no		-0.5	myopia	accurate	

Outcome specs	Referral	Visual defect	Referral to QTVI	Reason for QTVI	CVI
no Rx		no concerns	other	gls	no
First time		not prev known	no		no
no Rx		vis def no info	other	glasses	no
no Rx		not prev known	no		no
No change		already known			no
no Rx	Orthoptist	not prev known	no		no
no Rx		not prev known	CVI + other	VI + CVI	yes
no Rx		no concerns	no		no
no Rx	GP	not prev known	no		no
no Rx		already known	other	VI	no
no Rx		no vis def	other	VI	no
no Rx		no vis def	no		no
no Rx		not prev known	no		no
no Rx		not prev known	other	VI	no
First time		not prev known	other	VI	no
First time		not prev known	other	glasses	no
no Rx		no concerns	other	glasses	no
no Rx	Orthoptist	not prev known	no		no
no Rx		no concerns	no		no
no Rx	Ophthalmol	already known	no		no
no Rx		not prev known	no		no
no Rx	Orthoptist	not prev known	CVI	VI+CVI	yes
First time		not prev known	CVI	CVI+VI	yes
First time	GP	already known	other	glasses	no
First time		already known	other	glasses	no
First time		not prev known	other	glasses	no
First time		already known	CVI	glasses + CVI	yes
no Rx		no concerns	other	glasses	no
no Rx		no vis def	no		no
no Rx		no vis def	no		no
no Rx		already known	no		no
no Rx		no vis def	CVI	CVI	yes
no Rx		already known	CVI	CVI	yes
First time		not prev known	CVI	CVI	yes
First time		not prev known	other	Rx	no
no Rx		already known	other	Rx	no
no Rx		already known	no		no
no Rx		no vis def	other	Rx	no
no Rx		already known	no		no
no Rx		no vis def	no		no
no Rx		no vis def	no		no
no Rx		no vis def	other	incomplete	no
no Rx		already known	CVI	CVI	yes
no Rx		already known	CVI + other	CVI nystagmus	yes
no Rx		no vis def	no	Rx	no
no Rx		no vis def	no		no

no Rx		no vis def	no		no
no Rx		already known	no		no
no Rx		no vis def	other	Rx path	no
no Rx		no vis def	no		no
no Rx		no vis def	no		no
no Rx		no vis def	no		no
no Rx		not prev known	no		no
no Rx		already known	no		no
no Rx		already known	CVI + other	CVI Rx	yes
no Rx		no vis def	no		no
First time		already known	no		no
no Rx		already known	other	Rx	no
no Rx		no vis def	no		no
no Rx		already known	no		no
First time		not prev known	other	RD	no
no Rx		no concerns	other	Rx	no
no Rx		no vis def	no		no
no Rx		no vis def	no		no
no Rx		no vis def	no		no
No change		no vis def	CVI	CVI	yes
First time	Orthoptist	vis def no info	no		no
no Rx		no vis def	other	Gls	no
no Rx		no vis def	no		no
no Rx		no vis def	CVI	CVI	yes
No change		vis def no info	no		no
no Rx	Orthoptist	vis def no info	no		no
First time		vis def no info	CVI	CVI	yes
no Rx		vis def no info	other	Gls	no
no Rx		no vis def	no		no
no Rx		vis def no info	no		no
First time	Orthoptist	vis def no info	no		no
no Rx		no vis def	other	Gls	no
First time		vis def no info	CVI	CVI	yes
no Rx	Orthoptist	vis def no info	other	VI and Gls	no
no Rx		no vis def	no		no
First time	Orthoptist	vis def no info	no		no
First time		vis def no info	no		no
First time		vis def no info	other	Gls	no
no Rx		no concerns	other	gls	no
First time		vis def no info	no		no
no Rx		no concerns	CVI + other	gls CVI	yes
no Rx		vis def no info	no		no
First time		already known	no		no
No change		already known	other	Glasses	no
No change		already known	CVI	?CVI	yes
First time		already known	other	Glasses	no
no Rx		no vis def	other	Glasses	no
no Rx		no vis def	no		no
no Rx		no vis def	no		no
no Rx		no vis def	no		no

no Rx		no vis def	no		no
no Rx		no vis def	no		no
no Rx		no vis def	no		no
no Rx		no vis def	no		no
First time		already known	no		no
no Rx		already known	other	Glasses	no
First time		already known	other	INCONCLUSIVE	no
No change		already known	other	Glasses	no
no Rx		no vis def	other	Glasses	no
no Rx		no vis def	no		no
no Rx		already known	no		no
no Rx		no vis def	CVI	?CVI	yes
no Rx		no vis def	no		no
no Rx		not prev known	no		no
no Rx		no vis def	CVI	?CVI	yes
No change		already known	no		no
no Rx		no vis def	other	Glasses	no
No change		already known	no		no
No change		already known	other	Glasses	no
no Rx		already known	other	Glasses	no
First time		not prev known	other	nystagmus	no
no Rx		no vis def	other	nystagmus/glass	no
First time		not prev known	no		no
First time		already known	other	Glasses	no
First time		not prev known	CVI + other	Glasses/?CVI	yes
No change		already known	other	Glasses	no
no Rx		no vis def	other	Glasses	no
no Rx		not prev known	no		no
no Rx		no vis def	CVI	?CVI	yes
no Rx		no vis def	no		no
no Rx		no vis def	no		no
no Rx		no vis def	no		no
no Rx		no vis def	no		no
no Rx		already known	no		no
no Rx		no vis def	other	SSI	no
no Rx		no vis def	no		no
no Rx			no		no
no Rx		already known	no		no
no Rx		no vis def	other	SSI	no
no Rx		no vis def	no		no
no Rx		not prev known	no		no
no Rx		no vis def	other	MONOC EFF	no
No change		already known	no		no
No change		already known	other	Glasses	no
First time		not prev known	other	Glasses	no
no Rx		not prev known	other	Glasses	no
No change		already known	no		no
no Rx		no vis def	other	Glasses	no
No change	Ophthalmol	already known	no		no
No change		already known	CVI + other	CVI+Glasses+VI	yes

No change		already known	other	gls	no
no Rx		no vis def	other	gls VI	no
no Rx		no vis def	no		no
no Rx		already known	no		no
First time		not prev known	CVI	CVI	yes
no Rx		no vis def	other	Glasses	no
No change		already known	no		no
No change		already known	other	glasses	no
No change		already known	other	Glasses	no
no Rx		already known	other	Glasses	no
no Rx		no vis def	CVI	?CVI	yes
no Rx		no vis def	no		no
no Rx		already known	no		no
no Rx		no vis def	CVI + other	CVI NYSTAGMUS	yes
no Rx		no vis def	no		no
no Rx		no vis def	no		no
no Rx		already known	no		no
First time		not prev known	other	SSI	no
no Rx		no vis def	other	Glasses	no
no Rx		no vis def	no		no
no Rx		already known	no		no
No change		already known	other	Glasses	no
no Rx		vis def no info	other	Glasses/VI	no
No change		already known	other	inconclusive	no
no Rx		no vis def	other	Glasses	no
no Rx		already known	no		no
no Rx		already known	other	Glasses	no
no Rx		already known	CVI	CVI	yes
First time		no vis def	CVI	CVI	yes
no Rx		no vis def	no		no
no Rx		no vis def	no		no
no Rx		no vis def	no		no
No change		already known	no		no
no Rx		no vis def	no	glasses/NYSTAGN	no
no Rx	Ophthalmol	already known	no		no
no Rx		no vis def	other	SSI	no
No change		already known	no		no
no Rx		no vis def	other	Glasses	no
no Rx		no vis def	no		no
no Rx		no vis def	no		no
No change		already known	no		no
no Rx		no vis def	other	Glasses	no
no Rx		no vis def	no		no
no Rx		already known	no		no
no Rx		no vis def	no		no
no Rx		no vis def	no		no
no Rx		already known	no		no
no Rx		already known	CVI + other	CVI/VF DEFECT	yes
no Rx		no vis def	no		no
No change		already known	no		no

no Rx		no vis def	other	Ocular/VF defect	no
no Rx		no vis def	no		no
First time		not prev known	no		no
no Rx		no vis def	other	Glasses	no
no Rx		no vis def	no		no
no Rx		no vis def	no		no
no Rx		already known	no		no
Change in Rx		already known			no
First time		not prev known		Glasses	no
no Rx		not prev known	other	Glasses	no
No change		already known	other	EOM control prol	no
Change in Rx		already known	CVI + other	Glasses/?CVI inv	yes
no Rx		no vis def	other	glasses/NYSTAGM	no
no Rx		no vis def	no		no
No change		already known	no		no
Change in Rx		already known	CVI + other	Glasses/?CVI inv	yes
no Rx		no vis def	other	glasses/squint	no
no Rx		no vis def	no		no
no Rx		no vis def	no		no
no Rx		no vis def	no		no
no Rx		already known	no		no
No change		already known	CVI + other	Glasses/?CVI inv	yes
no Rx		already known	other	Glasses	no
no Rx			CVI + other	Glasses/?CVI	yes
no Rx		no concerns			no
no Rx		no concerns	no		no
no Rx		no concerns	no		no
no Rx		no concerns	no		no
no Rx		no concerns	no		no
Change in Rx	Ophthalmol	already known	no		no
no Rx		no concerns	other	gls	no
no Rx		no concerns	no		no
no Rx		no concerns	no		no
no Rx		no concerns	no		no
First time		not prev known	no		no
no Rx		no concerns	other	gls	no
no Rx		no concerns	no		no
no Rx		no concerns	no		no
no Rx		no concerns	no		no
no Rx		no concerns	no		no
First time	Orthoptist	already known	no		no
First time	Orthoptist	not prev known	other	gls	no
No change		already known	other	gls	no
no Rx	Ophthalmol	already known	other	gls	no
no Rx		already known	other	vi	no
no Rx		no concerns	no	n	no
no Rx		no concerns	no		no
no Rx		no concerns	no		no
no Rx		no concerns	no		no
no Rx		vis def no info	no		no

no Rx	Orthoptist	no concerns	other	upgaze	no
First time	Orthoptist	vis def no info	other		no
no Rx		no concerns	other	glasses	no
No change		already known	no		no
No change		already known	other	glasses	no
no Rx		no vis def	other	glasses	no
no Rx		no concerns	CVI	cvi	yes
No change		already known	no		no
no Rx		no vis def	CVI + other	gls VI/CVI	yes
First time		already known	no		no
No change		already known	other	gls	no
No change		already known	other	VI gls	no
no Rx		already known	CVI + other	CVI/gls	yes
no Rx		no concerns	CVI	CVI/VI	yes
no Rx		already known	no		no
no Rx		no concerns	no		no
No change		already known	no		no
no Rx		no vis def	other	gls	no
no Rx		no concerns	no		no
Replace FWT		already known	no		no
no Rx		no vis def	other	gls	no
no Rx		already known	no		no
no Rx		no concerns	CVI	CVI	yes
Replace FWT		already known	no		no
No change		already known	other	gls	no
no Rx		no concerns	other	gls	no
no Rx		already known	no		no
No change		already known	other	VI	no
no Rx		no concerns	other	gls	no
no Rx		no vis def	no		no
no Rx		no vis def	no		no
Change in Rx		already known	no		no
no Rx		no vis def	other	gls	no
No change		already known	no		no
no Rx		no concerns	CVI + other	gls/CVI	yes
no Rx		no concerns	no		no
no Rx		no concerns	CVI	CVI	yes
no Rx			no		no
Change in Rx		already known	no		no
no Rx		already known	other	gls	no
no Rx		already known	no		no
Replace FWT		already known	CVI	CVI	yes
no Rx		no concerns	other	gls	no
No change		already known	no		no
First time		not prev known	other	gls	no
Change in Rx		already known	other	gls	no
no Rx		already known	CVI + other	gls/CVI	yes
First time		not prev known	other	VI	no
no Rx		already known	other	gls	no
no Rx		no concerns	other	VI	no

No change		already known	no		no
Replace FWT		already known	other	gls	no
no Rx		no concerns	CVI	Y	yes
no Rx		no vis def	no		no
Change in Rx		already known	no		no
no Rx		no concerns	CVI + other	gls/CVI	yes
no Rx		no concerns	no		no
no Rx		no vis def	no		no
Change in Rx		already known	no		no
no Rx		no vis def	no		no
no Rx	GP	not prev known	no		no
Change in Rx		already known	no		no
Replace FWT		already known	other	gls	no
No change		already known	other	gls	no
No change		already known	other	gls	no
no Rx		no concerns	other	gls	no
no Rx		no concerns	no		no
Replace FWT		already known	no		no
Change in Rx		already known	CVI + other	gls/CVI	yes
no Rx		already known	CVI + other	gls/CVI	yes
First time		already known	no		no
No change		already known	other	gls	no
no Rx		no concerns	other	gls/nystagmus	no
First time		already known	no		no
no Rx		no vis def	other	gls	no
no Rx		no concerns	no		no
First time		not prev known	no		no
no Rx		no vis def	other	gls	no
no Rx		no vis def	no		no
no Rx		no vis def	no		no
no Rx			no		no
no Rx		no vis def	no		no
no Rx		no concerns	no		no
No change		already known	no		no
First time		not prev known	CVI + other	gls/?CVI	yes
Change in Rx	Orthoptist	already known	other	gls	no
no Rx		no vis def	other	gls	no
First time		already known	no		no
no Rx		no vis def	other	gls	no
no Rx		no concerns	no		no
no Rx		already known	no		no
First time		already known	CVI	CVI	yes
Discontinue		already known	other	gls	no
no Rx	Orthoptist	not prev known	CVI	CVI	yes
Replace FWT		already known	no		no
no Rx		no vis def	other	glasses	no
no Rx		no vis def	no		no
no Rx		no vis def	no		no
Change in Rx		already known	no		no
Replace FWT		already known	CVI + other	CVI/glasses	yes

First time		not prev known	other	glasses	no
no Rx		no vis def	other	glasses	no
no Rx		no vis def	no		no
no Rx		no vis def	no		no
no Rx		no vis def	no		no
Change in Rx	Ophthalmol	already known	no		no
First time	Ophthalmol	already known	other	glasses	no
no Rx		no vis def	other	glasses	no
no Rx		no vis def	no		no
no Rx		no vis def	no		no
no Rx		no vis def	no		no
First time	Ophthalmol	not prev known	no		no
no Rx		already known	other	gls/ptosis	no
no Rx		no vis def	no		no
no Rx		no vis def	no		no
no Rx		no vis def	no		no
no Rx		already known	no		no
no Rx	GP	no vis def	CVI	CVI	yes
no Rx		no vis def	no		no
no Rx		no vis def	no		no
no Rx		no vis def	no		no
no Rx		no vis def	no		no
no Rx		no vis def	no		no
no Rx		no vis def	no		no
no Rx		no vis def	no		no
no Rx		already known	no		no
no Rx		no vis def	CVI	CVI	yes
First time		not prev known	no		no
no Rx		no vis def	other	glasses	no
no Rx		no vis def	no		no
no Rx		no vis def	no		no
no Rx		no vis def	no		no
no Rx		no vis def	no		no
no Rx		no vis def	no		no
First time		vis def no info	no		no
no Rx		no vis def	other	glasses	no
no Rx		no vis def	no		no
No change		already known	no		no
no Rx		no vis def	other	glasses	no
no Rx		not prev known	no		no
no Rx		not prev known	no		no
First time		not prev known	no		no
Discontinue		no vis def	other	glasses	no
Discontinue	Ophthalmol	already known	no		no
Discontinue		already known	CVI	CVI	yes
no Rx		no vis def	CVI	CVI	yes
no Rx		no vis def	no		no
no Rx		no vis def	no		no
No change		already known	no		no
no Rx			CVI	CVI	yes

no Rx		no vis def	no		no
Change in Rx		already known	no		no
no Rx		no vis def	other	gls	no
First time		not prev known	no		no
First time		already known	CVI + other	gls/?CVI	yes
no Rx		no vis def	CVI + other	CVI/glasses	yes
no Rx		no vis def	no		no
Replace FWT		already known	no		no
no Rx	Ophthalmol	not prev known	no		no
no Rx		not prev known	CVI	CVI	yes
no Rx	Ophthalmol	already known	CVI	CVI	yes
no Rx		already known	CVI	CVI	yes
no Rx		already known	CVI	CVI	yes
no Rx		already known	CVI	CVI	yes
no Rx		already known	CVI	CVI	yes
no Rx		already known	CVI	CVI	yes
no Rx		no vis def	other	Ocular	no
no Rx		no vis def	no		no
First time		not prev known	no		no
no Rx		no vis def	no		no
no Rx		no vis def	no		no
No change		already known	no		no
no Rx		no vis def	other	Ocular	no
no Rx		no vis def	no		no
no Rx		no vis def	no		no
no Rx		no vis def	no		no
no Rx		no vis def	no		no
no Rx		already known	no		no
First time		already known	other	Ocular	no
First time		not prev known	no		no
no Rx		no vis def	CVI + other	CVI/Ocular	yes
no Rx		no vis def	CVI	CVI	yes
no Rx		no vis def	no		no
no Rx		no vis def	no		no
No change		already known	no		no
no Rx		not prev known	CVI + other	CVI/glasses	yes
No change	Ophthalmol	already known	no		no
First time		already known	CVI + other	Glasses/ocular	no
no Rx		no vis def	CVI + other	CVI/glasses	yes
no Rx		no vis def	no		no
no Rx		no vis def	no		no
no Rx		no vis def	no		no
First time		already known	no		no
no Rx		no vis def	CVI + other	CVI/glasses	yes
No change		already known	no		no
no Rx		vis def no info	other	Gls	no
no Rx		no vis def	other	gls	no
no Rx		not prev known	no		no
No change		already known	no		no
No change		already known	CVI + other	CVI and VI	yes

no Rx		no concerns	CVI		yes
no Rx		already known	no		no
No change		already known	no		no
no Rx		vis def no info	other	Gls	no
no Rx		vis def no info	no		no
No change		already known	CVI	CVI	yes
no Rx		not prev known	CVI		yes
no Rx		already known	CVI		yes
No change		already known	no		no
no Rx		no concerns	CVI		yes
no Rx		vis def no info	no		no
no Rx		no concerns	other	vi	no
First time		not prev known	no		no
no Rx		vis def no info	CVI		yes
no Rx		no concerns	CVI + other	cvi and vi	yes
Change in Rx		already known	CVI	cvi	yes
no Rx		vis def no info	other	gls	no
Replace FWT		already known	CVI	CVI	yes
No change		already known	other	gls	no
no Rx		no concerns	CVI	CVI	yes
No change		already known	no		no
no Rx		no concerns	other	gls	no
no Rx		no concerns	no		no
No change		already known	no		no
First time		not prev known	CVI + other	gls/CVI	yes
no Rx		no concerns	other	gls	no
no Rx		no concerns	no		no
no Rx		no concerns	no		no
no Rx		already known	no		no
no Rx		no concerns	no		no
no Rx		no concerns	CVI	cvi	yes
no Rx		not prev known	no		no
Change in Rx		not prev known	other	vi	no
no Rx		no concerns	CVI + other	gls/CVI	yes
no Rx		no concerns	no		no
no Rx		no concerns	no		no
no Rx		no concerns	no		no
no Rx		no concerns	no		no
no Rx		no concerns	no		no
no Rx		no concerns	no		no
No change		vis def no info	CVI	cvi	yes
no Rx		no concerns	CVI + other	cvi glasses	yes
First time		not prev known	no		no
First time		not prev known	CVI + other	gls/CVI	yes
no Rx		no concerns	CVI + other	gls/CVI	yes
no Rx	Ophthalmol	already known	no		no
First time	Orthoptist	not prev known	CVI	cvi	yes
Replace FWT		already known	CVI	gls/CVI	yes
no Rx		no concerns	other	gls	no
no Rx		no concerns	no		no

First time		not prev known	no		no
Change in Rx		already known	CVI + other	gls/CVI	yes
Change in Rx		already known	CVI + other	gls/CVI	yes
no Rx		no concerns	CVI + other	gls/CVI	yes
no Rx		no concerns	no		no
no Rx		not prev known	no		no
no Rx		no concerns	other	gls/VI	no
no Rx		vis def no info	no		no
no Rx		no vis def	CVI + other	gls/CVI	yes
no Rx		already known	no		no
Change in Rx	Orthoptist	already known	other	hemi	no
no Rx		no concerns	no	gls	no
Change in Rx	Orthoptist	already known	no		no
First time		not prev known	no	gls	no
no Rx		no concerns	other	gls	no
First time	Orthoptist	already known	other	visual guided mo	no
no Rx		no concerns	no	gls	no
no Rx	Orthoptist	already known	no		no
no Rx	Orthoptist	vis def no info	CVI	cvi	yes
no Rx		already known	no		no
no Rx		no concerns	no	VI	no
no Rx		no concerns	no		no
no Rx		no concerns	no		no
no Rx		no concerns	no		no
no Rx		no concerns	no		no
no Rx		no concerns	no		no
Change in Rx		already known	no		no
First time		already known	no	gls	no
no Rx	GP	not prev known	other	gls	no
no Rx		no concerns	no		no
no Rx		no concerns	no		no
First time		not prev known	no		no
No change		already known	other	gls	no
no Rx		no concerns	other	gls	no
no Rx		no concerns	CVI	cvi	yes
no Rx		already known	no		no
no Rx	GP	not prev known	other	VI	no
no Rx		no concerns	CVI	cvi	yes
no Rx		no concerns	no		no
no Rx		no concerns	no		no
no Rx		no concerns	no		no
no Rx		no vis def	no		no
no Rx		no concerns	no		no
No change		already known	no		no
Change in Rx		not prev known	other	Gls use	no
no Rx		no vis def	other	gls	no
no Rx		no vis def	no		no
no Rx		no vis def	no		no
no Rx		no vis def	no		no
Replace FWT		no vis def	no		no

no Rx	no vis def	no		no
no Rx	no vis def	no		no
no Rx	no vis def	no		no
no Rx	no vis def	no		no
First time	not prev known	no		no
no Rx	no vis def	other	gls	no
no Rx	no vis def	no		no
no Rx	no vis def	no		no
no Rx	no vis def	no		no
no Rx	no vis def	no		no
no Rx	no vis def	no		no
no Rx	no vis def	no		no
no Rx	no vis def	no		no
no Rx	no vis def	no		no
no Rx	no vis def	no		no
First time	not prev known	no		no
no Rx	no vis def	other	gls	no
no Rx	no vis def	no		no
no Rx	already known	no		no
First time	not prev known	CVI	CVI	yes
Change in Rx	already known	other	gls	no
no Rx	no vis def	no		no
First time	not prev known	no		no
First time	not prev known	no		no
no Rx	no vis def	no		no
no Rx	no vis def	no		no
no Rx	no vis def	no		no
no Rx	no vis def	no		no
no Rx	no vis def	no		no
no Rx	no vis def	no		no
First time	no vis def	no		no
no Rx	no vis def	no		no
no Rx	no vis def	no		no
no Rx	no vis def	no		no
no Rx	no vis def	no		no
no Rx	no vis def	no		no
no Rx	no vis def	no		no
First time	not prev known	no		no
no Rx	no vis def	CVI	CVI	yes
First time	already known	no		no
no Rx	no vis def	other	gls	no
Change in Rx	already known	no		no
First time	not prev known	other	gls	no
no Rx		other	gls	no
no Rx	no vis def	no		no
no Rx	no vis def	no		no
First time	not prev known	no		no
no Rx	no concerns	no		no
no Rx	no vis def	no		no
no Rx	no vis def	no		no

no Rx	no vis def	no		no
no Rx	already known	no		no
Change in Rx	already known	other	gls	no
First time	already known	CVI + other	gls&CVI	yes
Change in Rx	already known	CVI + other	gls/CVI	yes
First time	not prev known	other	gls	no
First time	already known	other	gls	no
no Rx	no vis def	other	gls	no
no Rx	already known	no		no
no Rx	already known	other	r hemianopia	no
no Rx	no vis def	CVI	suspect CVI	yes
First time	not prev known	no		no
no Rx	not prev known	other	gls	no
Change in Rx	already known	CVI	suspect CVI	yes
no Rx	no concerns	CVI + other	gls ?CVI	yes
no Rx	no concerns	no		no
no Rx	no concerns	no		no
No change	already known	no		no
Change in Rx	already known	CVI	CVI	yes
no Rx	no vis def	other	gls	no
First time	not prev known	no		no
no Rx	no vis def	other	gls	no
no Rx	no vis def	no		no
First time	not prev known	no		no
no Rx	no concerns	no		no
no Rx	not prev known	no		no
First time	not prev known	CVI	CVI?	yes
Replace FWT	already known	other	gls	no
No change	already known	CVI + other	squint/?CVI	yes
no Rx	already known	other	gls	no
no Rx	no concerns	CVI	CVI	yes
no Rx	no concerns	no		no
no Rx	no vis def	no		no
No change	already known	no		no
No change	already known	other	gls	no
no Rx	no vis def	other	gls	no
no Rx	no vis def	no		no
no Rx	already known	no		no
no Rx	no vis def	other	squint	no
First time	already known	no		no
no Rx	no vis def	other	gls/squint	no
First time	not prev known	no		no
no Rx	no concerns	no		no
no Rx	no vis def	no		no
no Rx	no vis def	no		no
No change	already known	other	glasses	no
no Rx	no vis def	no		no
First time	not prev known	other	glasses	no
no Rx	no vis def	no		no
Change in Rx	already known	other	glasses	no

no Rx		no vis def	no		no
no Rx		no vis def	no		no
Change in Rx		already known	CVI + other	glasses/CVI?	yes
no Rx		no vis def	no		no
no Rx		no vis def	no		no
no Rx		no concerns	no		no
No change		already known	other	ROP/glasses	no
no Rx		already known	CVI	CVI	yes
no Rx		no vis def	no		no
no Rx		no vis def	no		no
no Rx		no vis def	no		no
First time		not prev known	other	glasses/nyst	no
No change		already known	CVI	CVI	yes
First time		not prev known	other	glasses	no
no Rx		no vis def	no		no
No change		already known	CVI + other	CVI/glasses	yes
Change in Rx		already known	other	glasses	no
no Rx		no vis def	no		no
no Rx		no vis def	no		no
no Rx		no vis def	no		no
no Rx		no vis def	no		no
Change in Rx		already known	other	glasses	no
First time	Ophthalmol	not prev known	CVI + other	glasses/CVI	yes
Change in Rx	Ophthalmol	already known	other	glasses	no
no Rx		no vis def	no		no
No change		already known	CVI + other	glasses/CVI	yes
no Rx		no vis def	no		no
First time		not prev known	other	glasses	no
no Rx		no vis def	no		no
no Rx		already known	CVI	CVI	yes
No change		already known	CVI + other	CVI/glasses	yes
no Rx		already known	CVI	CVI	yes
no Rx		no vis def	no		no
no Rx		no vis def	no		no
First time		not prev known	other	glasses	no
no Rx	Orthoptist	not prev known	no		no
No change		already known	CVI + other	CVI/glasses	yes
no Rx		no vis def	no		no
no Rx		no vis def	no		no
no Rx	Orthoptist	already known	no		no
no Rx		no vis def	no		no
no Rx		no vis def	no		no
Change in Rx	Orthoptist	already known	other	glasses	no
no Rx	Orthoptist	not prev known	no		no
no Rx		no vis def	no		no
no Rx	Orthoptist	already known	no		no
no Rx		no vis def	no		no
no Rx		no vis def	no		no
no Rx		no vis def	no		no
First time		no vis def	other	glasses	no

no Rx		no vis def	no		no
no Rx		no vis def	no		no
no Rx		no vis def	no		no
No change		already known	CVI	CVI	yes
no Rx		no vis def	no		no
no Rx		no vis def	no		no
no Rx	GP	no vis def	no		no
no Rx		no vis def	no		no
no Rx		no vis def	no		no
no Rx		no vis def	no		no
No change		already known	other	nystagmus,glasse	no
no Rx		no vis def	no		no
Change in Rx		already known	other	high ref error	no
Change in Rx		already known	no		no
no Rx		no vis def	no		no
no Rx		no concerns	no		no
no Rx		no vis def	no		no
First time		not prev known	CVI	CVI	yes
no Rx		no vis def	no		no
no Rx		already known	CVI	CVI	yes
no Rx		no vis def	no		no
Change in Rx	Ophthalmol	already known	CVI	cvi	yes
no Rx		not prev known	other	Hemianopia	no
no Rx		no vis def	no		no
no Rx		no vis def	no		no
no Rx		no vis def	no		no
no Rx		no vis def	no		no
no Rx		no vis def	no		no
no Rx		no vis def	no		no
no Rx		no concerns	no		no
First time		not prev known	no		no
First time		already known	no		no
no Rx		no concerns	no		no
no Rx		no vis def	no		no
no Rx		no vis def	no		no
no Rx		already known	other	Nystagmus/Poor	no
no Rx		no vis def	no		no
no Rx		no vis def	no		no
First time		not prev known	no		no
no Rx		no vis def	no		no
No change		already known	other	reduced correcte	no
no Rx	GP	no vis def	no		no
No change		already known	no		no
no Rx		no vis def	no		no
no Rx	GP	no vis def	no		no
no Rx		no concerns	no		no
no Rx		no vis def	no		no
no Rx		no vis def	no		no
First time		not prev known	no		no
no Rx		no concerns	no		no

no Rx		no vis def	no		no
No change		already known	no		no
no Rx		no vis def	no		no
no Rx		no vis def	no		no
Change in Rx		already known	no		no
Change in Rx		already known	no		no
no Rx		not prev known	other	hemianopia	no
no Rx		already known	other	Hemianopia	no
no Rx		no vis def	no		no
no Rx		no vis def	no		no
no Rx		no vis def	no		no
no Rx		no concerns	no		no
no Rx		no concerns	no		no
Replace FWT		already known	CVI	CVI?	yes
no Rx		no vis def	no		no
no Rx		no vis def	no		no
Change in Rx		already known	no		no
no Rx		already known	no		no
no Rx		no vis def	no		no
no Rx		no vis def	no		no
Change in Rx		already known	no		no
no Rx		no concerns	no		no
no Rx		no concerns	no		no
no Rx		no vis def	no		no
no Rx		no concerns	no		no
no Rx		no vis def	no		no
no Rx		no vis def	no		no
no Rx		no vis def	no		no
Change in Rx		already known	no		no
No change		already known			no
no Rx		already known			no
no Rx		already known			no
no Rx		no concerns			no
First time	Orthoptist	vis def no info			no
No change		vis def no info	other	gls	no
no Rx	Orthoptist	no concerns	no		no
no Rx		no concerns	no		no
no Rx	Orthoptist	no concerns	no		no
no Rx		no concerns	no		no
No change		vis def no info	other	gls	no
no Rx	Orthoptist	no concerns	no		no
no Rx	Orthoptist	no concerns			no
no Rx	Orthoptist	no concerns	no		no
no Rx		already known	no		no
no Rx		no concerns	no		no
no Rx		no concerns	no		no
no Rx		already known	no		no
no Rx		vis def no info	no		no
no Rx		already known	no		no
no Rx		no concerns	no		no

no Rx			CVI	cvi	yes
No change		already known	other	gls	no
no Rx		no concerns	no		no
no Rx		no concerns			no
No change		already known	other	gls	no
no Rx		no concerns			no
no Rx		no vis def	no		no
No change		not prev known	other	vi	no
First time		not prev known	other	gls	no
no Rx		no concerns	no		no
no Rx		already known	no		no
no Rx		already known	no		no
No change		already known	other	gls	no
First time		not prev known	other	gls no no ASD<br>CVI CVI	yes
no Rx		no concerns	CVI + other	gls,cvi	yes
no Rx	Orthoptist	no concerns	CVI	cvi	yes
no Rx		no concerns	no		no
no Rx		no concerns	no		no
no Rx		no concerns	CVI	cvi	yes
no Rx		not prev known	other	gls	no
Change in Rx		not prev known	no		no
no Rx		no concerns	other	gls	no
First time		vis def no info	other	gls	no
First time		vis def no info			no
no Rx		no concerns			no
no Rx		no concerns	CVI	CVI	yes
no Rx		no concerns	other	vi	no
no Rx	GP	not prev known	other	gls	no
Change in Rx		vis def no info	no		no
no Rx			no		no
No change		vis def no info	no		no
Change in Rx		vis def no info	no		no
no Rx			no		no
no Rx			no		no
No change		vis def no info	no		no
Change in Rx		vis def no info	no		no
Change in Rx		vis def no info	no		no
Change in Rx		vis def no info	no		no
no Rx		no concerns	no		no
First time			no		no
no Rx			no		no
Change in Rx		vis def no info	CVI	CVI Sus	yes
no Rx			no		no
no Rx			no		no
no Rx			CVI	CVI Sus	yes
Change in Rx		vis def no info	no		no
Change in Rx		vis def no info	CVI	CVI sus	yes
Change in Rx		vis def no info	CVI	CVI sus	yes

no Rx			no		no
no Rx			no		no
no Rx		vis def no info	no		no
Change in Rx		vis def no info	no		no
Change in Rx			no		no
Change in Rx			no		no
Change in Rx		vis def no info	no		no
Change in Rx		vis def no info	no		no
Change in Rx			no		no
Change in Rx			no		no
no Rx			no		no
Change in Rx		vis def no info	no		no
Change in Rx		vis def no info	CVI	CVI sus	yes
Change in Rx		vis def no info	no		no
Change in Rx			other		no
no Rx		vis def no info	CVI	CVI sus	yes
no Rx			no		no
Change in Rx			no		no
Change in Rx			CVI	CVI	yes
no Rx		already known	no		no
Replace FWT		already known	other	SSI	no
no Rx		already known	no		no
no Rx		no concerns	no		no
no Rx		no concerns	other	VI"	no
no Rx		no concerns	no		no
First time	GP	not prev known	other	gls	no
no Rx		not prev known			no
no Rx		no concerns	no		no
no Rx		no concerns	no		no
no Rx		no concerns	no		no
no Rx		no concerns	no		no
no Rx		no concerns	no		no
no Rx		no concerns	no		no
no Rx		no concerns	other	gls	no
No change		vis def no info	other	gls	no
No change		vis def no info	no		no
no Rx		no concerns	no		no
no Rx		no concerns	no		no
no Rx		no concerns	CVI + other	gls CVI	yes
First time		vis def no info	other	gls	no
First time		not prev known	no		no
no Rx		not prev known	no		no
no Rx		no vis def	other	gls	no
First time		not prev known	no		no
no Rx		no vis def	no		no
no Rx		no vis def	other	gls	no
Replace FWT		already known	other	gls	no
Change in Rx		already known	no		no
no Rx		no vis def	no		no
First time		already known	no		no

no Rx		no vis def	no		no
no Rx		no vis def	no		no
no Rx		no vis def	no		no
no Rx		no vis def	other	cataracts	no
no Rx		already known	no		no
no Rx		no vis def	no		no
no Rx		no vis def	other	gls	no
Replace FWT		already known	CVI + other	gls/CVI	yes
First time		not prev known	other	gls	no
Change in Rx		already known	CVI	CVI?	yes
Change in Rx	Ophthalmol	already known	no		no
First time		not prev known	no		no
no Rx		no vis def	CVI	CVI	yes
no Rx		not prev known	no		no
no Rx		no vis def	CVI + other	CVI/gls	yes
Replace FWT		already known	no		no
no Rx		no vis def	no		no
no Rx		no vis def	no		no
no Rx		no vis def	no		no
no Rx		no vis def	no		no
First time		not prev known	no		no
no Rx		no concerns	no		no
no Rx		no concerns	no		no
no Rx		no vis def	no		no
First time		not prev known	no		no
no Rx		no concerns	no		no
First time		not prev known	no		no
no Rx		no vis def	no		no
no Rx		no vis def	CVI	CVI	yes
no Rx		already known	no		no
no Rx		no concerns	no		no
no Rx		no vis def	no		no
no Rx		no concerns	no		no
no Rx		no concerns	no		no
no Rx		no concerns	no		no
First time		not prev known	no		no
First time		not prev known	no		no
no Rx		no concerns	no		no
no Rx		no vis def	no		no
no Rx		no concerns	no		no
no Rx		no concerns	no		no
no Rx		no concerns	no		no
no Rx		no concerns	no		no
no Rx		no concerns	no		no
Change in Rx		already known	no		no
First time		not prev known	CVI	CVI	yes
no Rx		already known	no		no
Change in Rx		already known	no		no
First time	GP	not prev known	no		no
no Rx		no vis def	no		no

no Rx	no concerns	no	no
no Rx	no concerns	no	no
First time	not prev known	no	no
no Rx	no vis def	no	no
no Rx	no concerns		

ASD

no ASD
ASD
no ASD
ASD
no ASD
no ASD
no ASD

no ASD
no ASD
no ASD
no ASD
no ASD
no ASD
ASD
no ASD
ASD
no ASD
ASD
no ASD
no ASD
no ASD
no ASD
no ASD
no ASD
no ASD

ASD
ASD
no ASD
no ASD
no ASD
no ASD

no ASD
no ASD
ASD
ASD
no ASD
no ASD
no ASD
ASD
ASD

ASD

ASD
ASD
ASD
no ASD
ASD
no ASD

ASD
no ASD
no ASD
ASD
ASD
ASD
ASD
ASD
ASD
ASD

no ASD
ASD

ASD

ASD
no ASD

no ASD
ASD
ASD
ASD

no ASD
ASD

ASD

ASD
no ASD
ASD
ASD
ASD
no ASD
ASD
ASD

ASD
ASD
ASD

no ASD
no ASD
no ASD
no ASD

ASD
no ASD
no ASD
ASD
no ASD
no ASD
no ASD
ASD
no ASD
no ASD
no ASD
no ASD
ASD
no ASD
no ASD
no ASD
no ASD
no ASD
no ASD
ASD
ASD
ASD
ASD
ASD

ASD
no ASD
ASD
no ASD
ASD
no ASD
no ASD
ASD
ASD

no ASD
ASD

ASD

ASD

ASD

no ASD
ASD
no ASD
ASD
no ASD
no ASD
no ASD
no ASD
no ASD
no ASD
ASD
ASD
no ASD
no ASD
no ASD
no ASD
no ASD
no ASD
no ASD
no ASD
no ASD
ASD
no ASD
no ASD
no ASD
no ASD

no ASD
no ASD
no ASD
no ASD
no ASD
ASD
no ASD
no ASD
ASD
ASD
no ASD
ASD
no ASD

no ASD
ASD
no ASD
no ASD
no ASD
no ASD

ASD
no ASD
no ASD
no ASD
ASD
ASD
no ASD
no ASD
no ASD
no ASD
no ASD
no ASD
no ASD
ASD
no ASD
no ASD
ASD
no ASD
no ASD
ASD

no ASD
no ASD

ASD
ASD
ASD
no ASD
ASD
ASD
ASD
no ASD
ASD
ASD
ASD
ASD
ASD
ASD
ASD
ASD
no ASD
no ASD

no ASD
no ASD
ASD
ASD
ASD
ASD
ASD

ASD
no ASD
ASD
no ASD
no ASD

no ASD
no ASD
no ASD
no ASD

no ASD
no ASD
no ASD
ASD
no ASD
no ASD
ASD

ASD
no ASD
ASD
no ASD
no ASD
no ASD
no ASD

no ASD
no ASD
no ASD
no ASD
no ASD
no ASD
ASD
no ASD
ASD
ASD
no ASD
ASD

no ASD
ASD
ASD
no ASD
no ASD
no ASD
no ASD

no ASD

no ASD
no ASD
ASD
no ASD
no ASD

ASD
no ASD
no ASD
no ASD
no ASD
no ASD
ASD

no ASD
ASD
ASD
no ASD
no ASD
no ASD
no ASD
ASD
ASD
no ASD
no ASD
no ASD
no ASD
no ASD
no ASD
no ASD

no ASD
ASD
no ASD
no ASD

ASD
no ASD
ASD
ASD
no ASD
no ASD
no ASD
no ASD
no ASD
no ASD
no ASD
ASD
no ASD
no ASD

ASD
ASD
ASD
ASD
no ASD
no ASD
no ASD
ASD
ASD
ASD
ASD
no ASD
no ASD
ASD
ASD
ASD
no ASD
ASD
ASD
ASD
ASD
ASD
no ASD
ASD
ASD
no ASD
ASD
no ASD
ASD
ASD
ASD
ASD
ASD
ASD
ASD
ASD
ASD
no ASD
ASD
no ASD
ASD
ASD
ASD
no ASD
no ASD
ASD
ASD
ASD
no ASD

no ASD
no ASD
ASD
no ASD
no ASD
ASD
ASD
no ASD
no ASD
ASD
no ASD
no ASD
no ASD
no ASD
no ASD
no ASD
ASD
no ASD
ASD
no ASD
ASD
no ASD
no ASD
ASD
ASD
ASD
ASD
no ASD
no ASD
no ASD
ASD
ASD
no ASD
no ASD
no ASD

no ASD
no ASD
ASD
no ASD
ASD
ASD
no ASD
no ASD
ASD
no ASD
no ASD
no ASD
no ASD
no ASD

ASD
no ASD
no ASD
ASD
ASD
ASD
ASD
no ASD
ASD

ASD
ASD
no ASD
ASD
ASD
no ASD
ASD
no ASD
ASD
ASD
no ASD
ASD
ASD
no ASD
ASD
no ASD
ASD
ASD
no ASD
no ASD
no ASD
no ASD
no ASD
no ASD
ASD
ASD
ASD

ASD
no ASD
no ASD
ASD
no ASD
no ASD
ASD
no ASD
no ASD
no ASD
ASD
ASD

no ASD
no ASD
no ASD
ASD
ASD
ASD
ASD
no ASD
ASD
no ASD
no ASD
ASD

ASD

no ASD
ASD
no ASD
no ASD
ASD
ASD
ASD
ASD
ASD
ASD
no ASD
no ASD

ASD
ASD
ASD
no ASD
no ASD
no ASD
ASD
no ASD
no ASD
ASD
ASD
ASD

ASD
ASD

no ASD
ASD
ASD
ASD
ASD
no ASD

ASD
ASD
ASD

ASD
ASD
ASD
ASD
ASD
ASD
ASD
ASD
ASD

ASD
no ASD
ASD
no ASD

ASD
ASD
ASD

ASD
ASD
ASD
ASD
ASD

ASD
no ASD
ASD
no ASD
ASD
ASD
no ASD

ASD
no ASD

no ASD
no ASD
no ASD
no ASD
ASD
no ASD
no ASD
ASD
no ASD
no ASD

no ASD
no ASD
no ASD
ASD
ASD
ASD
no ASD

ASD
ASD
ASD
ASD
no ASD
no ASD
no ASD
no ASD
no ASD
ASD
ASD

ASD
no ASD
no ASD
no ASD
no ASD
no ASD
ASD

ASD
ASD
ASD

ASD
no ASD
no ASD
no ASD
ASD
ASD

ASD
ASD
no ASD
ASD
ASD
ASD
no ASD
no ASD
ASD
no ASD
ASD
ASD
no ASD
ASD
ASD
no ASD
ASD
ASD
ASD
no ASD
ASD
no ASD
no ASD
no ASD
ASD
no ASD
ASD
ASD
ASD
no ASD
no ASD
no ASD
ASD
ASD
ASD
ASD
no ASD
ASD
ASD
ASD
ASD
ASD
ASD
ASD
ASD
ASD
ASD
no ASD
ASD
ASD

ASD
ASD
ASD
no ASD
ASD

no ASD
ASD
ASD
ASD
ASD
ASD
ASD
no ASD
ASD
ASD
ASD
no ASD
ASD
no ASD
ASD
no ASD
ASD
ASD
ASD
ASD
ASD
no ASD
ASD
ASD
no ASD
ASD
ASD
no ASD
ASD
no ASD
no ASD

ASD
no ASD
no ASD
ASD
no ASD
ASD
no ASD
ASD
ASD
ASD
ASD
ASD

ASD
no ASD
ASD
ASD
ASD
no ASD
no ASD
no ASD
ASD
ASD
no ASD
ASD
ASD
no ASD
no ASD
ASD
ASD
no ASD
no ASD
no ASD
no ASD
ASD
ASD
no ASD
ASD
ASD
no ASD
ASD
ASD
ASD
no ASD
no ASD
ASD
no ASD
ASD
ASD
ASD
ASD
ASD
no ASD
ASD
no ASD
ASD
ASD
ASD
ASD
no ASD
no ASD
ASD
ASD

no ASD

ASD
ASD
no ASD
ASD
no ASD
no ASD
no ASD
ASD
no ASD
no ASD
no ASD

ASD
ASD
ASD
ASD
ASD
no ASD
ASD
no ASD
no ASD

ASD
ASD
no ASD
ASD
no ASD
no ASD

ASD
no ASD
ASD
ASD

no ASD

ASD
no ASD
no ASD
no ASD
no ASD

no ASD
ASD

no ASD

ASD

ASD
no ASD

ASD
no ASD

no ASD

ASD
no ASD
no ASD
ASD
ASD
ASD
no ASD
no ASD
no ASD
no ASD
ASD
no ASD
no ASD
ASD
ASD
ASD

ASD
ASD
no ASD
ASD
no ASD

ASD
ASD

no ASD
ASD
ASD
ASD
ASD
ASD
ASD
ASD
no ASD
ASD
ASD
no ASD

ASD
ASD
ASD
no ASD
no ASD
no ASD
no ASD
no ASD
no ASD
no ASD
ASD
no ASD
no ASD
ASD
no ASD
ASD
ASD
ASD
ASD
no ASD
ASD
ASD
no ASD
ASD
ASD
ASD
no ASD
ASD
no ASD
ASD
ASD
ASD
ASD
ASD
ASD
ASD
ASD
no ASD
ASD
ASD
ASD
ASD
ASD
ASD
ASD
no ASD
no ASD
no ASD
no ASD
ASD

ASD
ASD
ASD
ASD
